# Phylogeographic evaluation of the effectiveness of Canadian travel restrictions in reducing SARS-CoV-2 variant importations and burden

**DOI:** 10.1093/ve/veaf077

**Published:** 2025-10-11

**Authors:** Angela McLaughlin, Vincent Montoya, Rachel L Miller, Canadian COVID-19 Genomics Network (CanCOGeN) Consortium, Brinkman Fiona, Anwar Zohaib, Côté Marceline, Fiume Marc, Gilbert Laura, Gill Erin, Gordon Paul, Joly Yann, Moreira Sandrine, Mubareka Samira, Prystajecky Natalie, Tanner Jennifer, Domselaar Gary Van, Zahariadis Phot, Michael Worobey, Jeffrey B Joy

**Affiliations:** Molecular Epidemiology and Evolutionary Genetics, British Columbia Centre for Excellence in HIV/AIDS, 608-1081 Burrard Street, Vancouver, BC V6Z 1Y6, Canada; Bioinformatics, University of British Columbia, 315- 2185 East Mall, Vancouver, BC V6T 1Z4, Canada; Molecular Epidemiology and Evolutionary Genetics, British Columbia Centre for Excellence in HIV/AIDS, 608-1081 Burrard Street, Vancouver, BC V6Z 1Y6, Canada; Molecular Epidemiology and Evolutionary Genetics, British Columbia Centre for Excellence in HIV/AIDS, 608-1081 Burrard Street, Vancouver, BC V6Z 1Y6, Canada; Department of Ecology and Evolution, University of Arizona, PO Box 210088, Tucson, AZ 85721, United States; Molecular Epidemiology and Evolutionary Genetics, British Columbia Centre for Excellence in HIV/AIDS, 608-1081 Burrard Street, Vancouver, BC V6Z 1Y6, Canada; Bioinformatics, University of British Columbia, 315- 2185 East Mall, Vancouver, BC V6T 1Z4, Canada; Department of Medicine, University of British Columbia, 317 - 2194 Health Sciences Mall, Vancouver, BC V5Z 1M9, Canada

**Keywords:** phylogenetics, genomic epidemiology, SARS-CoV-2, COVID-19, variant, phylogeography

## Abstract

Evaluating travel restriction effectiveness in mitigating infectious disease burden, exemplified by COVID-19, is critical for informing pandemic response policy, yet methodologies and results evaluating their effectiveness vary considerably. We hypothesized Canadian COVID-19 travel restrictions, including flight bans and enhanced screening, targeting focal source countries where SARS-CoV-2 variants of concern (VOCs) Alpha, Beta, Gamma, Delta, and Omicron were first identified, were variably effective towards averting introductions and case burden. We conducted a retrospective observational study using all the publicly available SARS-CoV-2 sequences and COVID-19 diagnoses up to March 2022, after which polymerase chain reaction (PCR) testing and surveillance sequencing decreased. Average daily variant cases were estimated across global regions and Canadian provinces, which informed subsampling probabilities for sequences for up to 50 000 sequences for VOCs and variants of interest from late 2020 to early 2022. Maximum likelihood phylogeographic methods were used to infer Canadian SARS-CoV-2 sublineages and singletons, representing international viral introductions with and without domestically sampled descendants. Reduction of sublineage and singleton introduction rates and proportional contributions from focal sources were quantified following interventions’ introductions. Sublineages and cases averted *via* VOC travel restrictions were estimated based on sublineages’ introduction rates and growth characteristics prior to restrictions. Our results suggest that across VOCs subject to targeted travel restrictions, approximately 995 (841–1151) introductions may have been prevented, accounting for an averted burden of 971 371 (321 204–1 004 575) cases, 10 685 (3533–11 050) hospitalizations, and 561 (185–580) deaths, largely accounted for by the Delta-related India flight ban. However, these estimates represent an upper bound of effectiveness if any assumptions were violated, including that introductions can be treated as independent when susceptibility is high, averted introductions mirror characteristics of observed introductions, and that travel restrictions caused sustained changes in travel behaviour. Travel restrictions were most effective when implemented rapidly following variant emergence, during exponential case growth in the focal source country, and concurrent with limited domestic and global circulation. Our analyses suggest that COVID-19 travel restrictions, particularly flight suspensions, mitigated variant case burden when global circulation was limited and case burden was high in the focal source, and highlight their value in future pandemic response, although public health benefits must be weighed against socioeconomic costs.

## Introduction

Emergence and successive sweeps of SARS-CoV-2 variants of concern (VOCs) with elevated transmissibility, immune evasion, and/or virulence (Konings et al. 2021) have challenged the effectiveness of COVID-19 nonpharmaceutical interventions (NPIs) and vaccines. Reconstructing the emergence and spread of VOCs can illuminate NPI effectiveness in mitigating viral introductions and burden, informing policy for ongoing and future pandemics. Phylogenetic analyses of SARS-CoV-2 genomes have been applied to infer viruses’ geographic and temporal origins (Worobey et al. 2020, du Plessis et al. 2021, McLaughlin et al. 2022), in addition to informing dynamic nomenclature systems (Hadfield et al. 2018, Rambaut et al. 2020, O’Toole et al. 2021a), estimating key epidemiological metrics such as the effective reproduction number (Nadeau et al. 2022), and corroborating or refuting epidemiological linkage through contact tracing (Douglas et al. 2021, Anderegg et al. 2023). Thus, phylogenetic inference of the importation dynamics of SARS-CoV-2 VOCs provides an empirical basis to evaluate travel restriction effectiveness.

Cross-border health measures, or travel restrictions, are a class of NPI applied to mitigate pandemic burden by managing the movement of people across jurisdictions and can be characterized by policy goal, type of movement, level of jurisdiction, stage of journey, and degree of restrictiveness (Lee et al. 2021). For COVID-19, travel restrictions were applied broadly or targeting particular countries and included restricted entry of foreign nationals, flight bans, enhanced screening, entry requirements such as testing or vaccination, and quarantine requirements.

Evidence and methodologies evaluating the effectiveness of travel COVID-19 restrictions have been variable (Burns et al. 2021, Grépin et al. 2021, Talic et al. 2021, Banholzer et al. 2022, Lison et al. 2023). In a review of early COVID-19 travel restrictions, eight of nine studies found travel restrictions were associated with reduced imported and exported cases, with variation due to differences in travel volumes, timing, stringency, and connectedness (Burns et al. 2021). A systematic review of COVID-19 travel–related measures found broad agreement that early measures against travel from Wuhan reduced transmission in mainland China and early implementation was a determinant of measures’ success (Grépin et al. 2021)*.*

Phylogeographic reconstruction of SARS-CoV-2 introductions has been applied to evaluate travel restrictions. International travel restrictions in 2020 were associated with reduced importation events in Canada (McLaughlin et al. 2022) and the United Arab Emirates (Henschel et al. 2022), and delayed onset of community transmission in Zimbabwe (Mashe et al. 2021). International travel restrictions and stringent domestic interventions within a zero-COVID policy in New Zealand eliminated community transmission until the arrival of Delta (Jelley et al. 2022). Relaxation of Swiss control measures alongside increased travel during the summers of 2020 and 2021 was associated with elevated importations and sustained local epidemic growth (Reichmuth et al. 2022). Mandatory hotel quarantines in England in response to Delta reduced onward transmission, but subsequent NPI relaxation drove rapid transmission of early introductions (McCrone et al. 2022). A small proportion of introductions often account for the majority of local cases: a single introduction to Boston resulted in hundreds of thousands of cases *via* superspreading at a conference (Lemieux et al. 2021). However, viral establishment prior to an intervention limits its effectiveness—for instance, US restrictions against travel from Brazil did not prevent establishment of Gamma in New York City, as the majority of Gamma sublineages were introduced at least 2 weeks prior from within the USA (Vasylyeva et al. 2022). In Hong Kong, border control measures were insufficient to prevent outbreaks during a period of lower stringency (Gu et al. 2022). In South Korea, international traveller quarantine in 2020 reduced introductions and spread of SARS-CoV-2, but control was incomplete (Kwon et al. 2021). Differential relationships between reduced importations and reduced transmission are affected by local incidence (Lemey et al. 2021).

Mathematical models have also been widely applied to quantify travel measures’ effectiveness. Counterfactual simulations indicated earlier implementation of travel measures in the first wave of COVID-19 would have averted cases and deaths (Pei et al. 2020). Stochastic branching processes were applied to simulate outbreaks averted by the *cordon sanitaire* imposed in Chinese cities in early 2020, which was deemed ineffective at reducing cumulative incidence or time to sustained transmission, despite reducing exports from Wuhan (Quilty et al. 2020). A semi-mechanistic Bayesian hierarchical model across 20 countries suggested that bans on large gatherings were the most effective NPI, while border closures were associated with variable and uncertain cases reduction (Banholzer et al. 2021). Compartmental models of early COVID-19 indicated sustained travel restrictions to and from China were more effective alongside a reduction of domestic transmission (Chinazzi et al. 2020). During the 2003 SARS-CoV epidemic, travel restrictions reduced travel but were too weak and tardy to impact global spread, whereas case isolation was effective because infectiousness peaked after symptom onset (Hollingsworth et al. 2006). Compartmental models fit to early COVID-19 showed wide variability in jurisdictions’ leeway for reopening borders in relation to the probability that contact rates were below the threshold for epidemic growth (Anderson et al. 2021). Across 142 countries, travel bans and lockdowns reduced *R*_e_ (Koh et al. 2020). Stochastic meta-population models of global spread of early SARS-CoV-2 found the 2020 travel ban on China significantly reduced importations and cases in Australia, as well as delayed the outbreak, while the bans on Iran, South Korea, and Italy were ineffective (Adekunle et al. 2020). This selection of studies highlights wide variability in COVID-19 interventions’ effectiveness depending on stringency, criteria, and circumstance.

**Figure 1:**
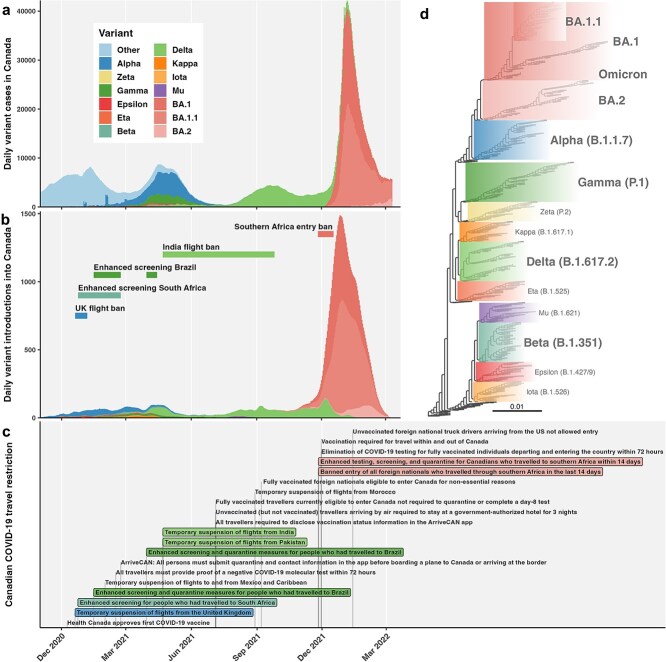
Epidemiological dynamics of SARS-CoV-2 variants in Canada. (a) Estimated daily cases of variants of concern (VOCs) and interest (VOIs) in Canada from 1 November 2020 to 22 March 2022. (b) Variant introductions into Canada, overlaid with duration of VOC-specific travel restrictions. (c) Timeline of Canadian federal COVID-19 travel restrictions’ implementation, including entry requirements and variant enhanced screening and flight bans, between November 2020 and March 2022. Additional details in Supplementary. (d) Maximum likelihood phylogeny of SARS-CoV-2 variants scaled by substitutions per site.

Canadian COVID-19 travel restrictions including flight bans and enhanced screening were implemented targeting focal regions where VOCs were first detected (Fig. 1b), but their relative effectiveness in limiting introductions and case burden has not been characterized and is important to understand, given their unintended economic and social consequences, such as supply chain disruption and family separation (Klinger et al. 2021). Previous evidence suggested that the March 2020 entry ban for foreign nationals reduced viral importations into Canada but was insufficient to prevent new SARS-CoV-2 sublineages from seeding the second wave (McLaughlin et al. 2022). We hypothesized that Canadian SARS-CoV-2 variant travel restrictions were associated with reduced introduction rates from focal sources and subsequently fewer cases. To test these hypotheses on the effectiveness of variant travel restrictions, we inferred phylogeographic models of SARS-CoV-2 variant dispersal into Canada. We subsampled publicly available Global Initiative on Sharing All Influenza Data (GISAID) sequences available up to 22 March 2022 proportionally to estimated variant cases for each Canadian province or global region. We then inferred maximum likelihood phylogenetic trees for up to 50 000 sequences per subsample and ancestral geographies for VOCs and variants of interest (VOIs) to estimate the timing, origins, destination, and spread of variant introductions into Canada. We further estimated the effectiveness of flight bans for Alpha, Delta, and Omicron variants, and enhanced screening for Gamma and Beta variants, in averting viral importations and cases using a counterfactual approach, whereby expected introduction rates in the absence of restrictions were predicted based on VOC cases in focal sources and travel volume. This approach is premised on the assumptions that averted introductions would have resulted in a similar number of descendants as observed introductions for each variant, introductions are independent of each other in a primarily susceptible population, and that the relationship between variant cases in the focal source and introductions preceding restrictions would be sustained in the absence of restrictions; violation of these assumptions lends additional uncertainty beyond the limits reported. Monitoring and analysing the emergence, introduction, and spread of viral sublineages is crucial to guide public health responses to ongoing and future epidemics.

## Methods

### Ethics approval

Ethical approval for this study was granted by the University of British Columbia Providence Healthcare Research Ethics Board (H23-02908).

### Timeline of Canadian COVID-19 variant travel restrictions

Federal COVID-19 travel restrictions in Canada in the early VOC era (up to Omicron BA.1 and BA.2) were summarized from November 2020 up to March 2022, including national entry requirements and variant travel restrictions (Fig. 1d). Events were collated from the Canadian Institute for Health Information (CIHI 2021), news releases from the Government of Canada, and a timeline of border measures (Canada Border Services Agency 2021b). An expanded list of key events is in the Supplementary.

### Sequence data and curation

All available SARS-CoV-2 genome sequences and associated metadata (*n* = 9 487 106, with 298 892 collected in Canada) were downloaded from GISAID on 22 March 2022 (Shu and McCauley 2017, Khare et al. 2021). GISAID clade partitions were maintained throughout data cleaning to reduce computational burden. Sequences were excluded if they were listed on the Nextstrain exclude list updated on the day of data download (*n* = 8782) (Hadfield et al. 2018, Bedford 2021), duplicated IDs (*n* = 4284), from a nonhuman host (*n* = 6803), environmental samples (*n* = 4907), or had incomplete dates outside of samples from Canada (*n* = 218 233). For Canadian sequences with incomplete dates within known months, days were randomly sampled. If only the year was provided, the sequence was discarded.

Sequences were aligned to Wuhan-Hu-1 (GenBank ID: MN908947.3) using the viralMSA wrapper of minimap2 (Li 2018, Moshiri 2020). Sequences were excluded if ambiguous sites or gaps exceeded 10% (*n* = 7968 and *n* = 657 063, respectively), resulting in 8 585 792 total clean sequences (274 568 from Canada). Pango lineages in the GISAID metadata were called using pangolin v3.1.20 and pangoLEARN data release 28 February 2022 (Rambaut et al. 2020, O’Toole et al. 2021a).

Clean sequences were grouped by variant (Table S3): Alpha (B.1.1.7, Q.^*^), Beta (B.1.351), Gamma (P.1), Delta (B.1.617.2, AY.^*^), Epsilon (B.1.429 and B.1.427), Zeta (P.2), Eta (B.1.525), Iota (B.1.526), Kappa (B.1.617.1), Mu (B.1.621), and Omicron (separately: BA.1, BA.1.1, and BA.2). Any sequence not identified as a variant was grouped into ‘Other’. Variants with fewer than 100 sequences from Canada were excluded (Lambda, Theta, GH/490R, and BA.3). The primary manuscript focuses on VOCs, Alpha, Beta, Gamma, Delta, and Omicron BA.1, BA.1.1, and BA.2, for which there were targeted travel restrictions. Omicron was split into BA.1, BA.1.1, and BA.2 lineages as their spatiotemporal origins into Canada differed. The earliest global sample collection dates for each variant were obtained from cov-lineages.org (O’Toole et al. 2021b) and outbreak.info (Gangavarapu et al. 2023), and the earliest Canadian dates were pulled from clean GISAID data (Table S1). Canadian sequences with collection dates preceding the earliest global date for each variant were removed.

### Estimating variant cases by Canadian province and global region

Canadian daily new COVID-19 diagnoses by province were obtained from the Public Health Agency of Canada (PHAC 2021). Global daily new diagnoses by country were obtained from the R package coronavirus (Krispin and Byrnes 2020), which pulls data from the Johns Hopkins University Center for Systems Science and Engineering Coronavirus repository. Rolling 7-day averages of daily new diagnoses were calculated. For global regions, countries were grouped into continents unless their sequence contributions were within the top 95th percentile, which included Brazil, Denmark, France, Germany, India, Japan, South Africa, Spain, Sweden, Switzerland, the UK, and the USA. Population-normalized incidence and COVID-19 mortality were compared across provinces and territories (Fig. S2).

Average daily variant cases overall in Canada and globally (Fig. S3), and for all Canadian provinces and global regions were calculated as the product of average daily new diagnoses and average daily variant frequency based on clean GISAID sequences (Figs S4 and S6). Total monthly contributions to variant cases were calculated for each province and global region; then, tallies were re-grouped by variant to calculate geographies’ proportional contributions to cases of each variant for each calendar month (Figs S5 and S7). Monthly proportional contributions to variant cases were used to inform the probability of subsampling a sequence from a given geography and month.

### Subsampling data to reduce spatiotemporal bias

For each variant, up to 50 000 sequences were subsampled where available, including 50% from Canada and 50% from other global regions, which is a relative sequence representation that identified the most sublineages compared to other sampling strategies in analyses of the first two waves (McLaughlin et al. 2022). The temporal distribution of sampled sequences reflects the distribution of monthly estimated variant cases for Canada and global regions, with augmented sampling of early months with sparse cases (Fig. S8). We compared relative frequencies of variant cases, sequences available (Table S3), and sequences sampled (Figs S10 and S11). Sequences were subsampled with probabilities equal to the monthly contribution of global regions or Canadian provinces to variant cases, up to the total number of target sequences per month. Subsampling was repeated ten times with replacement. Similar subsampling approaches based on countries’ cases or deaths have been applied elsewhere (Nadeau et al. 2021, Tegally et al. 2023). Additional sequences were sampled from variants’ parental lineages (i.e., lineages B, B.1, and B.1.1 for variant B.1.1.7) to inform the topology and timing of early introductions. For B, Wuhan-hu-1 was sampled, and for other parental lineages, 10 sequences with a collection date preceding the first global variant sample were randomly sampled from the global dataset for each subsample. All 1 752 808 unique subsampled genome sequences from 200 countries and territories were collated into EPI_SET_230510yr (doi: 10.55876/gis8.230510yr) and Supplemental Table provided by GISAID.

### Maximum likelihood phylogenetic inference and ancestral state reconstruction

Problematic sites were censored from sequence alignments prior to phylogenetic inference (de Maio et al. 2020). Approximate maximum likelihood (ML) trees were generated for each variant subsample using FastTree v2.1.11 under a generalized time-reversible (GTR) substitution model and the ‘-fastest’ algorithm (Price et al. 2010). Trees were outgroup-rooted on Wuhan-hu-1 in R package ‘ape’ (Paradis and Schliep 2018). A linear regression of root-to-tip distance versus time was generated for each tree, excluding parental lineage sequences. Tips with absolute value residuals >0.001 or terminal branch lengths longer than 20 mutations were excluded as temporal outliers. Trees were time-scaled using IQ-TREE 2.1.2 with least squares dating (LSD2) (To et al. 2016, Minh et al. 2020), a GTR model with empirical base frequencies, invariant sites, free-rate site heterogeneity with four categories, a relaxed molecular clock with 0.2 variance, and 50 bootstraps to estimate uncertainty of internal branch lengths. Polytomies were randomly resolved.

To infer a phylogeny relating variants (Fig. 1d), 100 sequences for each variant were sampled randomly from those with collection dates in the first 10% of sampling dates available (restricted to early diversity to focus on variants’ relative ancestry). Variants sampled included Alpha, Beta, Gamma, Delta, Omicron (BA.1, BA.1.1, and BA.2 separately), Eta, Epsilon, Iota, Kappa, Mu, and Zeta; 100 samples from wild-type SARS-CoV-2 (‘Other’) and reference sequence Wuhan-hu-1 were also included. An approximate ML tree was inferred using FastTree v2.1.11 under a GTR model and rooted on outgroup, Wuhan-hu-1. The tree was visualized using ggtree (Yu et al. 2016), and clades representing VOCs were expanded.

Phylogeographic reconstruction of internal nodes’ geographic state as Canadian province or global region was conducted using ML discrete ancestral character estimation in R package ape with symmetrical rates (Paradis and Schliep 2018). The highest likelihood state was pulled for each internal node. We defined Canadian sublineages as subtrees descending from Canadian internal nodes preceded by non-Canadian internal nodes, signifying an international introduction resulting in onward sampled transmission. Sublineages’ estimated origins are limited to the common ancestor of sampled viruses and may not include the true introducing case (infected traveller). Singletons were defined as Canadian sequences with non-Canadian parental origin and no sampled descendants; singletons may represent the introducing case or a downstream case with unobserved recent Canadian ancestry. Sublineage and singleton importation rates were summarized as a 7-day rolling mean of importations per week by global region, province of introduction, and Pango lineage. The sum of sublineages and singletons (Table S2) represents a lower limit for the total number of introductions due to subsampling. Rate estimates were reported as the mean across subsamples, with 95% confidence intervals calculated using the *t*-distribution.

The fold reduction of sublineage and singleton importation rates from focal sources were calculated 1–4 weeks after implementation of restrictions versus immediately prior to implementation of the restriction (Fig. 3). The fold reduction in proportion of sublineages originating from focal sources was calculated for periods before versus during restrictions. Statistical significance of fold reductions above or below one was tested using one-sample two-sided *t*-tests with Benjamini and Hochberg–adjusted *P*-values (Ferreira and Zwinderman 2006).

The ratios of introductions to cumulative variant diagnoses in the week immediately preceding restrictions and during the early and late periods of each restriction for observed and predicted were calculated. A similar ratio of imported cases to incidence was applied (Russell et al. 2021), whereby they considered that above 1%, travel restrictions could have an impact on controlling epidemics. Observed introductions were calculated as the sum over that period of daily 7-day average rolling introductions, including singletons and sublineages, from all sources, and cumulative diagnoses were calculated as the sum of the daily estimated variant cases (Table S8). Predicted introductions and diagnoses were calculated as the sum of observed and averted in the specified periods.

### Phylogeographic sensitivity analyses

To assess the robustness of results obtained using ML phylogeography, we compared relative importation rates obtained with Bayesian discrete trait analysis in BEAST v1.10.4 with BEAGLE (Minin and Suchard 2008, Lemey et al. 2009, Ayres et al. 2012, Suchard et al. 2018). Additional details on model selection and priors are in the Supplementary Material. For Alpha, Beta, and Delta variants, we further subsampled 1 of 10 subsamples from the primary analysis up to 500 sequences (250 from each of Canada and global, with parental lineages) using the same subsampling strategy. Tips’ geographic states (or demes) in the model were merged to fewer categories with ‘more’ or ‘less’ demes (Tables S5, S6, S7). We applied the ML phylogeographic pipeline with the same secondary subsamples of *n* = 500 to reconstruct geographies using the same sets of demes as for the BEAST analysis.

We further compared ML results for a range of sample sizes including 50 000 (original analysis), 10 000, 5000, 1000, and 500 sequences. The same subsampling algorithm was applied for each, with 50% of sequences from Canada and global. Comparison metrics included correlation of monthly sequences to cases (Figs S38, S39), fold reduction of viral importation rates (sublineages and singletons, together) from the focal region associated with travel restriction, as well as proportion of importations from the focal region before and during travel restrictions.

### Counterfactual models of introductions and cases averted

We estimated introductions averted due to travel restrictions for each VOC by comparing observed to expected introductions in the absence of travel restrictions using deterministic counterfactual linear models relating introduction rates (sum of sublineages and singletons) to variant cases in the focal source. Models were trained on data 6–10 days preceding the restriction (described for each VOC; Figs S19–S24). Introductions averted are the difference in the areas under the curves of observed and predicted introductions per day during interventions (Fig. 4). Statistics Canada data on international arrivals (Statistics Canada 2023) were used to quantify averted travel volume from focal sources in the absence of restrictions (Figs S14–S18) in corroboration of trends in estimated introduction rates from focal sources.

Cases averted were estimated using stochastic branching processes with epochal reproduction number (*R*_t_), reflecting the daily median R_t_ across sublineages (Figs S25, S26), and variable case ascertainment and sampling (Lloyd-Smith et al. 2005, Althaus 2015, Grubaugh et al. 2017, Riou and Althaus 2020, Reichmuth et al. 2022). For each variant, we simulated 10 000–20 000 outbreaks, which ran until infected cases were zero, cumulative infected cases exceeded the maximum expected outbreak size, or duration (time from first to last case) exceeded the maximum observed sublineage lifespan. Each introduction seeds a branching process where the number of descendants of each infected case is drawn from a negative binomial distribution with the mean of the observed epochal *R*_t_ and uniformly-distributed dispersion [*k*, 0.1–0.3 (Endo et al. 2020)] and gamma-distributed serial interval with mean = 4.7+/−0.1 days and sd = 2.9+/−0.1 days (Nishiura et al. 2020, Riou and Althaus 2020, Susvitasari et al. 2023). Simulated cases were stochastically diagnosed and sequenced. We drew 100 samples of *n* averted introductions from the simulations without replacement (Figs S27–S33), enforcing the observed proportion of singletons (Table S4). Diagnoses averted were compared to observed daily incidence to evaluate differences in cumulative and maximum daily incidence. Percentage additional cases is the ratio of diagnoses averted to estimated total variant diagnoses in Canada.

To approximate healthcare cost savings, we translated averted COVID-19 diagnoses to hospitalizations, intensive care unit (ICU) visits, and deaths. We assumed a 1% hospitalization rate, similar to 1093.9 hospitalizations per 100 000 cases (1.1%) estimated for the USA between May 2020 and April 2021 (Couture et al. 2022). Although the hospitalization rate is likely overestimated due to incomplete sampling of asymptomatic or undiagnosed cases, we have extrapolated diagnoses averted (not cases averted) to hospitalizations averted, which is valid given the denominator. The average cost per COVID-19 hospitalization (no ICU) in Canada was $15 000 CAD, and the average cost for those admitted to ICU was $55 000 (CIHI 2023). The CIHI estimated 21% of COVID-19 hospitalizations resulted in ICU admission, and 56% of ICU patients received ventilation and 25% died in the facility (CIHI 2023).

## Results

### Epidemiological synopsis of SARS-CoV-2 variants in Canada

This retrospective observational study spans November 2020, when the first variants were identified in Canada, until March 2022, following predomination of Omicron lineages, BA.1 and BA.2 (Fig. 1). Daily and cumulative variant cases were estimated for Canadian provinces as the product of rolling average daily new diagnoses and variant frequency on GISAID.

While the second wave (August 2020–March 2021, Fig. 1) was primarily composed of nonvariant (‘Other’) SARS-CoV-2 lineages, variants became more predominant from fall 2020 onwards. On 9 December 2020, the first COVID-19 vaccine was approved in Canada (Health Canada 2020). By the third wave (March 2021–July 2021), wild-type lineages were outcompeted primarily by Alpha (estimated 307 680 cases in Canada; Table S3; Fig. S4), with notable contributions of Gamma (93 762 cases), Beta (18 326 cases), Eta (19 067 cases), Iota (8325), Epsilon (5591 cases), Zeta (2906), Kappa (2838), and Mu (405) across Canada, and low detection of Delta, first sampled in Canada on 6 March 2021. By May 2021, at least 50% of adults in most provinces and territories had received at least one vaccine dose, though uptake varied (PHAC 2021). The trough between the third and fourth waves in summer 2021 was low enough to justify interventions’ relaxation. The fourth wave (August 2021–December 2021) was driven by Delta, for which we estimated 500 617 Delta cases. Omicron initiated the fifth wave, with 630 408 BA.1 cases, 741 437 BA.1.1 cases, and 38 009 BA.2 cases cumulatively by the end of our study period on 22 March 2022.

### Viral introduction rate reductions from focal sources following travel restrictions

For each VOC, 10 sets of up to 50 000 GISAID sequences consisting of 50% Canadian and 50% global sampling locations were subsampled to reflect the temporal distribution of variant cases over time and regions’ monthly proportional contributions to variant cases (Fig. S9). Ancestral geographies were estimated upon maximum likelihood phylogenies to quantify viral importations, consisting of Canadian sublineages (international importations with sampled descendants) and singletons (importations with no sampled descendants, representing infected travellers or downstream cases with no sampled recent Canadian ancestry), in relation to travel restrictions. Fold reduction of importation rates and proportion of importations from focal regions were calculated 1–4 weeks following implementation of travel restrictions. The ratio of introductions from all sources to cumulative variant diagnoses in the week preceding restrictions and during the early and late periods of each restriction was also estimated.

**Figure 2:**
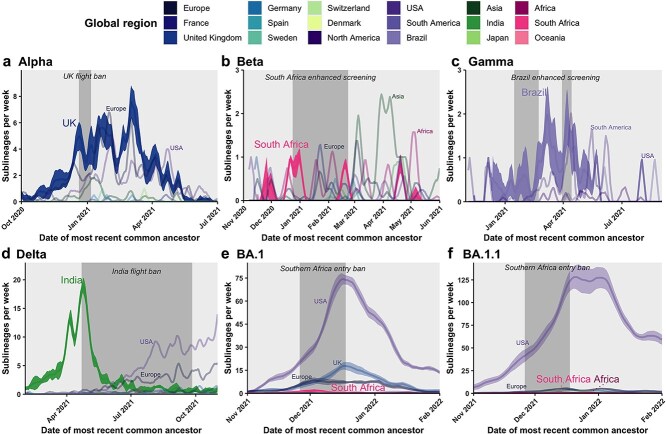
VOC sublineage introduction dynamics into Canada in the context of COVID-19 travel restrictions. The 7-day rolling average weekly sublineage introduction rates were inferred using maximum likelihood phylogeography for (a) Alpha, (b) Beta, (c) Gamma, (d) Delta, and (e) Omicron, BA.1 and (f) BA.1.1. Most introductions of Omicron BA.2 came after travel restrictions and thus BA.2 was excluded. Global regions contributing the most sublineages were directly annotated. Ninety-five percent confidence intervals across 10 subsamples for focal regions of first VOC detection are displayed and additionally for the USA for BA.1 and BA.1.1. Travel restriction durations annotated with shaded grey highlights.

**Figure 3:**
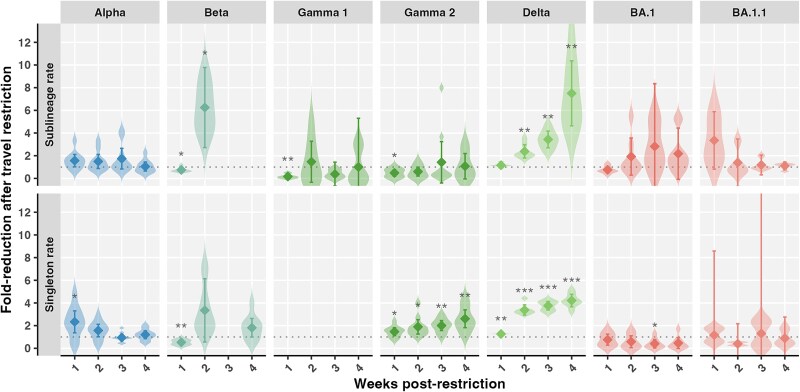
Changes in VOC importation rates from focal sources following travel restrictions. The fold reduction of the 7-day rolling average sublineage and singleton importation rates from focal sources 1–4 weeks after restrictions. Diamonds show the mean, and vertical lines depict the 95% confidence interval calculated across 10 subsamples. Estimates were not shown where comparison included a zero. *P*-values for fold reduction were calculated using one-sample two-sided *t*-tests against a null hypothesis of one (dotted line) and were adjusted for multiple comparisons using the Benjamini–Hochberg method. Significance levels: ^*^ (adjusted *P* = .01–.05), ^**^ (*P* = .001–.01), and ^***^ (*P* < .001).

VOC-specific travel restrictions were variably effective in lowering viral introduction rates from focal regions where variants were first detected. The UK flight ban from 20 December 2020 to 6 January 2021 (intervention duration: 17 days; delay from first Canadian sample collection date in clean GISAID sequences to intervention start: 44 days) in response to Alpha (B.1.1.7) was associated with a 1.57 (95% confidence interval across subsamples, 1.02–2.11)-fold reduction (adjusted *P* = .10) of the sublineage importation rate within 1 week, a 2.33 (1.36–3.30)-fold reduction (*P* = .038) of the singleton rate within1 week, and a 1.15 (0.91–1.39)-fold reduction (*P* = .20) of the proportion of sublineages from the UK (Figs 2a and 3). The importation rate from the UK was held steady amid exponentially rising Alpha cases in the UK (Fig. S19a) and then rebounded following ban repealment, rising to a maximum of 7.6 (6.3–8.8) sublineages per week on 3 March 2021. There were at least 672 (652–693) Alpha introductions into Canada, including 234 (228–240) sublineages and 438 (418–459) singletons (Table S2). The UK contributed 50% (46%–53%) of sublineages prior to the flight ban, 46% (39%–53%) during, and 37% (35%–40%) after (Fig. S45). Altogether, 40% (38%–42%) of sublineages and 45% (43%–48%) of singletons originated in the UK (which represented ~20% of international sequences), followed by Europe, the origin of 31% (29%–32%) of sublineages and 23% (21%–25%) of singletons, and the USA, contributing 23% (22%–24%) of sublineages and 26% (25%–28%) of singletons. In the week preceding the UK flight ban, we estimated Alpha introductions represented 28.8% of diagnoses. During the early period of the restriction, 161.5% of Alpha diagnoses were represented by observed importations, and we predicted Alpha introductions could have represented 163.9% (162.4%–165.5%) of diagnoses. A discrepancy was also found between the observed 7.8% of diagnoses as introductions in the late period, compared to 9.5% (9.2%–9.8%) predicted in the absence of the restriction.

For the Beta variant (B.1.351), implementation of enhanced screening and quarantine for travellers who had been to South Africa, effective 23 December 2020 to 22 February 2021 (duration: 61 days; delay from first sample: 4 days), was associated with a 6.25 (2.72–9.78)-fold reduction (*P* = .049) of the Beta sublineage importation rate from South Africa within 2 weeks, as well as a 1.75 (1.33–2.18)-fold-reduction (*P* = .003) of the proportion of sublineages from South Africa (Figs 2b and 3). This effect appeared delayed, as 1 week following the restriction, the sublineage and singleton importation rates were elevated (*P* = .015; *P* = .0015). During the restriction, there was a rise in Beta sublineages likely to have originated in Europe and other African nations; following restrictions, Beta importations from Asia increased. Overall, of 40 (40–41) total estimated sublineages, 14% (12%–16%) were likely from South Africa, 31% (30%–33%) from Asia, and 20% (18%–22%) from other African nations (Fig. S49). Of 80 (77–83) total singletons, 8% (7%–9%) were likely from South Africa, while 41% (39%–42%) were from Asia, 22% (19%–24%) from Europe, and 18% (17%–20%) from other African nations. Prior to the Beta-related restrictions, introductions represented an estimated 99.1% of Beta diagnoses. In the early period of enhanced screening for Beta, observed introductions represented 11.3% of new diagnoses but may have represented 12.2% (11.7%–12.7%) without the restriction. In the late period, introductions represented ~8.1% of new diagnoses, similar to 7.7% (7.5%–7.9%) predicted.

Neither of the Gamma (P.1)-related restrictions of enhanced screening and quarantine for travellers from Brazil (first intervention, 15 January–22 February 2021, duration: 38 days; delay from first sample: 42 days; second intervention, 30 March–14 April 2021, duration: 15 days) were associated with reductions in sublineage importations from Brazil (Figs 2c and 3). Rather, there was a slight increase in the sublineage importation rate from Brazil 1 week following the first and second interventions (*P* = .0050; *P* = .010). The second period of restrictions was associated with consecutively significant reductions in the singleton importation rates (1 week, *P* = .039; 2 weeks, *P* = .036; 3 weeks, *P* = .0041; 4 weeks, *P* = .0060), as well as a significant 1.60 (1.27–1.93)-fold reduction (*P* = .002) in the proportion of sublineages from Brazil; however, sublineage introductions during this period were sparse (*n* = 3). Brazil was the likely origin for 52% (50%–55%) of the 38 (37–40) Gamma sublineages in Canada, while the USA accounted for 29% (26%–32%), other South American nations for 12% (10%–14%), and other North American nations for 6% (5%–8%) (Fig. S46). Of 159 (148–170) total Gamma singletons, 65% (63%–67%) were likely from Brazil. Most introductions of Zeta (P.2), the sister lineage to P.1, also originated in Brazil (Fig. S47). Prior to the first Gamma-related restriction, there had been negligible Gamma diagnoses in Canada, yet we estimated 4.2 introductions had taken place. In the first period of Gamma restrictions, observed introductions represented 35.9% of new diagnoses, which may have been 37.2% (36.5%–37.9%) without restrictions, suggesting counter-effectiveness. In the second period of Gamma restrictions, observed and predicted ratios were both 0.9%, as cases grew exponentially.

Suspension of flights from India to counter Delta (B.1.617.2) from 22 April 2021 to 26 September 2021 (duration: 157 d; delay from first sample: 47 d) was associated with a 4.0 (1.8–3.0)-fold reduction (*P* = .0060) of the sublineage importation rate from India within 2 weeks and 7.5 (4.6–10.4)-fold (*P* = .0064) within 4 weeks, from a maximum of 19.0 (17.5–20.5) sublineages per week on 24 April (Fig. 2d). The singleton importation rate from India was also reduced 1–4 weeks following the ban (1 week, *P* = .0015; 2–4 weeks, *P* < .001) (Figs 3 and S52). By late June 2021, Delta sublineage importations from the USA and Europe superseded India and, for the USA in particular, rose to a maximum of 20.7 (16.8–24.6) sublineages per week on 4 December 2021. There were at least 1822 (1794–1850) Delta introductions into Canada, including 537 (521–553) sublineages representing 90 (86–93) unique Pango lineages, and 1285 (1253–1317) singletons. The flight ban was additionally associated with a 3.8 (3.6–4.1)-fold reduction in the proportion of sublineages originating from India (Fig. 3). Prior to the restriction, 95% (93%–97%) of 76 (72–80) Delta sublineages were from India, compared to 25% (23%–26%) of 228 (218–237) sublineages during the restriction (Fig. S52). After the flight ban, 233 (225–240) additional sublineages were introduced, primarily from the USA, Europe, and a lesser contribution from India. Overall, the USA was the likely origin for 48% (47%–49%) of Delta sublineages and 50% (49%–52%) of singletons, followed by India for 26% (25%–27%) of sublineages and 21% (20%–22%) singletons, and then Europe for 19% (18%–20%) of sublineages and 20% (19%–21%) of singletons (Fig. S52). India and the USA accounted for 15% and 42% of global Delta sequences sampled, respectively. The flight ban was also associated with a reduction of sister lineage Kappa (B.1.617.1) sublineage and singleton importation rates from India (Fig. S51). In the week preceding the India flight ban, we estimated the ratio of Delta introductions to cumulative new diagnoses as 38.2%. In the early period of the India flight ban, observed introductions represented 4.8% of cumulative new diagnoses, but predicted introductions without the flight ban could have represented 6.1% (5.9%–6.2%) in the early period. In the late period, observed introductions represented only 2.3% of cumulative new diagnoses and were predicted to have been 2.4% without restrictions, signifying lowered ineffectiveness.

The Omicron (BA.1, BA.2)-related entry ban for foreign nationals and enhanced screening for Canadians arriving from southern African nations from 26 November 2021 to 18 December 2021 (duration: 22 d; delay from first sample (retrospectively): 17 d) was largely ineffective towards reducing importations of BA.1 and BA.1.1. No BA.2 introductions resulting in sampled descendants occurred prior to travel restrictions. The flight ban was not associated with a reduction in the BA.1 or BA.1.1 sublineage or singleton importation rates from southern Africa (Figs 2e and f and 3). There was a 2.4 (1.3–3.6)-fold reduction in the proportion of BA.1 sublineages from southern Africa following the ban (*P* = .024): from 3% (1%–5%) of sublineages before to 1% (1%–2%) during restrictions (Fig. S55); however, this coincided with increased importations from the USA, reaching a maximum of 74.2 (71.0–77.4) BA.1 sublineages per week (Fig. 2e) and 128.2 (114.9–141.6) BA.1.1 sublineages per week (Fig. 2f). The USA was the predominant source of Omicron BA.1 and BA.1.1 into Canada up to March 2022, contributing 72% (70%–73%) of BA.1 sublineages (Fig. S55), 90% (89%–91%) of BA.1.1 sublineages (Fig. S56), 74% (73%–75%) of BA.1 singletons, and 92% (91%–93%) of BA.1.1 singletons. Africa and South Africa combined likely contributed 1% (1%–2%) of BA.1 sublineages, <1% of BA.1.1 sublineages, 2% (1%–2%) of BA.1 singletons, and <1% BA.1.1 singletons. Predominant sources of early BA.2 introductions were India and the UK, and negligibly few BA.2 introductions originated from the USA or Africa (Fig. S57). On average the USA was the collection location for 40% of BA.1 and 66% of BA.1.1 of international sequences subsampled. Prior to Omicron travel restrictions, we estimated up to 500 observed introductions for only 10.5 new diagnoses in the week preceding, likely impacted by low testing and ascertainment rates. During the early period of Omicron restrictions, introductions from multiple sources, though mostly the USA, continued to supersede detected diagnoses in observed and predicted scenarios. In the late period, domestic Omicron diagnoses increased exponentially, and observed introductions represented 45.8% of new diagnoses for observed and predicted scenarios.

Introduction rate dynamics for VOIs to which no targeted travel restrictions were applied, including Eta (Fig. S48), Epsilon (Fig. S50), Iota (Fig. S53), and Mu (Fig. S54), displayed only moderate temporal variability.

### Introductions and cases averted *via* variant-of-concern travel restrictions

We quantified introductions averted due to travel restrictions by applying deterministic counterfactual models relating introduction rates from the focal source to estimated variant cases in focal sources, fit to data 6–10 days preceding interventions. This assumes that the relationship between variant cases in the focal source and introductions from the focal source to Canada preceding the restriction would have remained the same had there been no restriction. Sampled and diagnosed cases averted were estimated using stochastic branching processes that reflected empirical epidemic characteristics including the sampling rate, sublineage size distribution, instantaneous reproduction number (*R*_t_) through time across sublineages, and proportion of singletons. These outbreak simulations assume that introductions are independent and that averted introductions mimic observed introductions, in terms of the probability of having further sampled descendants and the total number of sampled descendants, with the underlying assumption of similarly susceptible populations. The reported uncertainty intervals do not capture deviations from these assumptions. Results were summarized as total diagnoses averted, percentage cumulative incidence averted, and percentage maximum daily incidence averted. Analyses were conducted for entire restriction periods, as well as separately for the early and late periods (split roughly in half, and into the two restriction periods for Gamma) to compare their effectiveness. We also estimated averted travellers based on international air arrivals to corroborate phylogeographic findings (Supplementary Materials).

**Figure 4:**
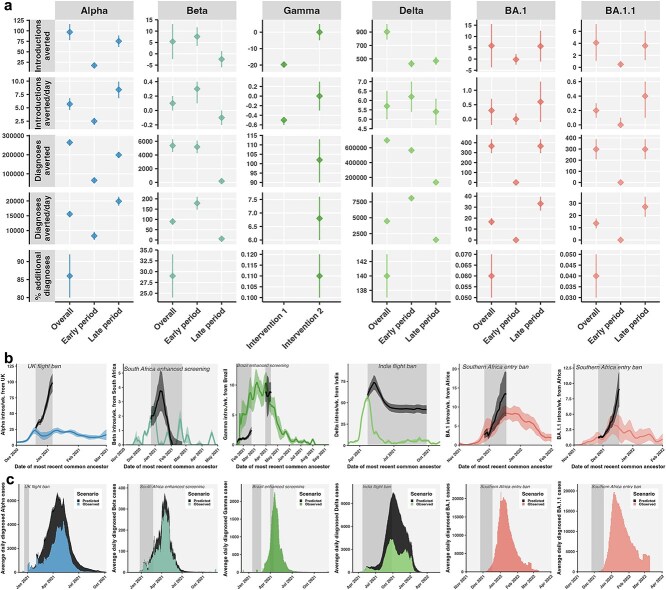
Averted introductions and cases attributable to VOC travel restrictions. (a) Introductions (including sublineages and singletons) and diagnosed cases averted in total and per day. Metrics were estimated for overall intervention and for early and late periods and for separate Gamma interventions. Percentage additional diagnoses averted relative to estimated variant diagnoses in Canada. Diamonds denote the mean, and vertical bars show 95% confidence intervals. (b) Observed variant introduction rates (coloured by variant) and predicted (black) rates in the absence of restrictions from focal source to Canada. (c) Observed daily variant diagnoses in Canada and predicted additional diagnoses in the absence of travel restrictions estimated with stochastic branching processes.

In the absence of the UK flight ban, we estimated there could have been 724 additional travellers from the UK and 97 (78–116) additional Alpha introductions, 77.4% of which we expect would have been singletons. Cumulatively, averted introductions could have resulted in 264 658 (247620–281 697) additional diagnosed cases, corresponding to 21 319 (19 959–22 678) sampled cases (Figs 3a, S19, and  S28). These averted diagnoses represent an additional 86% (80%–92%) of the 324 547 estimated Alpha cases in Canada. Furthermore, the maximum daily incidence at the peak of the Alpha wave could have been 71% (66%–76%) higher and could have occurred 34 (32–36) days earlier. Overall, the UK flight ban averted an average of 15 568 (14 566–16 570) downstream diagnosed cases per day. The ban became more effective over time as daily Alpha cases in the UK rose during the latter part of the flight ban (Fig. S19). In the late period of the Alpha restriction (28 December 2020–6 January 2021), 19 910 (18 403–21 416) diagnoses were likely averted per day, versus in the early period (20–27 December 2020), 8196 (6812–9580) diagnoses were likely averted per day (Fig. 4).

Enhanced screening for travellers from South Africa countering Beta may have averted 5 (−2 to 13) introductions (Fig. 4). The early period of Beta restrictions (24 December 2020–21 January 2021) likely averted more introductions and cases than the late period (22 January–22 February 2021). The early period was associated with 8 (4–12) averted introductions that could have resulted in 5181 (4271–6092) descendant cases, while the late period was associated with −2 (−6 to 1) averted introductions, which if we assume no negative values, could have resulted in 188 (0–384) averted diagnosed cases (Fig. S20). Negative averted introductions suggest an opposite than intended effect, however, with a confidence interval spanning zero. The 5370 (4438–6301) averted cases would have represented an additional 29% (24%–34%) of the cumulative incidence of estimated Beta cases (*n* = 12 462) in Canada (Figs S20 and S29). The average effectiveness for Beta was 90 (74–105) diagnosed cases averted per day, but the early period may have averted up to 179 (147–210) cases per day. Our simulations also predicted that in the absence of restrictions, the peak daily case incidence would have been 15 (10–20) days earlier and 26% (21%–30%) higher (Fig. 4).

Both periods of enhanced screening for travellers from Brazil countering Gamma were ineffective in averting introductions (Figs 4, S21, and  S30). Although the first intervention may have averted 326 travellers, observed introduction rates from Brazil exceeded predicted rates (many were singletons), resulting in −19.7 (−21.4 to 18.1) additional introductions. The second period of enhanced screening, implemented when Gamma cases in Canada were peaking, had a minor to negligible effect, averting 0 (−4.9 to 4.9) introductions. Even assuming the upper limit of 5 averted introductions, we expect that 87.4% of introductions would have been singletons; therefore, 102 (90–113) diagnosed cases, or 6.8 (6.0–7.6) per day, may have been averted, which represents 0.11% (0.10%—0.12%) additional case burden.

The 5-month India flight ban averted many Delta introductions and cases; however, its effectiveness diminished as Delta cases increased globally (Fig. 4). There were 206 Delta introductions observed during restrictions, whereas we predicted that there could have been 1109 (994–1224) without restrictions, amounting to 903 (788–1018) introductions averted or an average of 5.7 (5–6.5) introductions averted per day (Fig. S22). The uncertainty intervals for introductions predicted in the absence of restriction were larger further into the ban. We estimated the ban likely averted 5148 travellers from India, though fewer travellers were averted after June 2021 with rising travel volume *via* other countries (Fig. S18). Averted Delta sublineages could have given rise to 700 577 (68 551–715 641) diagnosed cases (Fig. S31), with the caveat that introduction characteristics were extrapolated for the 5 months restriction duration. Relative to the 491 733 total estimated Delta cases in Canada, this could have represented 140% (137%–143%) additional sampled Delta cases and could have increased the maximum daily incidence by 153% (150%–157%). Overall, 4462 (4366–4558) cases were likely averted per day during the ban; in the early period (22 April–30 June 2021), 8090 (7876–8303) cases were averted per day, which likely decreased to 1526 (1498–1554) in the late period (1 July–26 September 2021), largely owing to the increased proportion of singletons among introductions. Insufficient sequences were available to test the effectiveness of the 2-month Pakistan flight ban on Delta importations; however, we estimated that approximately 116 travellers may have been averted from Pakistan during the ban (Fig. S18f).

For Omicron, the entry ban for foreign nationals and enhanced screening for Canadians who had travelled to southern African nations was ineffective in reducing Omicron burden (Fig. 4). Although the ban may have averted 1681 travellers from Africa, we estimated it averted 6 (−4 to 16) BA.1 and 4 (1–7) BA.1.1 introductions, and 366 (296–435) BA.1 cases and 298 (209–388) BA.1.1 cases, representing average effectiveness of 16.6 (13.5–19.8) BA.1 cases averted per day and 13.6 (9.5–17.6) BA.1.1 cases averted per day (Figs S23, S24, S55, and  S56). Cases averted were negligible compared to Omicron burden, representing 0.06% (0.05%–0.07%) of 634 334 BA.1 cases and 0.04% (0.03%–0.05%) of 779 903 BA.1.1 cases confirmed by 10 March 2022. However, the later Omicron intervention period (8–18 December 2021) was potentially more effective than the early period (26 November–7 December 2021), with 33.2 (26.9–39.5) BA.1 diagnoses averted per day and 27.1 (19–35.3) BA.1.1 diagnoses averted per day, but with negligible relative impact on the Omicron burden. We found no evidence of BA.2 sublineages introduced from South Africa or Africa, and only one singleton introduced in mid-February (Fig. S57).

Variant-specific restrictions were differentially effective but conservatively may have averted at least 995 (841–1151) introductions and 971 371 (321 204–1 004 575) additional diagnosed cases in Canada, if our assumptions hold true regarding averted introductions occurring independently of each other and mirroring observed introductions’ characteristics. The majority of cases averted were Delta (700 577), followed by Alpha (264 658), Beta (5370), Omicron BA.1.1 and BA.1 (664), and Gamma (102). The restrictions that averted the highest percentage of cases relative to the observed variant burden in Canada, in order from highest to lowest, were Delta (140%), Alpha (86%), and Beta (29%), while Gamma and Omicron interventions averted negligibly low percentages of additional cases. Comparing restrictions by diagnoses averted per day, the late period of Alpha restrictions was the most effective (19 910 diagnoses averted per day), followed by the early period of Delta restrictions (8090 diagnoses averted per day), corresponding to periods with exponentially increasing variant cases in focal sources with low domestic prevalence and high susceptibility.

Based on 971 371 (321 204–1 004 575) diagnosed cases averted, we estimated direct healthcare cost savings through averted hospitalizations and deaths. Assuming an average COVID-19 hospitalization rate of 1.1% (Couture et al. 2022), an average cost of $15 000 per COVID-19 hospitalization outside ICU and $55 000 in ICU (CIHI 2023), and that 21% of hospitalized patients were admitted to the ICU (CIHI 2023), of whom 56% received ventilation and 25% died in the facility, we estimate VOC travel restrictions cumulatively may have averted 10 685 (3533–11 050) COVID-19 hospitalizations, 2244 (742–2321) ICU admissions, and 561 (185–580) deaths. The minimum cumulative direct healthcare cost of averted hospitalizations is $250 030 895 ($82 677 910–$258 577 605) Canadian dollars (CAD). This represents a lower bound as we do not consider additional costs incurred from employee absences, postacute sequelae of SARS-CoV-2 infection (i.e., long COVID), or funeral and estate costs.

### Phylogeographic sensitivity analyses

The robustness of inferred viral importation rates to sample size (500–50 000 sequences), geographic grouping, and Bayesian versus ML ancestral reconstruction was evaluated for Alpha, Beta, and Delta. We compared fold reduction of viral importation rates from the focal region associated with travel restriction, as well as proportion of importations from the focal region before and during travel restrictions.

Total inferred introductions overall (Fig. S34) and from the focal source (Fig. S35) scaled relatively linearly with sample size for Alpha and Delta, but not Beta. More introductions and a higher percentage of introductions from the focal source were estimated with more demes (Fig. S35). For Alpha, smaller sample sizes led to lower estimates of percentage of introductions from the focal source than the primary analysis, whereas for Delta, the percentage of introductions from the focal source was robust to sample sizes of at least 5000. Fold reduction in the proportion of introductions from the focal source was relatively robust to sample size and very robust to deme groupings for all variants, especially for subsamples with at least 5000 sequences (Fig. S36). Estimates of introduction rates from focal sources and fold reduction in the introduction rate were sensitive to subsample size and small subsamples (<5000) failed to detect many introductions (Fig. S37). For Alpha and Delta, estimates of fold reduction in the introduction rate for subsamples of at least 5000 were relatively stable.

We also compared relative importation rates inferred using Bayesian discrete trait analysis (DTA) in BEAST v1.10.4 (Minin and Suchard 2008, Lemey et al. 2009, Ayres et al. 2012, Suchard et al. 2018) with one of five trees with 500 sequences used for ML phylogeographic inference (Figs S40–S44). Total introductions, detected as sublineages and singletons in the BEAST analyses, were generally within 20% of estimates from ML inference (Fig. S41). Detection of introductions from focal sources was unstable for low sample size (Fig. S42), but generally, the BEAST estimates fell within the credible range of ML across subsamples. Including more demes was associated with a higher percentage of introductions from the focal source (Fig. S42). Fold reduction of proportions from the focal source was generally higher in BEAST than ML inference; for Delta, estimates were robust to methodology (Fig. S43). Fold reduction of introduction rates from focal was unstable for low sample size (Figs S44 and S37).

## Discussion

Canadian COVID-19 travel restrictions targeting regions where VOCs were first detected were variably effective towards reducing SARS-CoV-2 importations and cases and cumulatively may have averted 995 (841–1151) introductions, 971 371 (321 204–1 004 575) diagnoses, 10 685 (3533–11 050) hospitalizations, and 561 (185–580) deaths. These conclusions were supported by counterfactual models developed to estimate introductions and cases averted, quantifying interventions’ effectiveness and providing insights into which restrictions, and restriction periods, had the greatest impact amid varying epidemiological circumstances. However, these estimates rely on assumptions regarding introductions’ independence to infect populations that are similarly susceptible and that the averted introductions would have had similar characteristics to observed introductions (up to the end of the restriction). Violation of these assumptions is not captured in our uncertainty estimates and may lead to lower than estimated effectiveness if, for instance, introductions during the intervention were more likely to go extinct or not be detected at all than captured in the model.

The suspension of flights from India countering Delta was the most effective travel restriction evaluated according to our estimates, corresponding to a steep decline in the viral importation rate from India, and may have averted over 700 000 additional diagnoses. The ban’s effectiveness likely decreased past June 2021, after which Delta cases and importations from the USA and other countries rose and cases in India decreased, although this finding must be interpreted under the caveat that the relationship between Delta cases in India and introductions from India may have changed over the period for which it was extrapolated. The difference between the observed and predicted ratios of Delta introductions to new diagnoses was larger in the early period before June, compared to late, corroborating that the effectiveness of the flight ban lessened with elevated domestic case burden and rising introduction rates from other sources. An analysis of Delta transmission in Canada found early introductions of AY.25 and AY.27 drove Delta burden in Canada, supporting the futility of later stages of the flight ban (Murall et al. 2021). Repeated extensions of the India flight ban were likely due to concerns surrounding Delta case underreporting and its elevated virulence and transmissibility (Planas et al. 2021, Hart et al. 2022, Jelley et al. 2022). The ban could have been implemented earlier and relaxed earlier in light of evidence that Delta was globally and domestically widespread. By contrast, the entry ban for travellers from southern Africa against Omicron was largely unsuccessful in reducing importations or case burden, as early BA.1 and BA.1.1 sublineages were introduced from other global sources, predominantly the USA. The UK flight ban targeting Alpha increased in effectiveness as cases climaxed in the UK, while both the enhanced screening of travellers from South Africa for Beta and from Brazil for Gamma had lagged effects, became less effective after two weeks, and were leakier. This may reflect the inadequacy of enhanced screening and at-home quarantine measures to drastically reduce introductions and onward transmission.

Many factors can impact travel restrictions’ effectiveness. More importations and cases were averted when restrictions were implemented rapidly following variant detection and emergence. The Delta-related flight bans commenced 47 days after the first Delta case was sampled in Canada and Beta-related measures began 5 days after first detection. Delayed responses were at times exacerbated by delayed detection. For instance, the Delta variant may have been first detected in March 2021, but its origin date was estimated near October 2020 (McCrone et al. 2022; Table S1). Part of the reason the Omicron ban may have been ineffective is that the variant was discovered on 19 November 2021, over a month following the estimated origin of BA.1 around 9 October 2021 (Viana et al. 2022). Delay from emergence to detection could be impacted by countries’ reluctance to be negatively impacted by travel restrictions. By the time travel restrictions were implemented for southern Africa, Omicron had already dispersed globally, and the USA was a dominant early international source of BA.1 beyond to Canada (Tegally et al. 2022).

Restriction effectiveness is also affected by the relative variant prevalence, incidence, fitness, and travel connectivity domestically and in geographies that could act as sources. A restriction targeting an area with a rapidly growing case burden relative to other areas and high travel connectivity is most likely to be effective. Furthermore, outbreak sizes upon introduction can also be reduced in the presence of domestic NPIs, such as restrictions on social gatherings or mask requirements that reduce contact rates or transmission probabilities. For instance, the Delta-related India travel ban from April to September 2021 was implemented amid vaccine passports, social gathering limits, and other NPIs that reduced the probability of introductions leading to large outbreaks. During Omicron travel restrictions, there were fewer domestic restrictions and more fatigue around COVID-19 policies. Legal and logistical travel loopholes could also compromise restriction effectiveness, such as for the India flight ban, where a loophole existed whereby travellers from India and Pakistan could enter Canada indirectly with a negative COVID-19 test upon arrival (Canada Border Services Agency 2021a). Another factor impacting NPI effectiveness is a variant’s fitness imparted by immune evasion, which, in combination with immunological waning from natural infections and vaccines, influences the potential for introductions to become large outbreaks. Variant virulence is also important to consider in terms of potential healthcare burden.

These analyses must be interpreted with acknowledgement of limitations due to lingering biases, multiple sources of uncertainty, and model assumptions. Publicly available genomes and diagnoses over time were affected by differences in case ascertainment across time and geographies, changing due to differences in variants’ virulence, as well as testing capacity, criteria, availability, and reporting. Between jurisdictions, differences in sociodemographic composition further impacted case ascertainment, and differences in resources affected the extent and timeliness of sequence generation and sharing. Sequencing could have been due to hospitalization, association with an outbreak, travel history, or variant confirmation, presenting biases not alleviated through subsampling. Regional sequencing disparities are challenging to resolve and can reduce the accuracy of phylogeographic inferences and undermine estimates of relative importation rates (Liu et al. 2022), particularly if sequencing was sparse in the country of potential origin. Our subsampling strategy reduced bias by lessening the representation of regions that contributed more sequences per case, indirectly increasing the representation of regions with fewer sequences per case; however, differences remained (Figs S12 and S13). With smaller subsamples, the ratio of sequences to cases is more normalized, but this reduces the signal. In our sensitivity analyses, subsamples with fewer than 5000 sequences had variable relative importation contributions across subsamples. Recent methodological developments to include unsampled nodes in phylogeographic inference are promising (Lemey et al. 2020), but not yet scalable to tens of thousands of sequences.

By subsampling sequences probabilistically across multiple subsamples, we considered uncertainty due to geographies’ relative inclusion. Other sources of uncertainty include those imposed by phylogenetic tree inference, whereby trees represent local maxima but not the most likely topology and potential misspecification of substitution models, the relaxed molecular clock model, and the ancestral character evolution model. Phylogeographic reconstruction is sensitive to the number of demes. We evaluated sensitivity of our results to various geographical groupings, with only minor differences in relative focal contributions. Geographical grouping should be based on epidemiological similarity and avoiding demes with an inadequate signal for robust reconstruction (Beerli 2004, Layan et al. 2023).

There is further uncertainty in the estimated relationship and extrapolation of importations to variant cases in the focal source, which could be influenced by other factors, including the domestic NPI stringency, which was highly dynamic in 2021, and could reduce the probability of an introduction seeding onward detected transmission. We modelled counterfactual importation rates during restrictions, but we surmise their impact extended after the restrictions through indirect effects on travel hesitancy or other behavioural changes. Distant extrapolations carry more uncertainty, as was evident in the Delta analysis for a 5-month flight ban. Altogether, averted introductions were likely over-estimated for longer interventions, despite estimating confidence limits that reflect some uncertainty.

While singleton and sublineage introduction rate trends were often closely related, as for Delta, elsewhere they differed. For instance, the second period of Gamma-related restrictions for Brazil reduced the singleton rate 1–4 weeks following the intervention, while there were no reductions in the sublineage rate and even a modest increase in the sublineage introduction rate 1 week following the restriction. The observation that singleton rates could be decreased while sublineage rates were slightly increased or were stable could reflect a coinciding increase in the probability of an introduction becoming a sublineage (due to relaxed domestic stringency) through large social gatherings, low compliance, or ability to quarantine. Separating singletons and sublineages is thus justified by their differential responses to interventions in some contexts.

The ratio of introductions to new diagnoses or incidence can inform the potential immediate effectiveness of travel restrictions (Russell et al. 2021) and represent a lower bound for interventions’ impact on the epidemic, but downstream cases warrant additional consideration. To this end, we also looked at the percentage additional diagnoses averted, largely driven by cases predicted to arise weeks to months following importations based on the characteristics of observed sublineage size and lifespan. Both metrics carry uncertainty in the number of imported cases, which are likely underestimated, and cumulative new diagnoses, which differ from incidence based on delayed diagnosis and variable ascertainment. The relative extent to which each is an underestimate depends on testing criteria and variant severity; the ratio may be overestimated where domestic case ascertainment and severity are low, as for Omicron and where travellers are more likely to be tested. Calculating the ratio within multi-week periods reduces noise related to missed or delayed diagnoses or introductions but is also sensitive to cut-offs defining, e.g., early and later periods of restrictions.

Although epochal changes in sublineages’ *R*_t_ were incorporated into the simulations of cases averted, our methodology did not consider temporal variation of serial intervals, ascertainment rates, and sampling rates. Although we incorporated variability in sampled parameters, estimates of cases averted are sensitive to model misspecification. The distribution of total sampled cases in simulations was representative of the over-dispersed distribution of sublineage sizes observed before the end of the travel restriction, and we enforced the proportion of simulation singletons to reflect the proportion observed.

We cannot discount the possibility that additional cocirculating sublineages would deplete the susceptible population more quickly, and this is a limitation of this approach. While multiple outbreaks could compete for susceptible hosts, the Canadian population is heterogeneous and geographically diffuse and was largely susceptible during 2021. Prior to Omicron, SARS-CoV-2 had not yet swept through the entire Canadian population and vaccine-induced immunity was incomplete, waning, and partially evaded by VOCs such as Delta (Mlcochova et al. 2021). For this reason, we considered introductions as independent seeds, with simulation parameters reflecting observed variant sublineages’ reproduction numbers, maximum size, transmission lifespan, and proportion of singletons, which are all impacted by partial susceptibility. The notion that introductions delay time to peak incidence (Clifford et al. 2020), but do not reduce cumulative cases, is sensitive to the assumption of well-mixed populations, which is an oversimplification for heterogeneously connected and susceptible populations. Our findings support that travel restrictions may, in circumstances with sparse domestic variant cases, low mixing, and infrequent superspreading, reduce burden, not just prolong it.

Flight data, where available, can corroborate or inform estimation of introduction rates. By considering temporal changes in air travel into Canada, we estimated travellers averted to corroborate averted importations from focal sources. Flight data has also been used to model exportation intensities as the product of prevalence and travel volume, in place of inferring sublineages’ origins through ancestral reconstruction (du Plessis et al. 2021). Travel history can also be used to inform priors in travel-aware phylogeographic models (Lemey et al. 2020); however, travel metadata are sparse for public sequences.

Disentangling the effects of public health interventions from viral adaptations remains challenging as VOCs vary in their selective advantages to outcompete co-circulating diversity (Otto et al. 2021). While we have not explicitly considered differential fitness in the context of vaccine and infection-induced immunity, this was indirectly considered by stratifying variants with different reproduction numbers reflecting their fitness advantages (Figgins and Bedford 2021). Longitudinal cohort studies and wastewater surveillance would also be helpful in estimating variant frequencies, positivity rates, period of infectiousness, incidence, and the true sampling rate. Further developments in bridging phylogeographic inference of migration rates and distinct sublineages with mathematical and machine learning models could further improve the accuracy of infectious disease forecasting (Stockdale et al. 2022).

We have provided evidence that travel restrictions can reduce viral introductions and mitigate healthcare burden when implemented rapidly following emergence, with high domestic susceptibility, and limited global detection outside the focal source. Travel restrictions should be considered alongside other NPIs, such as restrictions on large gatherings, improved ventilation, and mask use, when sufficient evidence suggests that there is a high risk to human health; equally, they should be repealed if evidence suggests a variant is widely established or poses a low risk to health. During the height of the COVID-19 pandemic, travel measures became highly politicized and lack of transparency in federal agencies’ decision-making eroded public trust as it was unclear what scientific evidence supported policies (Piper et al. 2022). Future pandemic responses need to be informed by evidence to minimize harm, support compliance, and maximize efficiency.

## Conclusion

Quantifying the effectiveness of public health interventions in reducing pathogen introductions and cases through phylogeography and simulations can contribute to evidence-informed pandemic policies, as we have shown for COVID-19 travel restrictions. Sustained investment in genomic surveillance in wastewater, humans, and animals, as well as ongoing generation and sharing of anonymized viral genomes are necessary for rapid detection, monitoring, and understanding of novel variants and emerging pathogens. Formalizing coordination among neighbouring jurisdictions, particularly those sharing land borders, would increase the probability of successful restrictions.

We conclude by emphasizing that it is critical to consider that travel restrictions can have negative socioeconomic impacts, such as disrupting tourism, international trade, and transportation industries, as well as hindering family visits, education, and medical treatments (Klinger et al. 2021). As such, they may contribute to reluctance from sharing discoveries publicly in countries where variants or emerging pathogens are first detected, diminishing the window of opportunity to prevent a potential new pandemic from being actualized. The scientific evidence presented here that travel restrictions can have public health benefits needs to be viewed in the context of social, economic, legal, and ethical consequences.

## Supplementary Material

Supp_SC2-VOC-travel_VirusEvo_McLaughlin-et-al_accept_20250923_veaf077

Supp_CanCOGeN-consortium-authorship_veaf077

Supp_Table_GISAID-data_veaf077

## Data Availability

Viral genome sequences analysed in this study were sourced from the Global initiative on sharing all influenza data (GISAID) coronavirus (CoV) database and are subject to the GISAID EpiFlu Database Access Agreement. Within this agreement, we cannot distribute data to any third part other than Authorized Users. A full set of all subsampled genome sequence accession IDs is available at GISAID EPI_SET_230510yr (doi:10.55876/gis8.230510 yr), which serves as the data acknowledgement for all originating and submitting laboratories. Those without GISAID access credentials may retrieve information about all data contributors by either clicking on the DOI or pasting the EPI_SET ID in the ‘Data Acknowledgement Locator’ on the GISAID homepage. Full and subsampled alignments can be shared to Authorized Users upon request. Scripts are available at github.com/AngMcL/sc2_canada_variants.
