# Supplementary material for: Phylogeographic evaluation of the effectiveness of Canadian travel restrictions in reducing SARS-CoV-2 variant importations and burden
Source: Virus Evol. 2025 Oct 11;11(1):veaf077. doi: 10.1093/ve/veaf077 (PMC12526992; doi:10.1093/ve/veaf077)
Supplement: Supp_SC2-VOC-travel_VirusEvo_McLaughlin-et-al_accept_20250923_veaf077 [file supp_sc2-voc-travel_virusevo_mclaughlin-et-al_accept_20250923_veaf077.pdf]

Supplementary Materials

Phylogeographic evaluation of the effectiveness of Canadian travel restrictions in reducing SARS-CoV-2 variant importations and burden

Angela McLaughlin[1,2], Vincent Montoya[1], Rachel L. Miller[1], Canadian COVID-19 Genomics Network (CanCOGeN) Consortium, Michael Worobey[3], Jeffrey B. Joy[1,2,4*]

[1] British Columbia Centre for Excellence in HIV/AIDS, Vancouver, BC V6Z 1Y6, Canada
[2] Bioinformatics, University of British Columbia, Vancouver, BC V5T 4S6, Canada
[3] Department of Ecology and Evolution, University of Arizona, Tucson, AZ 85721, United States
[4] Department of Medicine, University of British Columbia, Vancouver, BC V5Z 1M9, Canada

*Corresponding author. British Columbia Centre for Excellence in HIV/AIDS, 608-1081 Burrard Street, Vancouver, BC, Canada V6Z 1Y6. E-mail: jjoy@bccfe.ca

SUPPLEMENTARY METHODS 1
COVID-19 incidence, mortality, and case fatality rates in Canadian provinces and territories 1
Estimating variant cases 5
Subsampling strategy 11
Modeling travelers averted 17
Counterfactual modeling of introductions averted 21
Characterization of observed sublineage sizes and reproduction number, R_T 24
Counterfactual modeling of cases averted with stochastic branching processes 26
Sampling size and deme sensitivity analyses 33
Bayesian phylogeographic sensitivity analyses 41
SUPPLEMENTARY TEXT 46
Synopsis of Canadian COVID-19 travel restrictions during the early variant pandemic 46
SARS-CoV-2 VOC and VOI sublineage and singleton dynamics in Canada 50
REFERENCES 60

Supplementary Methods

COVID-19 incidence, mortality, and case fatality rates in Canadian provinces and territories

Incidence, mortality, and average case fatality rate (CFR) were compared across Canadian provinces and territories to investigate differences in case ascertainment (Figs. S1, S2). Although the predominance of Omicron from January 2022 onwards was associated with lower CFR and testing, affecting the final months of the study period, our analyses assume comparable case ascertainment across provinces and over time.

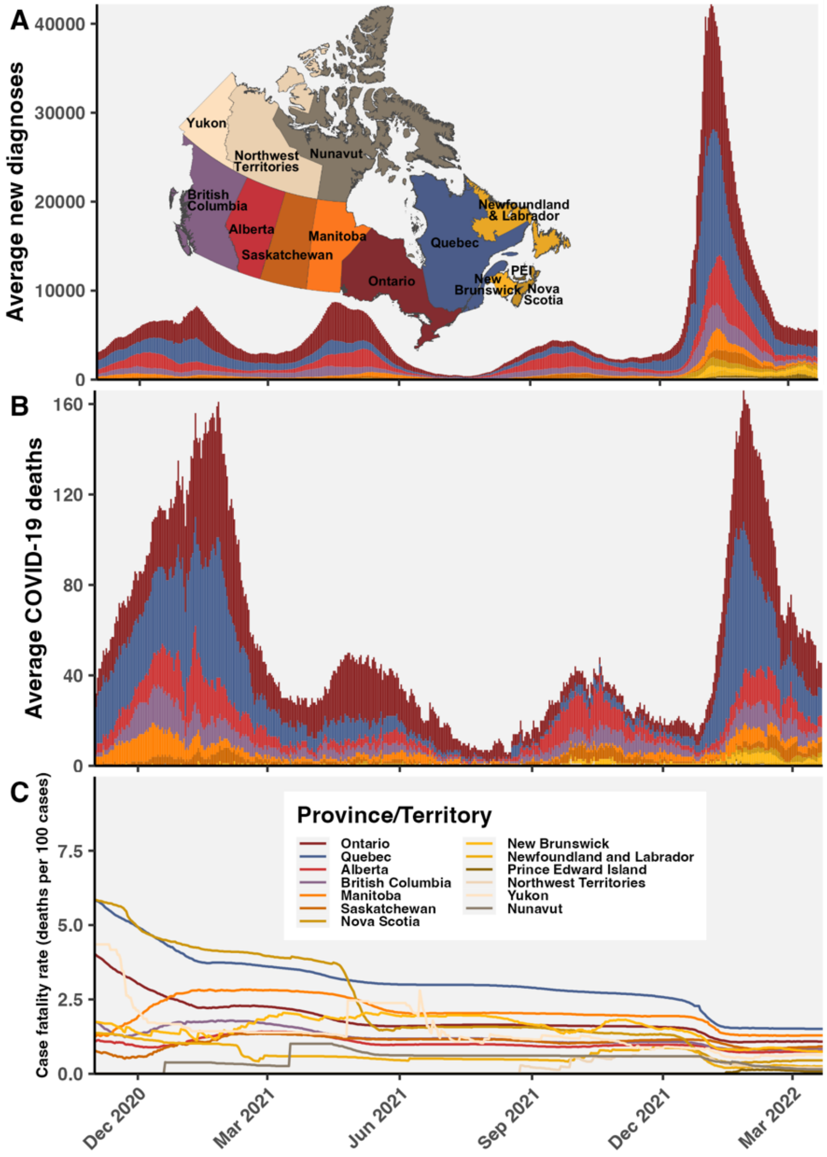

Fig. S1. **Comparison of COVID-19 cases, deaths, and case fatality rate across Canadian provinces and territories.** Average daily **(A)** new COVID-19 diagnoses, **(B)** deaths due to COVID-19, and **(C)** diagnosed case fatality rate from November 1, 2020 to March 22, 2022. Averages calculated as right-aligned 7-day rolling mean. Data from Public Health Agency of Canada (PHAC 2021a).

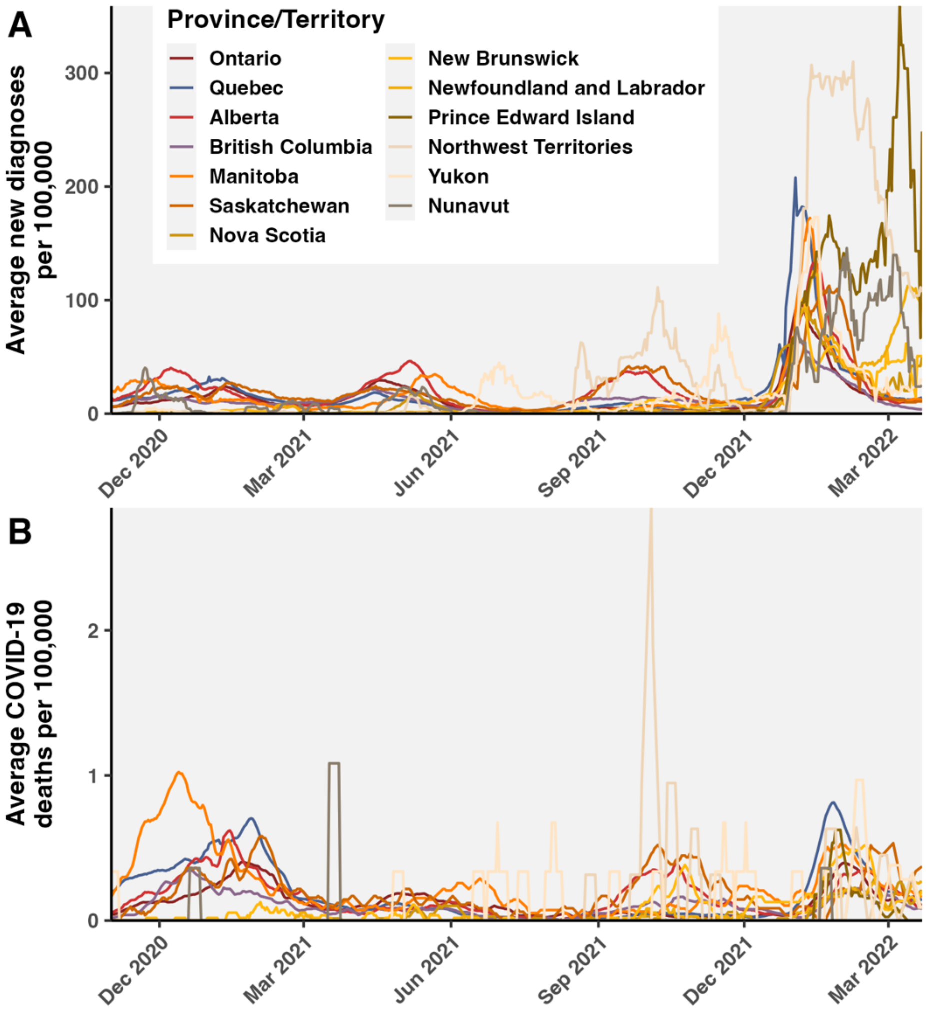

Fig. S2. **Population-normalized daily COVID-19 incidence and death rates in Canadian provinces and territories. (A)** Average daily new diagnoses per 100,000. **(B)** Average daily deaths due to COVID-19 per 100,000. Population sizes for provinces and territories were obtained from the Statistics Canada 2021 Census (Statistics Canada 2022).

Summary of variants of concern and interest

Table S1. SARS-CoV-2 variants of concern (VOCs) and interest (VOIs) up to early Omicron lineages BA.1 and BA.2, ordered by first Canadian sample date. Lambda, Theta, GH/490R, and BA.3 were not included because they were sampled fewer than 100 times in Canada. First global sample dates were obtained from outbreak.info, enabled by data from GISAID, and were updated on 2025-07-01. First Canadian sample dates include clean GISAID sequences, excluding dates preceding first global sample or incomplete dates. Estimates of time of most recent common ancestor (TMRCA) were reported where available.

Variant type	WHO variant	Pango lineage	GISAID clade	First Can. date	First global detection	Outbreak.info earliest date	TMRCA
VOC	Alpha	B.1.1.7; Q.*	GRY	2020-11-06	UK	2020-05-14	2020-08-28 (95 % HPD: 08-15 – 09-09)<br>(Hill et al. 2022)
VOI	Zeta	P.2	GR/484K.V2	2020-11-24	Brazil	2020-04-15	n/a
VOC	Gamma	P.1	GR/501Y.V3	2020-12-04	Brazil	2020-09-11	2020-11-15 (HPD: 10-06 – 11-24)<br>(Faria et al. 2021)
VOI	Epsilon	B.1.427;<br>B.1.429	GH/452R.V1	2020-12-05	California, USA	2020-08-21 (B.1.427)<br>2020-07-06 (B.1.429)	n/a
VOI	Eta	B.1.525	G/484K.V3	2020-12-14	Nigeria; UK	2020-12-11	n/a
VOC	Beta	B.1.351	GH/501Y.V2	2020-12-19	South Africa	2020-07-09	2020-08 (HPD: 07-15 - 08-30)<br>(Tegally et al. 2021)
VOI	Kappa	B.1.617.1	G/452R.V3	2021-02-26	India	2020-09-15	n/a
VOC	Delta	B.1.617.2; AY.*	GK	2021-03-06	India	2020-10-15	2020-10-19 (HPD: 09-06 - 11-29)<br>(McCrone et al. 2022)
VOI	Iota	B.1.526	GH/253G.V1	2021-03-17	New York, USA	2020-11-15	n/a
VOI	Mu	B.1.621	GH	2021-06-08	Colombia	2020-09-19	n/a
VOC	Omicron	BA.1; BA.2	GRA	2021-11-04	South Africa; Botswana	2021-01-27 (BA.1)<br>2021-03-25 (BA.2)<br>2021-11-18 (BA.3)<br>2022-01-06 (BA.4)<br>2021-12-09 (BA.5)	BA.1: 2021-10-09 (HPD: 09-30 – 10-20)<br>(Viana et al. 2022)<br>BA.2: 2021-11 (HPD: 10-09 – 11-29)<br>(Tegally et al. 2022)<br>BA.4/.5: 2021-11 (09-29 – 12-06)<br>(Tegally et al. 2022)

Table S2. Summary of inferred introductions of SARS-CoV-2 variants by March 22, 2022. Introductions consist of sublineages (international introductions Canada resulting in sampled onward transmission) and singletons (introductions with no sampled onward transmission). Mean and 95% confidence interval were calculated using t-distribution across ten subsamples.

WHO variant	Pango lineage	N introduction	N sublineages	N singletons	% singletons of introductions
Alpha	B.1.1.7; Q.*	672 (652-693)	234 (228-240)	438 (418-459)	65.2 (64.1-66.2)
Zeta	P.2	24 (24-24)	9 (9-9)	15 (15-15)	62.5 (62.5-62.5)
Gamma	P.1	197 (185-210)	38 (37-40)	159 (148-170)	80.7 (80.0-81.0)
Epsilon	B.1.427; B.1.429	76 (74-78)	27 (26-27)	49 (48-51)	65 (63.8-66.1)
Eta	B.1.525	62 (59-65)	24 (23-24)	38 (36-41)	61.6 (60.4-62.7)
Beta	B.1.351	120 (117-124)	40 (40-41)	80 (77-83)	66.7 (65.8-66.9)
Kappa	B.1.617.1	102 (101-103)	21 (20-21)	81 (81-82)	79.8 (79.3-80.3)
Delta	B.1.617.2; AY.*	1822 (1794-1850)	537 (521-553)	1285 (1253-1317)	70.5 (69.8-71.2)
Iota	B.1.526	50 (49-52)	14 (13-14)	37 (36-38)	72.6 (71.3-73.9)
Mu	B.1.621	62 (61-62)	7 (7-7)	55 (54-55)	88.5 (88.1-89.0)
Omicron	BA.1	3634 (3524-3744)	615 (596-633)	3019 (2920-3119)	83.1 (82.6-83.5)
Omicron	BA.1.1	5572 (5337-5806)	1148 (1103-1194)	4423 (4223-4624)	79.4 (78.8-79.9)
Omicron	BA.2	549 (537-565)	87 (83-92)	462 (451-473)	84 (83-85)

Estimating variant cases

We estimated variant cases by multiplying daily rolling average GISAID variant frequencies by rolling average confirmed diagnoses for all of Canada and globally (Fig. S3), Canadian provinces (Fig. S4), and global regions (Fig. S6). Monthly proportional contributions of geographic areas to variant cases informed the subsampling probabilities of sequences from Canadian provinces (Fig. S5) and global regions (Fig. S7).

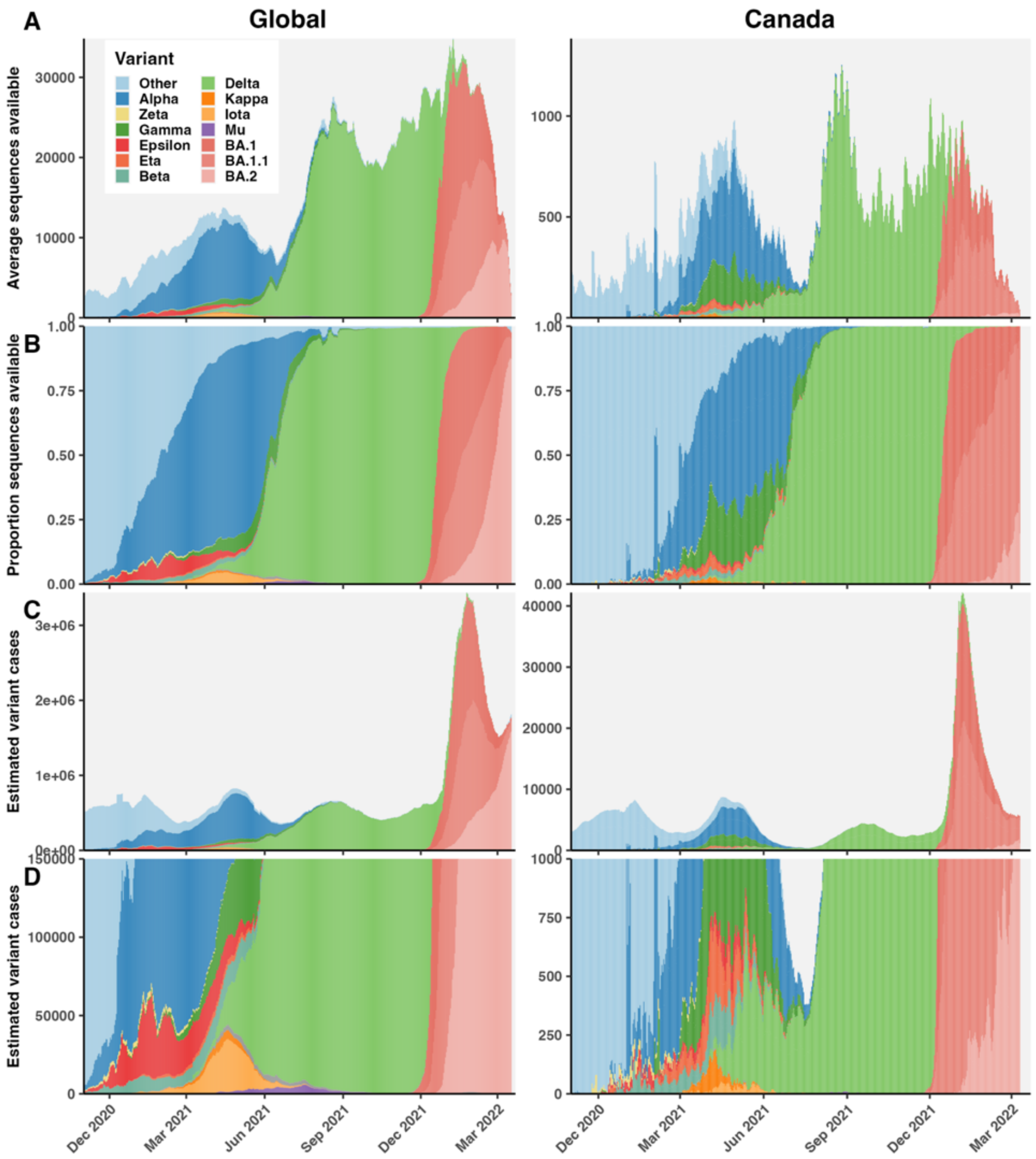

Fig. S3. Estimation of Canadian and global variant cases as the product of diagnoses and variant frequencies. (A) Average daily SARS-CoV-2 genome sequences available on GISAID after cleaning, by variant. **(B)** Proportional frequencies of variants among sequences available. **(C)** Estimated variant cases, and **(D)** zoomed in below 1000 cases/day and 150,000 cases/day.

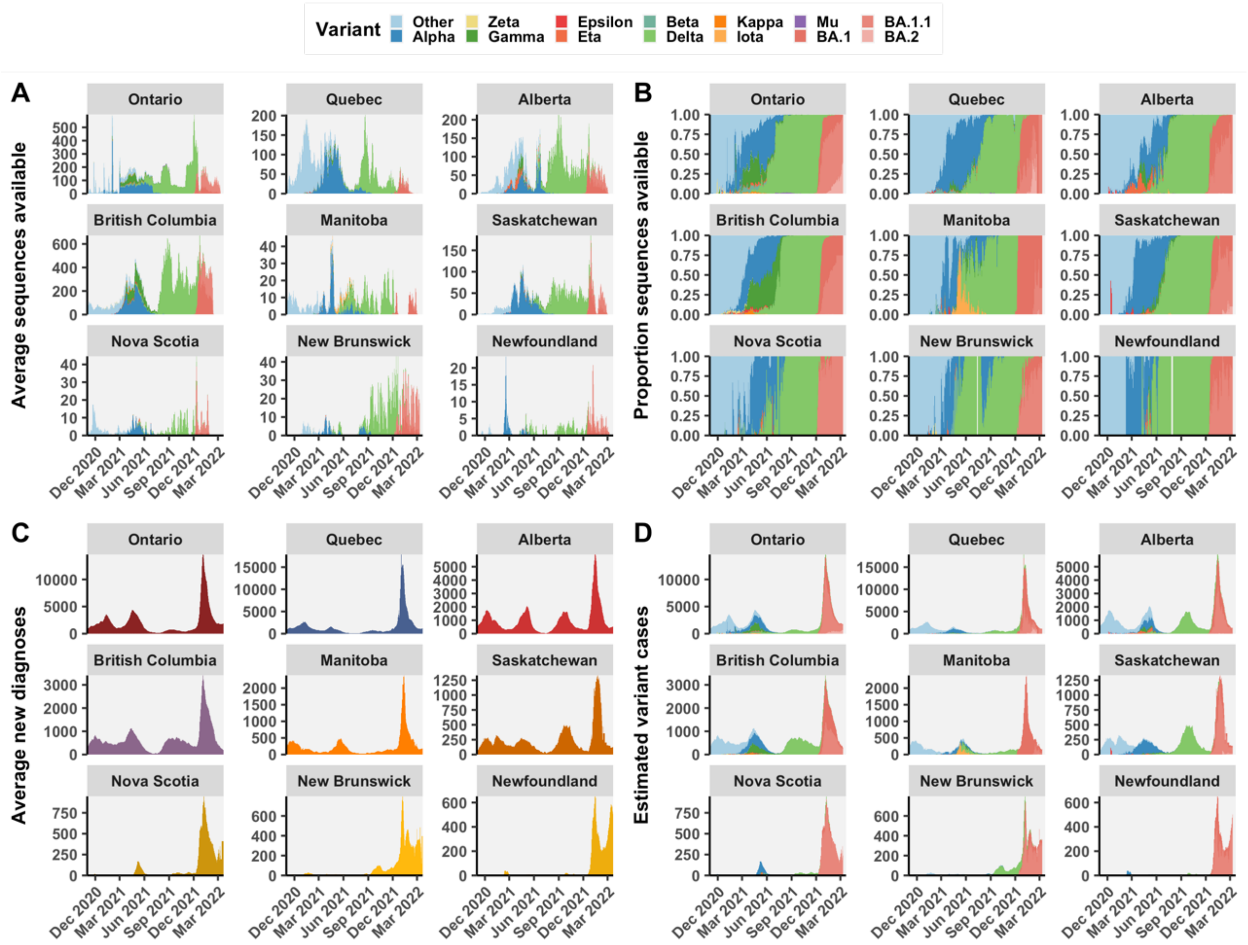

Fig. S4. Estimation of daily variant cases by Canadian province. (A) 7-day rolling average number of clean GISAID sequences available per day by province, colored by variant. **(B)** Daily average proportion of variant sequences within each province. If data was incomplete in the past 7 days, the raw count was used. For days with no sequences but with non-zero cases, variant proportions from the previous day with sequences available were carried forward. **(C)** Daily average new diagnoses by province**. (D)** Daily estimated variant cases by province. Only provinces with sequences available on GISAID were shown.

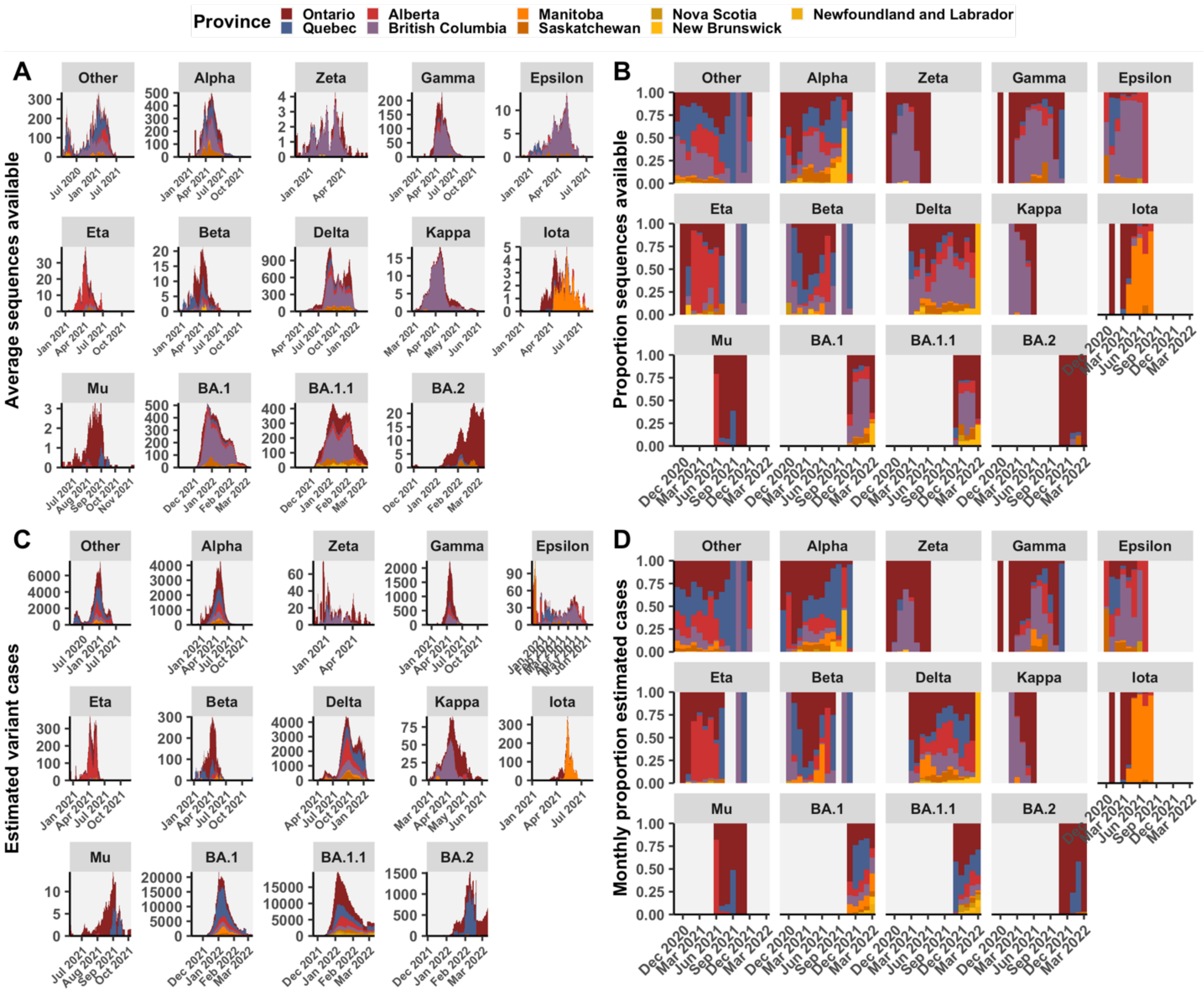

Fig. S5. Distribution of available sequences and estimated variant cases by Canadian province. (A) The rolling 7-day average daily and **(B)** monthly proportional sequences available by variant colored by province. **(C)** The estimated daily variant cases and **(D)** monthly proportional contribution of each province to variant cases; the latter informs the sampling probability of sequences in the analysis.

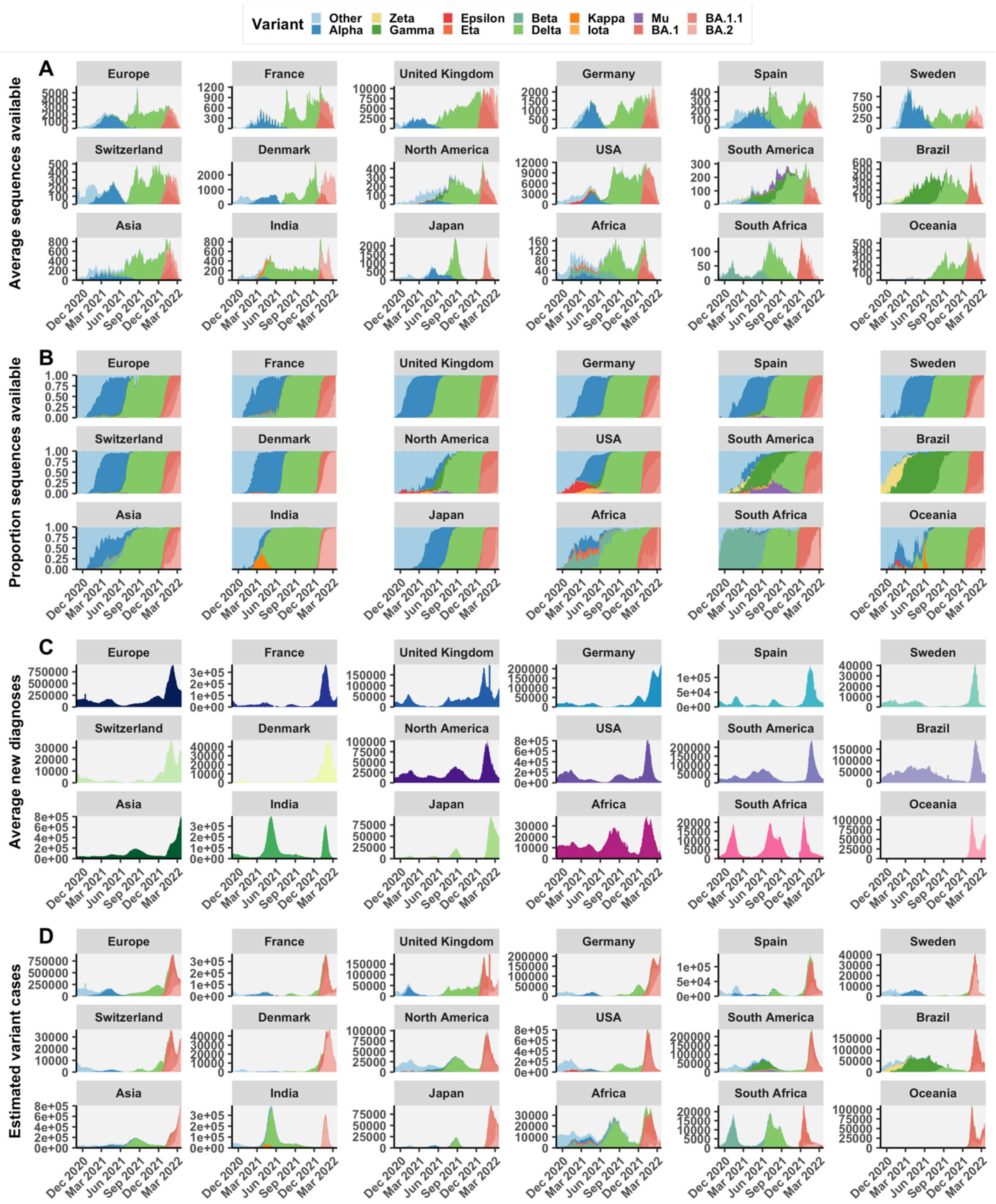

Fig. S6. Estimation of daily variant cases by global region. (A) Daily average clean GISAID sequences available by region, colored by variant. **(B)** Daily average proportion of sequences contributed by each variant to regional totals. If data was incomplete in the past 7 days, the raw count was used. For days with zero sequences and non-zero cases, variant proportions from the previous day with sequences available were carried forward. **(C)** Daily average new diagnoses by global region. **(D)** Daily estimated variant cases by region.

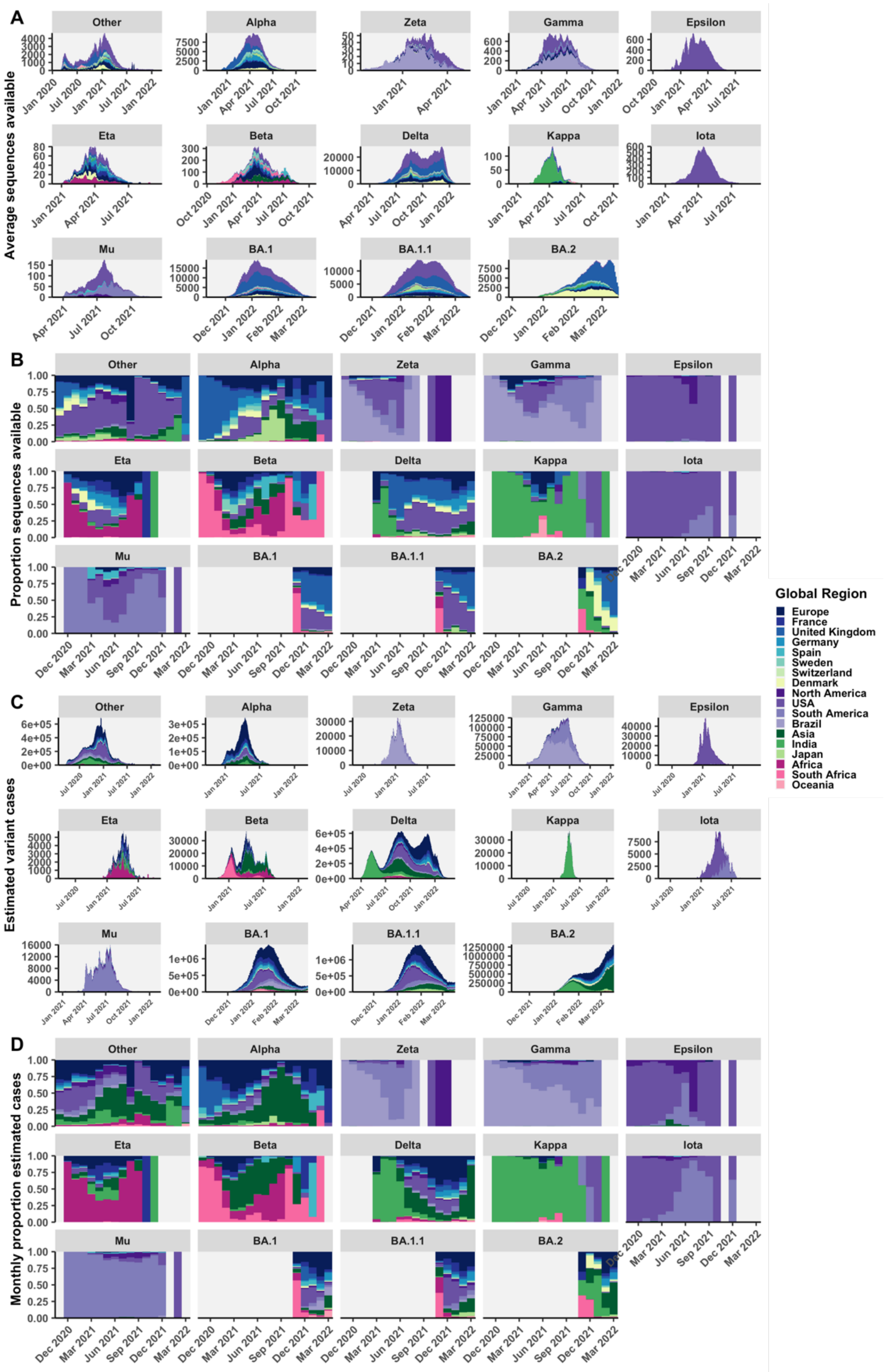

Fig. S7. Distribution of available sequences and estimated variant cases, by global region. (A) The rolling 7-day average daily and **(B)** monthly proportional sequences available by variant colored by region. **(C)** The estimated daily variant cases and **(D)** monthly proportional contribution of each region to variant cases; the latter informs the sampling probability of sequences in the analysis.

Table S3. Cumulative estimated SARS-CoV-2 variant cases globally and in Canada up to March 22, 2022, relative to clean sequences available on GISAID. Cumulative cases by variant were summed across global regions or Canadian provinces. Excludes sequences with unidentified lineage, Theta, Lambda, and GH/490R.

	Global			Canada		
**Variant**	**Cumulative cases**	**Clean sequences**	**Sequence per case**	**Cumulative cases**	**Clean sequences**	**Sequence per case**
Other	109,632,102	942,487	0.009	930,569	58,001	0.062
Alpha	31,471,326	1,065,890	0.034	307,680	42,596	0.138
Zeta	2,520,842	4,721	0.002	2,906	244	0.084
Gamma	15,750,416	98,939	0.006	93,762	13,488	0.144
Epsilon	3,223,780	63,369	0.02	5,591	752	0.135
Eta	445,556	6,733	0.015	19,067	1,800	0.094
Beta	4,290,486	35,606	0.008	18,326	1,414	0.077
Delta	111,501,622	3,921,060	0.035	500,617	111,192	0.222
Kappa	1,638,294	6,621	0.004	2,838	481	0.169
Iota	917,808	38,216	0.042	8,325	302	0.036
Mu	1,369,309	13,140	0.01	405	126	0.311
BA.1	65,751,679	903,272	0.014	630,408	21,242	0.034
BA.1.1	69,901,000	791,147	0.011	741,437	21,920	0.03
BA.2	42,513,492	404,349	0.01	38,009	817	0.021

Subsampling strategy

In our previous study of the first two waves of SARS-CoV-2 in Canada in 2020 and early 2021, we subsampled sequences from provinces or countries with probabilities reflecting geographies' monthly proportional contributions to cases (McLaughlin et al. 2022). The more data were subsampled, the less bias due to overrepresented geographies; however, when below 50% of data were Canadian, the less likely we were to identify introductions. A split of approximately 50% each of global and Canadian sequences (which was 75% of all Canadian sequences available) per subsample identified the most introductions. The current analysis subsampling strategy differs from our previous analysis firstly because there were magnitudes more sequences available for this period of the pandemic (~60,000 Canadian and ~500,000 global sequences; vs. ~300,000 Canadian and >5M global sequences); secondly, variants are not distinguished in the case data, which necessitated estimating variant new cases over time (described above). Geographies' monthly contributions to variant cases informed sequences' sampling probabilities, downweighing geographies with relatively more sequences per cases. As an additional difference to our analysis of the first two waves, we applied temporally distributed sampling of sequences per month that mimicked the distribution of variant cases (Fig. S5, S7), whereas previously we attempted to sampled uniformly in each month. We augmented sampling of sparse early months (Fig. S8), where cases may be relatively low, but we want sequences to be representative of early dynamics.

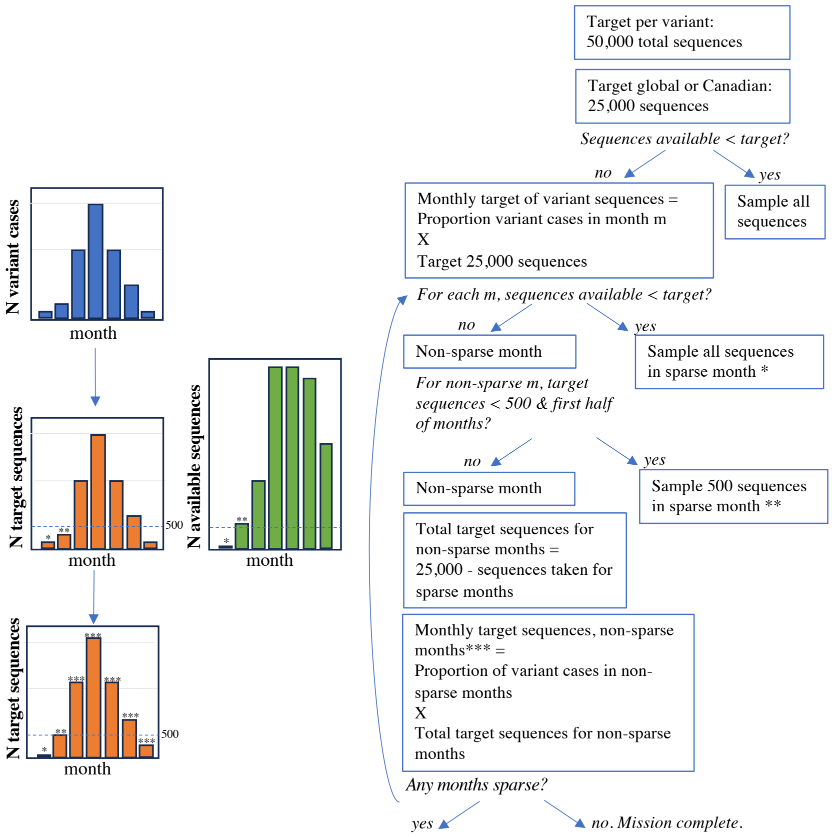

Fig. S8. Graphical depiction of algorithm for temporally-distributed subsampling with augmented sampling of sparse early months. Sequences subsampled mimic the distribution of variant cases over time with number of target sequences topped up to a minimum for early months with fewer cases.

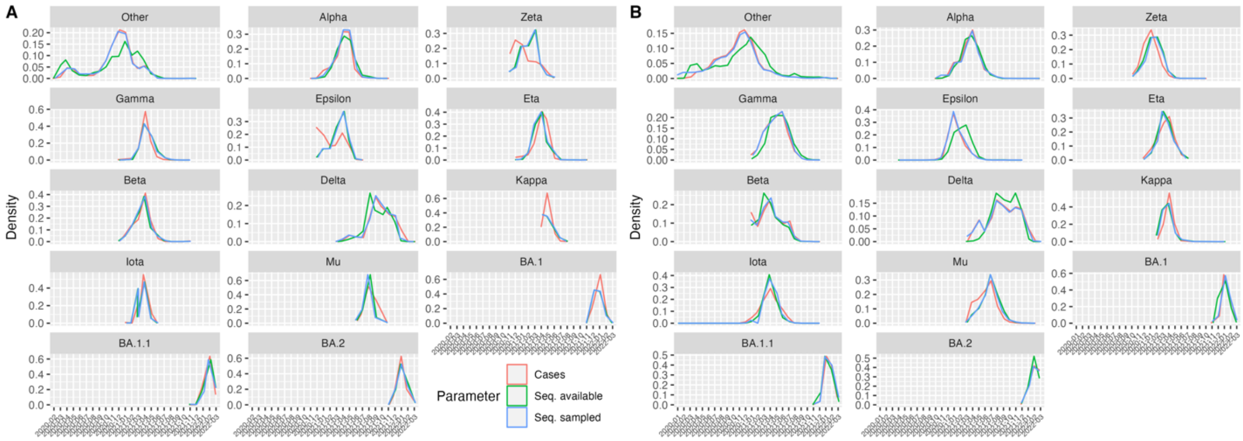

Fig. S9. Relative monthly distributions of cases and sequences in (A) Canadian and (B) global datasets. Proportions of cases, available sequences ('seq. available'), and sequences taken ('seq. sampled') using the temporally-distributed sampling strategy for each variant in a representative subsample.

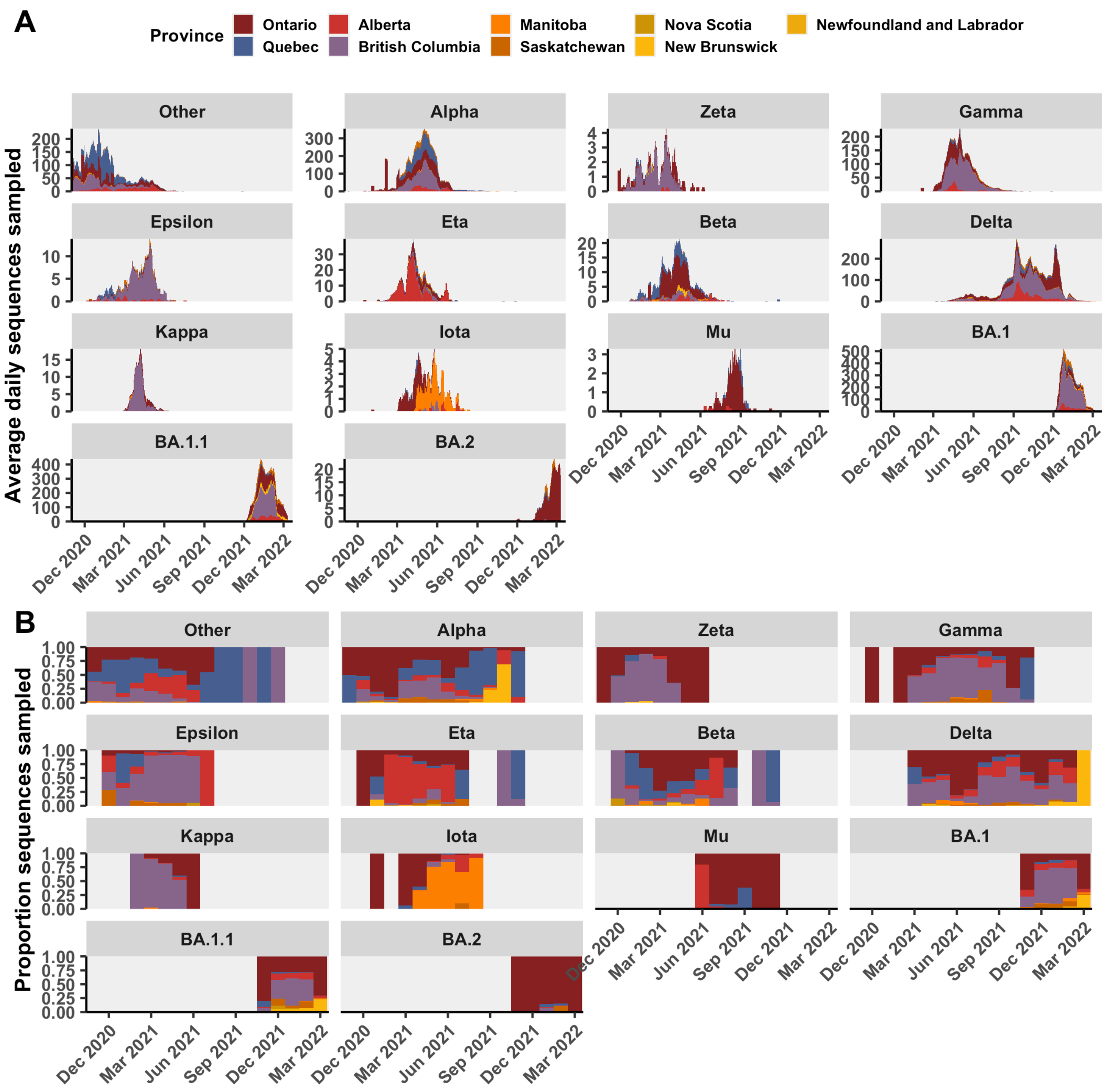

Fig. S10. Average daily and monthly proportion of sequences sampled for each Canadian province for a representative subsample, grouped by variant.

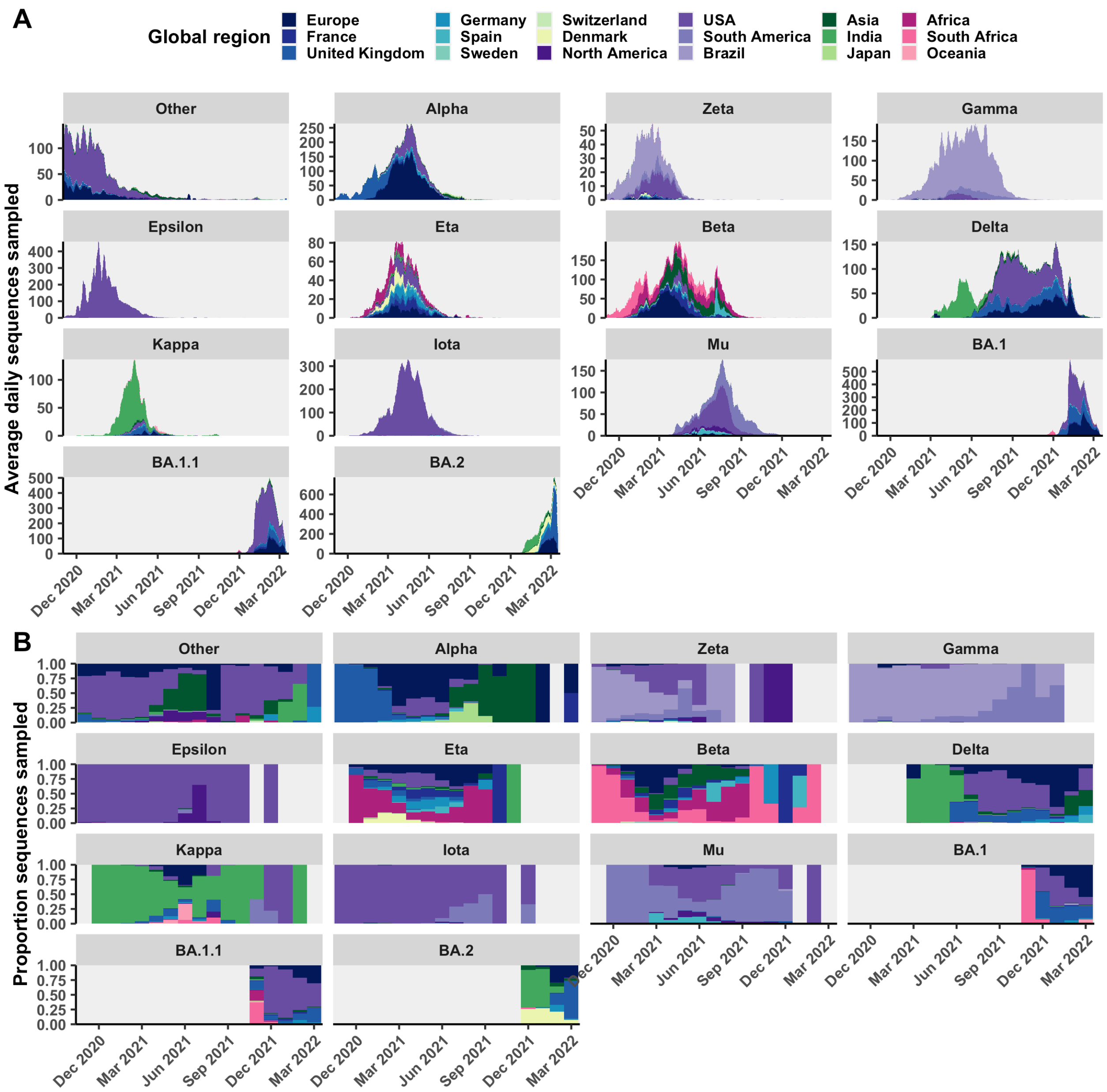

Fig. S11. Average daily (A) and monthly proportion (B) of sequences sampled for each global region for a representative subsample, grouped by variant.

Subsampling improved correlations of monthly cases to sequences among Canadian provinces and global regions (Fig. S12, S13). For variants like Delta, where sequences were abundant but differentially collected and submitted (widely different sequence per case ratio), subsampling improved the correlation for Canadian provinces (Pearson's correlation coefficients: from 0.58 to 0.69) and global regions (from 0.47 to 0.76). Similarly, Alpha correlation improved for global (0.60 to 0.90) and Canadian (0.65 to 0.77) following subsampling. The correlation between monthly sequences sampled and monthly cases was improved more with temporally distributed sampling than uniform sampling for Alpha and Delta variants.

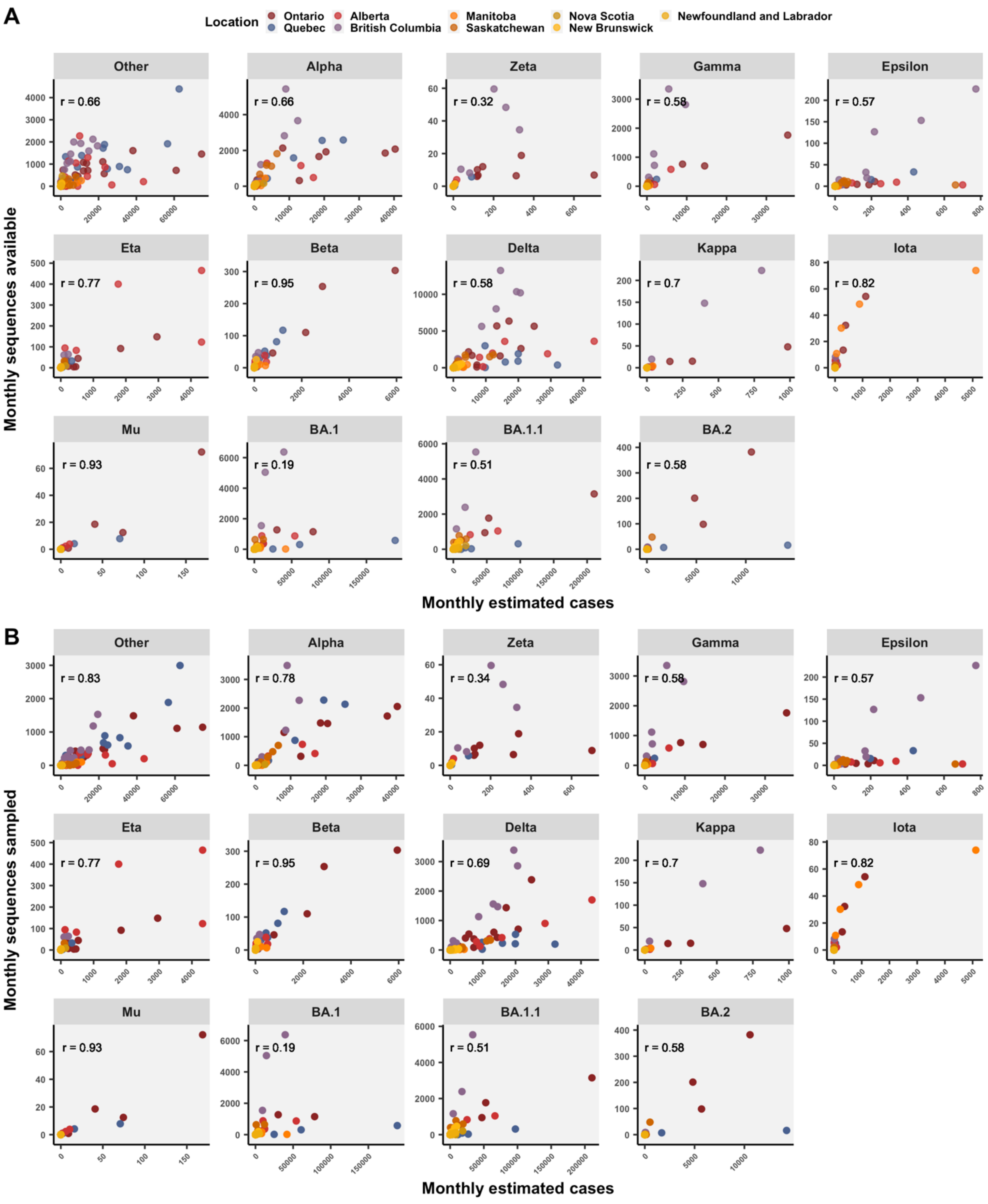

Fig. S12. Canadian provinces' sequence representation per case before and after subsampling in the primary analysis (target sample size 50,000). The relationship between Canadian monthly estimated variant cases and **(A)** monthly sequences available (pre-subsample) for provinces, or **(B)** monthly sequences sampled. Relationships summarized by Pearson's correlation coefficients.

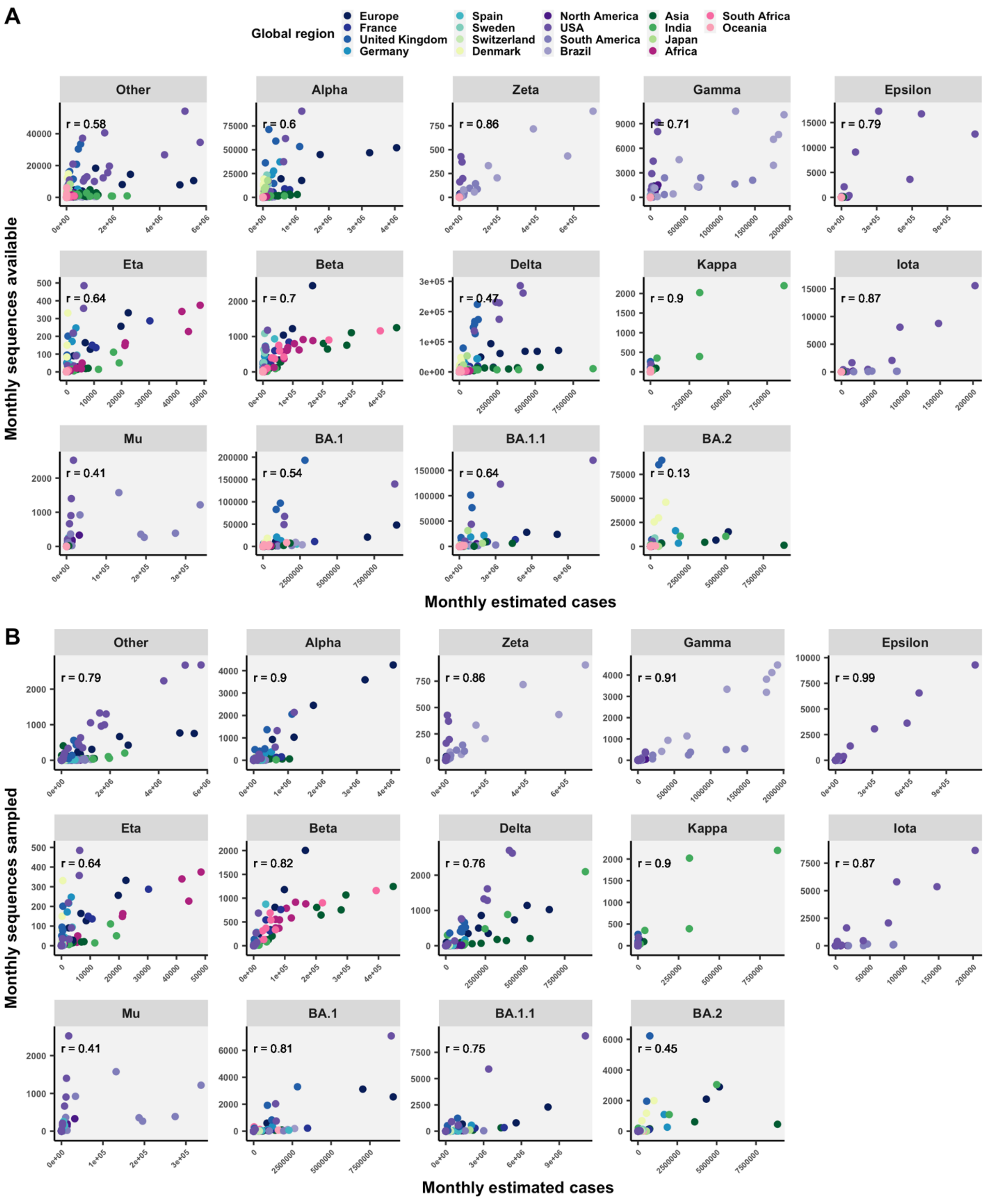

Fig. S13. **Global regions' sequence representation per case before and after subsampling.** The correlation between global monthly estimated variant cases and **(A)** monthly sequences available (pre-subsample) for global regions, or **(B)** monthly sequences sampled. Correlations summarized by Pearson's correlation coefficients.

Modeling travelers averted

Statistics Canada data on international travel arrivals was used to estimate likely travel volume in the absence of travel restrictions for VOCs (Statistics Canada 2023). We describe the methods for Delta as an example, for which we analyzed travel volume from India and Pakistan to Canada from 2018 to 2023**.** Average daily travelers by month revealed seasonality (Fig. S14C), with higher-than-average travel volume from India to Canada in April and May, and to a lesser extent June to August, in 2018 and 2019 (pre-COVID-19), and 2022 (low to negligible COVID-19 restrictions). Seasonality was quantified as the deviation of monthly compared to annual average daily travelers - either as the difference in the monthly and annual means ('diff.means') or the ratio of monthly to annual means ('ratio.means') (Fig. S15). Across 2018, 2019 and 2022, we summarized the mean monthly deviation from the annual trend (Fig. S15C). Expected travel in the absence of the travel ban considering seasonality was estimated as the annual mean travel volume, adjusted by adding the monthly diff.means ('expected.diffmeans') or by multiplying by ratio.means ('expected.ratiomeans') (Fig. S15D).

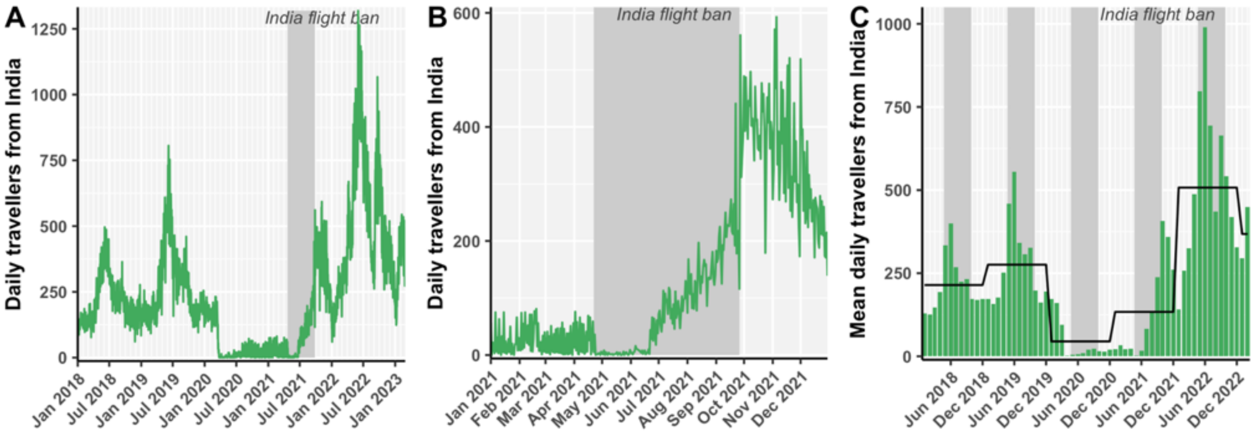

Fig. S14. **Statistics Canada data on international arrivals from India 2018 – 2023.** Observed daily travelers from India to Canada **(A)** from January 2018 to 2023, **(B)** in 2021. **(C)** Monthly mean daily travelers from India; black line is mean daily travelers in calendar year. Time of flight ban (April 22 - Sept 26) highlighted in adjacent years.

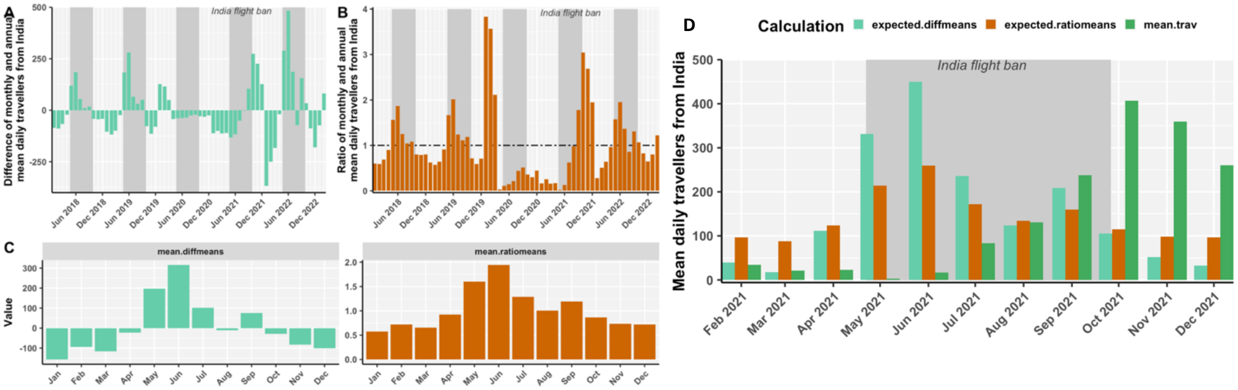

Fig. S15. **Seasonal variation in travel volume from India**. **(A)** Difference between monthly and annual mean daily travelers ('diffmeans'). **(B)** Ratio between monthly and annual mean daily travelers ('ratiomeans'). **(C)** The mean monthly deviation from the annual trends in 2018, 2019, and 2022. **(D)** Observed ('mean.trav') and expected mean daily travelers from India in the absence of the COVID-19-related flight ban based on seasonality trends.

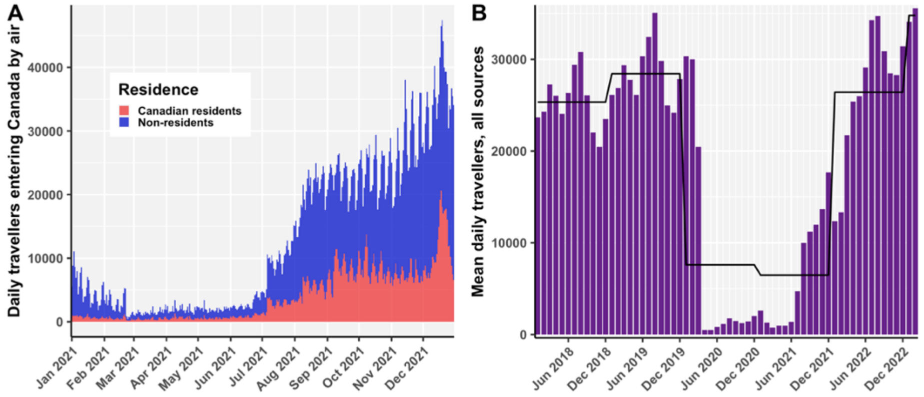

Fig. S16. Observed daily air travel volume into Canada from all sources. **(A)** Daily travelers entering Canada by air, colored by Canadian residents versus non-residents in 2021. **(B)** Monthly mean daily travelers from 2018 to 2022 from all sources, with black line for annual means.

We next considered epochal changes in travel volume from all sources into Canada to incorporate broad changes in travel patterns due to other COVID-19 restrictions (Fig. S16). Non-resident visitors and Canadian-resident visitors daily travel volume was steadily low in 2021 until July 5th, when the requirement for fully vaccinated air travelers eligible to enter Canada to quarantine for three days in a hotel and provide a test after eight days (PHAC 2021a). In September 2021, fully vaccinated foreign nationals were allowed to enter Canada for non-essential reasons, corresponding to a further increase in travel volume. To quantify an epochal effect in 2021, we calculated the difference and ratio between expected daily travelers (resident and non-resident, all sources) based on seasonal trends in 2018, 2019, and 2022, and the observed daily travelers in 2021 from all sources (Fig. S17). The monthly ratio of means is an adjustment factor that takes into consideration broad travel patterns affected by other policies and willingness to travel into Canada in 2021. Monthly adjustment factors were used to correct our expectations of monthly

travel in the absence of flight bans in India (Fig. S17D). This adjusted expectation considers monthly seasonality in travel from India to Canada based on historical data, average travel from India to Canada in 2021, and epochal trends in air travel from all sources in 2021. We converted this to a daily expectation by fitting a spline to the monthly averages. The number of travelers averted was calculated by comparing expected (no ban) to observed (with ban) travelers. This process was repeated for UK (Alpha), Brazil (Gamma), South Africa (Beta), and Africa (Omicron).

During the Delta-related India flight ban, we estimated 5,147 travelers from India were averted via the restrictions (17,958 expected during the ban, and 12,811 observed; Fig. S18). We estimated 724 travelers from the UK were averted during the Alpha intervention; only 3 travelers from South Africa were averted during the Beta intervention; 383 travelers were averted from Brazil over both Gamma-related restriction periods; 1681 travelers from Africa were averted during the Omicron-related travel restriction. In addition to these estimates being useful to corroborate estimations of importations averted, we evaluated adjusting for expected travel volume as a confounder in the model between importation rates and variant cases in focal source.

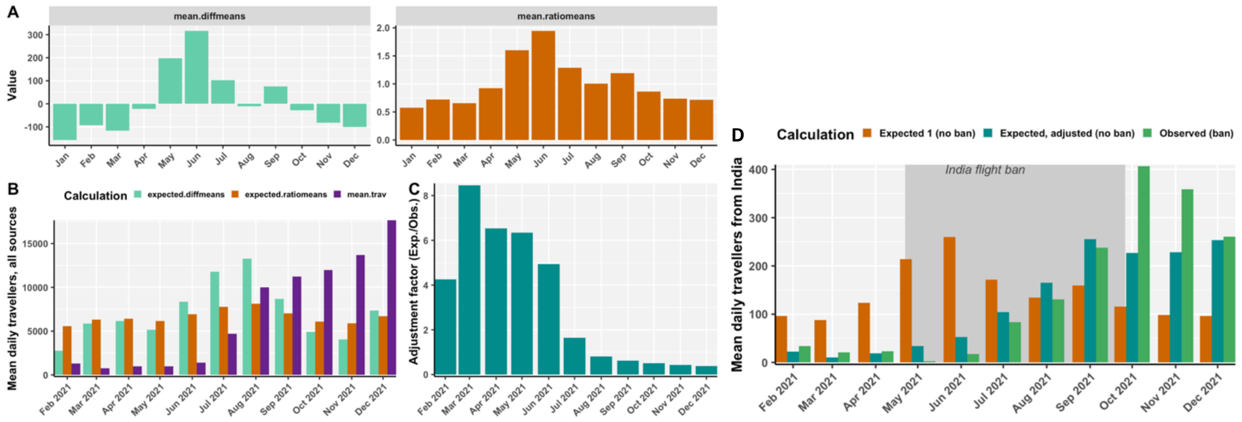

Fig. S17. **Seasonal variation in travel volume from all sources**. **(A)** Mean monthly deviation from annual trend, summarized as the monthly mean across 2018, 2019, and 2022 of diffmeans or ratiomeans. **(B)** Expected mean daily travelers from all sources versus observed ('mean.trav'), using diffmeans or ratiomeans. **(C)** 2021 monthly adjustment factor reflecting epochal changes in overall travel based on ratio of expected (based on 'ratiomeans') and observed mean daily travelers. **(D)** Expectations of monthly travel volume from India in 2021: 'Expected 1 (no ban)' based on seasonality and 'Expected, adjusted (no ban)' based on seasonality and epochal travel.

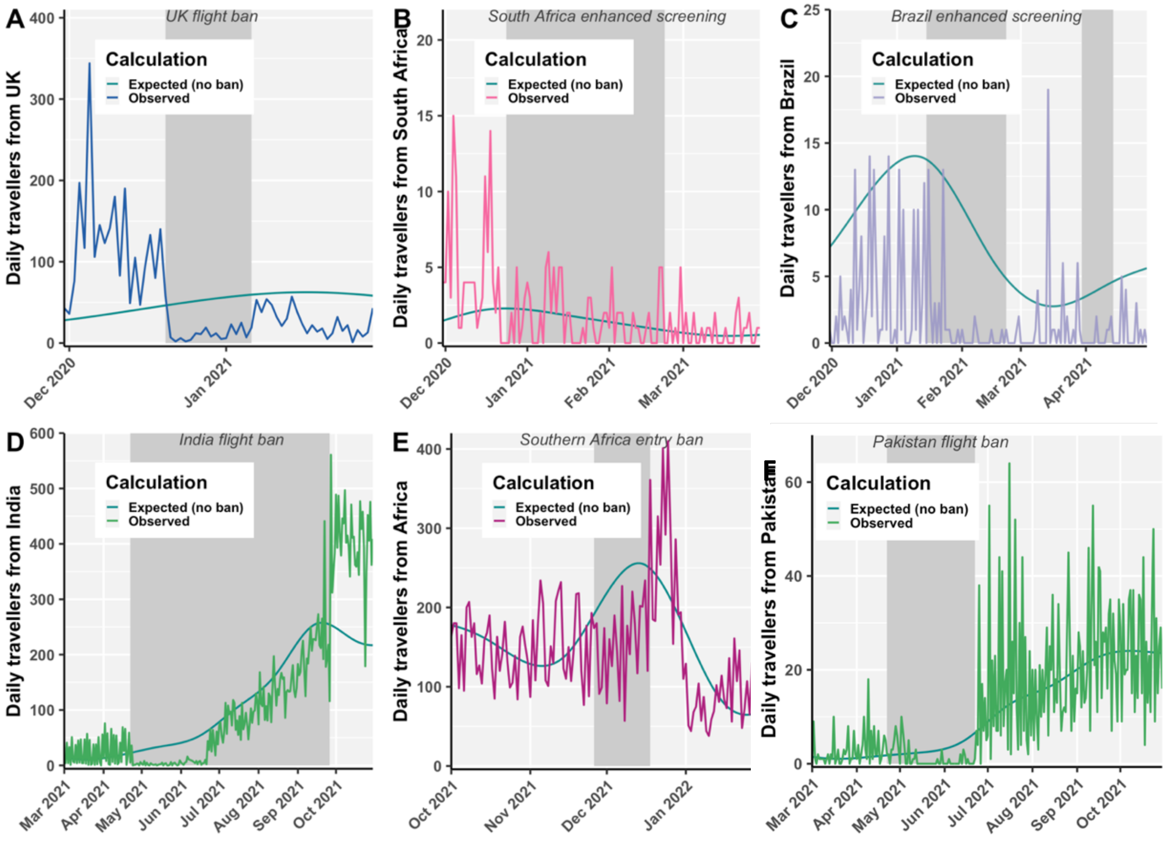

Fig. S18. Expected versus observed daily travelers from focal countries affected by COVID-19 travel restrictions, for **(A)** UK (Alpha), **(B)** South Africa (Beta)**, (C)** Brazil (Gamma), **(D)** India (Delta), and **(E)** Africa (representing southern Africa; Omicron), and **(F)** Pakistan (two-month flight ban countering Delta).

Counterfactual modeling of introductions averted

Observed daily variant cases in the focal source during the intervention and expected travel volume were used to deterministically predict introductions per week in the absence of restrictions with linear regression models, which had better goodness-of-fit than Poisson and negative binomial. Models were trained on data from the week preceding travel restriction, or where variant cases in the source region exceeded a threshold chosen to maximize the correlation between cases and importations (Figs. S19-25). We compared nested models additionally adjusting for travel volume using likelihood ratio tests. In most cases travel data did not improve the fit. Averted introductions were calculated as the difference in the area under the curve (introductions per day) observed and predicted.

For Alpha, the introduction model was fit to data up to 9 days preceding restrictions, corresponding to greater than 5000 daily mean cases in the UK (Fig. S19, adjusted R-squared=0.959). For Beta, the introduction model was fit to data up to 11 days preceding restrictions, corresponding to greater than 6000 daily mean cases in South Africa (Fig. S20, adj. R-squared=0.668). For Gamma, the model for the first intervention was fit to data up to 25 days preceding restrictions corresponding to greater than 5000 daily mean cases in Brazil (Fig. S21, adj R-squared=0.684). The second Gamma intervention was modelled to data up to 10 days preceding the intervention, with 73050 - 77120 daily Gamma cases in Brazil (Fig. S21, adj. R-squared=0.02), but the model was not improved by adjusting for travel volume (LRT, p=0.664). For Delta, the introduction model was fit to data up to 10 days preceding restrictions corresponding to greater than 70,000 daily mean cases in India (Fig. S22). For Omicron BA.1, the model was fit to data up to 6 days preceding restrictions corresponding to greater than 400 daily mean cases in South Africa and other African nations grouped together (Fig. S23, adj. R-squared=0.859). For Omicron BA.1.1, the model was fit to data up to 6 days preceding restrictions corresponding to greater than 30 daily mean cases in Africa (Fig. S24, adj. R-squared=0.925), and although adjusting for travel volume improved the fit, the fit was deemed sufficient. Including travel volume observed as a covariate did not improve the goodness-of-fit based on likelihood ratio test of nested models for any other variants.

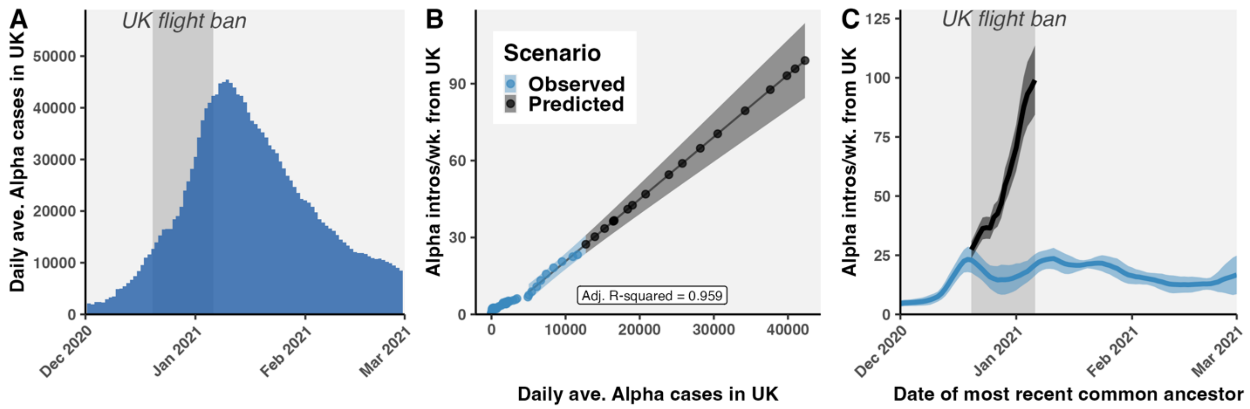

Fig. S19. **Estimation of Alpha introductions averted. (A)** Estimated average daily Alpha cases in the UK. **(B)** Linear model of Alpha introductions (sublineages and singletons) per week from the UK versus average daily Alpha cases in the UK was used to predict introduction rates in the absence of restriction. **(C)** Observed and predicted Alpha introductions from the UK per week.

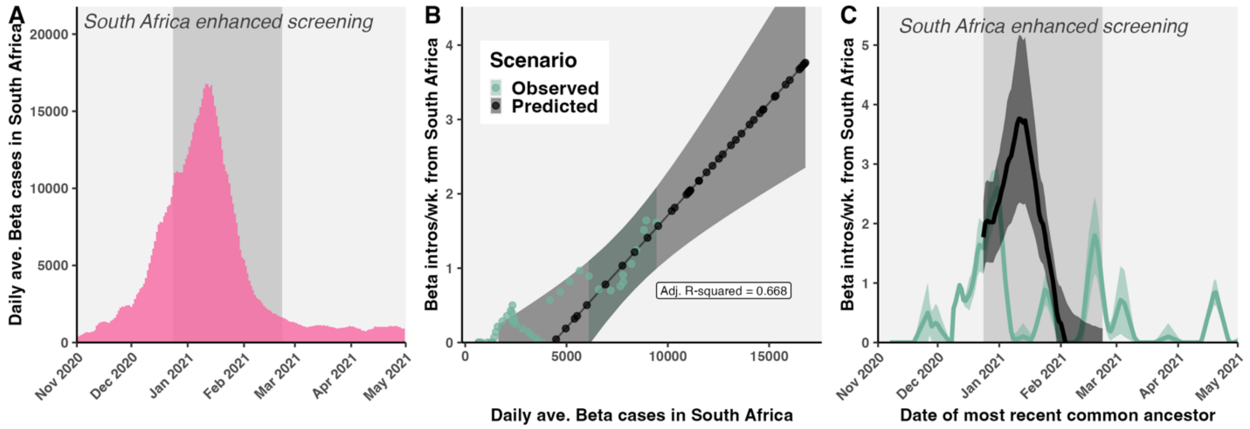

Fig. S20. **Estimation of Beta introductions averted. (A)** Estimated average daily Beta cases in South Africa. **(B)** Linear model of Beta introductions per week from South Africa versus average daily Beta cases in South Africa was used to predict introduction rates in the absence of restriction. **(C)** Observed and predicted introductions per week.

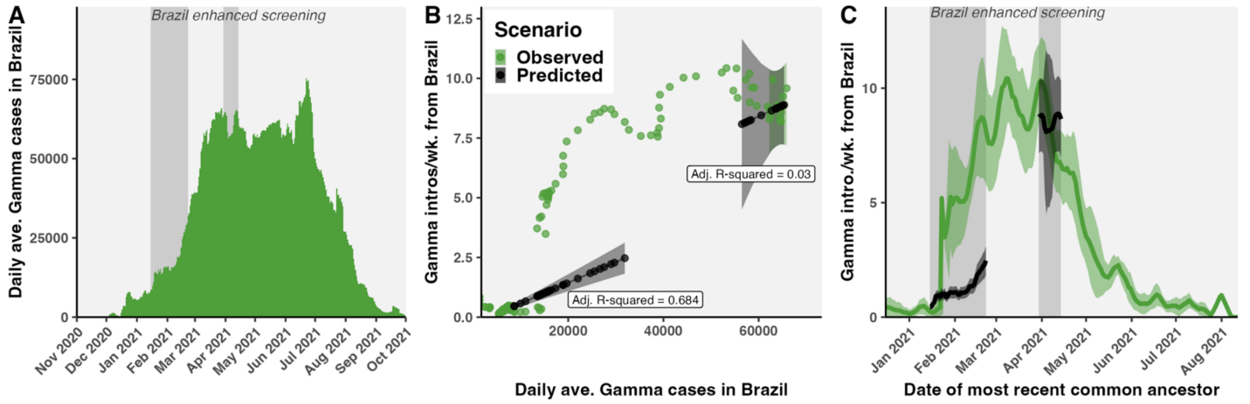

Fig. S21. **Estimation of Gamma introductions averted. (A)** Estimated average daily Gamma cases in Brazil. **(B)** Linear models of Gamma introductions per week from Brazil versus average daily Gamma cases in Brazil prior to the two interventions were used to predict introduction rates in the absence of restriction. **(C)** Observed and predicted introductions per week during two interventions.

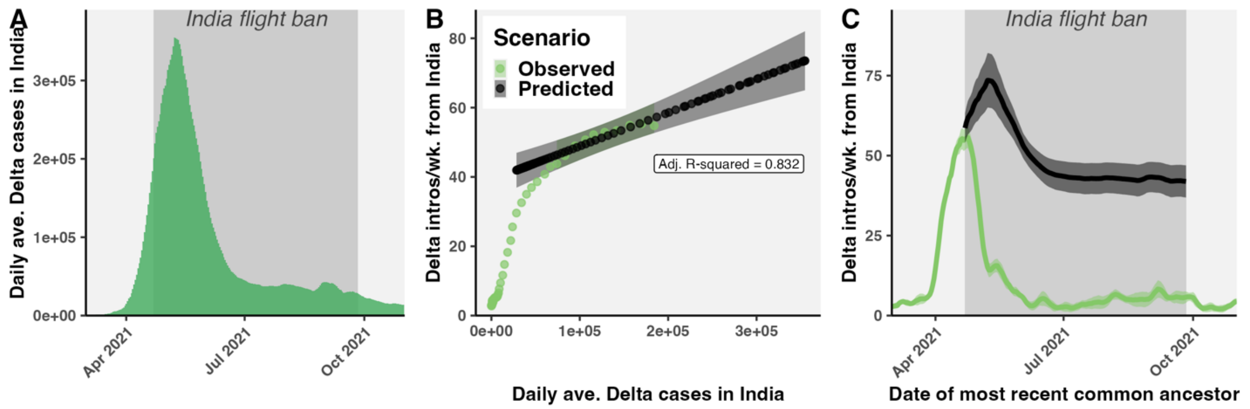

Fig. S22. Estimation of Delta introductions averted. (A) Estimated average daily Delta cases in India. **(B)** Linear model of Delta introductions per week from India versus average daily Delta cases in India was used to predict introduction rates in the absence of restriction. **(C)** Observed and predicted introductions/wk.

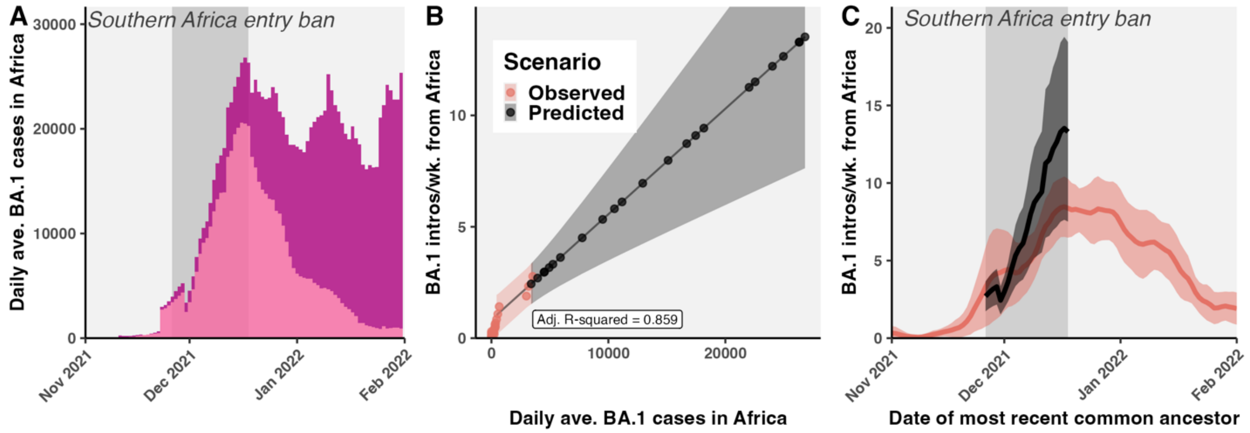

Fig. S23. **Estimation of Omicron BA.1 introductions averted. (A)** Estimated average daily Omicron BA.1 cases in Africa (dark pink) and South Africa (light pink). **(B)** Linear model of BA.1 introductions per week from all of Africa versus average daily BA.1 cases in Africa was used to predict introduction rates in the absence of restriction. **(C)** Observed and predicted introductions per week.

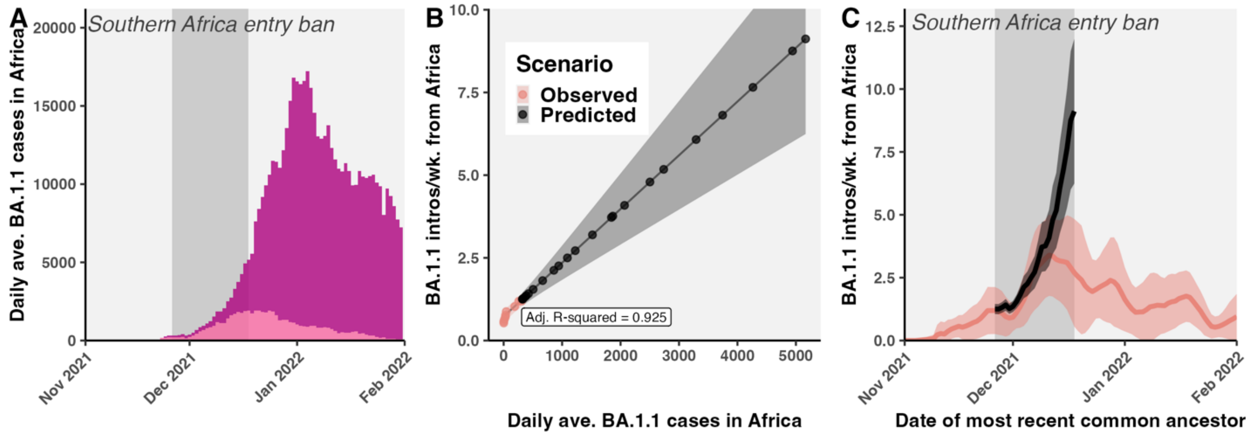

Fig. S24. Estimation of Omicron BA.1.1 introductions averted. (A) Estimated average daily Omicron BA.1.1 cases in Africa (dark pink) and South Africa (light pink). **(B)** Linear model of BA.1.1 introductions per week from all of Africa versus average daily BA.1.1 cases in Africa were used to predict introduction rates in the absence of restriction. **(C)** Observed and predicted introductions per week.

Characterization of observed sublineage sizes and reproduction number, R_t

To simulate outbreaks as accurately as possible, we characterized observed sublineages' transmission, quantified by instantaneous R_t, as well as the distribution of observed sublineage sizes, lifespan (time from first to last Canadian sample), and proportion of introductions detected as singletons (with a single sample) (Table S4). Sublineages' R_t were estimated using EpiEstim with a gamma-distributed serial interval with mean=4.7 days and sd=2.9 days (Nishiura et al. 2020; Riou and Althaus 2020) and 14-day estimating window (Fig. S25). The epochal median R_t across sublineages was calculated daily, excluding observations with CI width greater than 10, then was fit with a smoothing loess curve (Fig. S25). As epochal R_t estimates were unstable at later stages of respective sub-epidemics, while observed cases were in decline, epochal R_t was truncated to 0.8 or 0.9 following last observed sub-epidemic peak (Fig. S26).

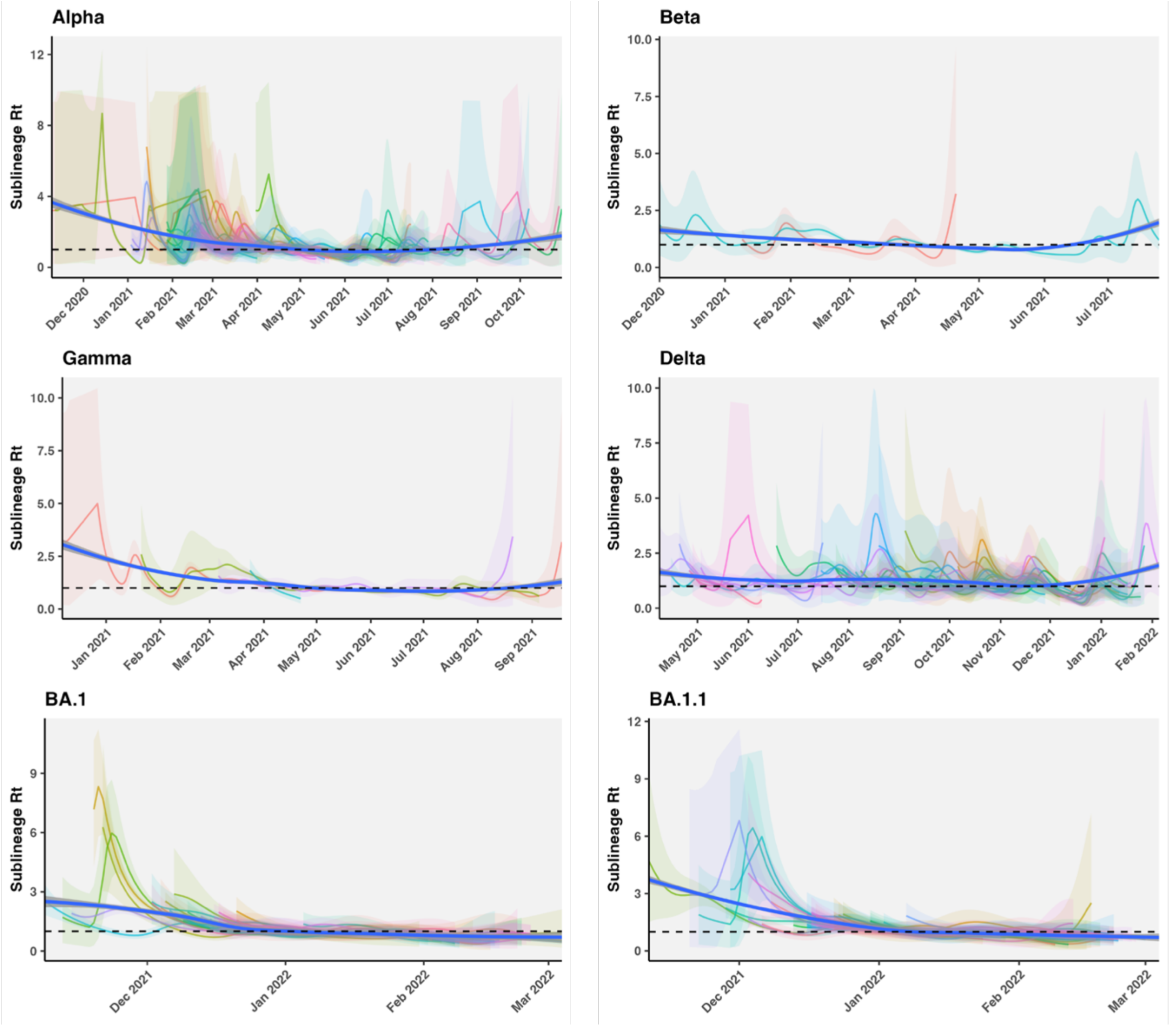

Fig. S25. Overlaid trajectories of sublineage-level R_t for observed variant sublineages with at least fifty sampled descendants. Each color represents a unique sublineage.

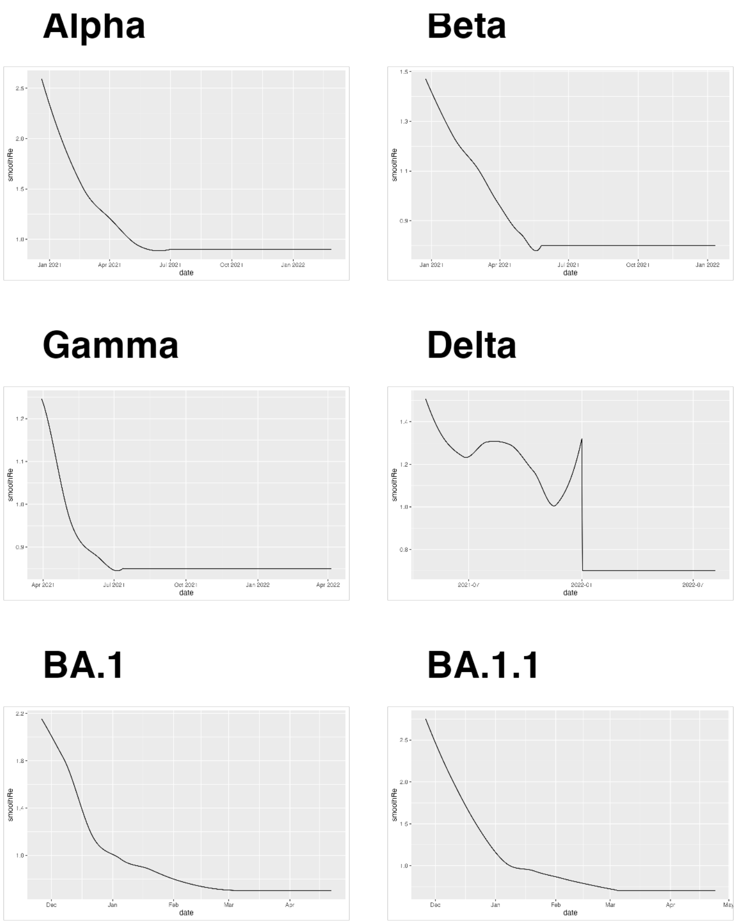

Fig. S26. Epochal R_t values used in simulations. Epochal R_t specified in the simulation is the median instantaneous R_t across sublineages, with truncated right tails as sublineage R_t values are unstable at later stages of epidemic.

Table S4. Characteristics of observed introductions informing stochastic simulations. Mean and standard deviation of sampled sublineage size fitted with gamma distributions, and maximum sampled size. Lifespan is the time between first and last sampled cases. Percentage singletons of introductions during intervention (and for Delta, during early and late periods). Sequences sampled per estimated variant case.

	**Mean size**	**sd size**	**Max size**	**Max lifespan**	**Percentage singletons**	**Sequence sampled per case**
**Alpha**	73.4	44.0	5061	327	77.4%	0.081
**Beta**	144.3	426.8	1827	322	65.3%	0.077
**Gamma**	125.4	1334.9	15480	355	87.4%	0.144
**Delta**	76.0	680.5	11016	313	Early: 64.1%<br>Late: 82.6%	0.050
**BA.1**	20.6	249.3	9744	124	80.8%	0.034
**BA.1.1**	22.6	291.0	16520	126	71.1%	0.030

Counterfactual modeling of cases averted with stochastic branching processes

We applied stochastic branching processes to simulate sublineage outbreaks, similarly to (Althaus 2015; Grubaugh et al. 2017; Riou and Althaus 2020; Reichmuth et al. 2022). For each variant, we simulated 20,000 outbreaks, which ran until infected cases were zero, cumulative infected cases exceeded the maximum expected outbreak size (maximum observed sublineage size / expected diagnosis per case / sequence per diagnosis), or lifespan (time from first to last case) exceeded maximum observed sublineage lifespan. Each introduction initiates a branching process where the number of descendants for each infected individual are drawn from a negative binomial distribution described by mean=R_t, dispersion parameter=k_t, and the time between successive infections, which was drawn from a gamma-distributed serial interval with mean drawn from uniform distribution of 4.7+/-0.1 days, standard deviation, sd=2.9+/-0.1 days (Nishiura et al. 2020; Riou and Althaus 2020; Susvitasari et al. 2023). For each generation, k_t was drawn from a uniform distribution from 0.1 to 0.3 (Endo et al. 2020), and R_t was specified as the epochal R_t, which was calculated as the median R_t across variant sublineages, excluding data with confidence interval wider than ten and dampened to a constant value of 0.8 after dropping below the last observed epidemic peak (Fig. S25, S26). Unstable and high R_t during early epidemics (which could lead to many large sublineages and no small to medium sublineages) was dealt with by drawing from a binomial distribution with 50% success probability at each generation, and for successes during which epochal R_t was greater than 1, we subtracted a value sampled from a uniform distribution between 0.1 and 0.5. For Beta, this was turned off as it was not required to recapitulate empirically-observed sublineage size distribution. For Alpha, when R_t was greater than 2 during the very early epidemic, the uniform distribution was 0.5-1, again, to prevent unrealistically frequent large outbreaks and to reflect the tendency of random generations to go extinct more rapidly.

We applied stochastic case ascertainment (diagnosed given infected) by drawing from a binomial distribution for each infected, with probability of success equal to the ascertainment rate, sampled from a truncated log-normal distribution (mean=log(expected diagnoses per infected), sd=0.1, minimum=0.1, maximum=0.9). We set default expected cases per infected as 0.1 for Alpha and Delta, 0.3 for Beta, 0.2 for Delta (Russell et al. 2020; Dougherty et al. 2021). Undiagnosed cases were excluded to reduce memory. Diagnoses were sampled with sampling probability drawn from a log-normal distribution with mean=log(sequences per diagnosis), sd=0.1, where the expected sequences per diagnoses was empirically calculated. Within each simulation, introduction date was sampled uniformly within the early and late periods of restrictions, with probability of being in either period equal to proportion of introductions averted in each period.

Simulations were filtered to exclude those that were unsampled (majority of outbreaks), exceeded the maximum lifespan from first to last sample (Table S4), or exceeded total infected size, equal to (maximum size of observed sublineages) / (sequences per diagnosis) / (diagnoses per infected). We also enforced the percentage of singletons (with one sampled case) to reflect the proportion of singletons among introductions observed up until the end of the restriction (Table S4). With remaining simulations, we made 100 draws of N averted introductions (without replacement within a draw), setting N to the mean, lower, or upper 95% confidence limit of averted introductions. We evaluated differences in cumulative and maximum daily incidence in the absence of travel restrictions. Percentage additional cases was calculated as the ratio of cumulative incidence predicted (averted) to observed (estimated variant cases based on provincial RT-PCR diagnoses and variant frequency). Introductions and cases averted were calculated for overall interventions and for early and late periods, and were reported overall and normalized to restriction duration.

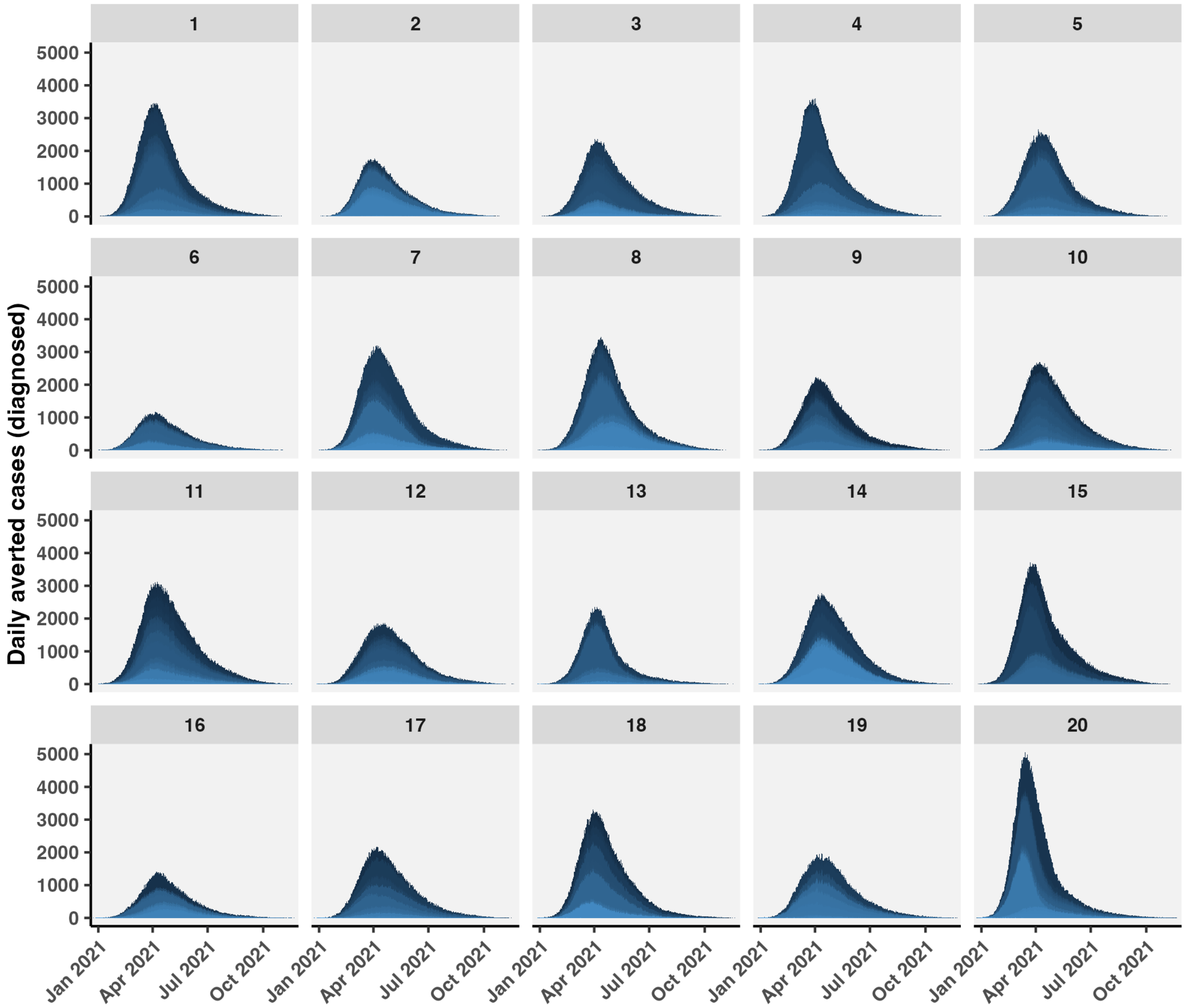

Fig. S27. Daily averted diagnosed cases across 20 of 100 draws for Alpha. Each draw includes simulation for each introduction averted. Colors represent outbreaks resulting from unique introductions.

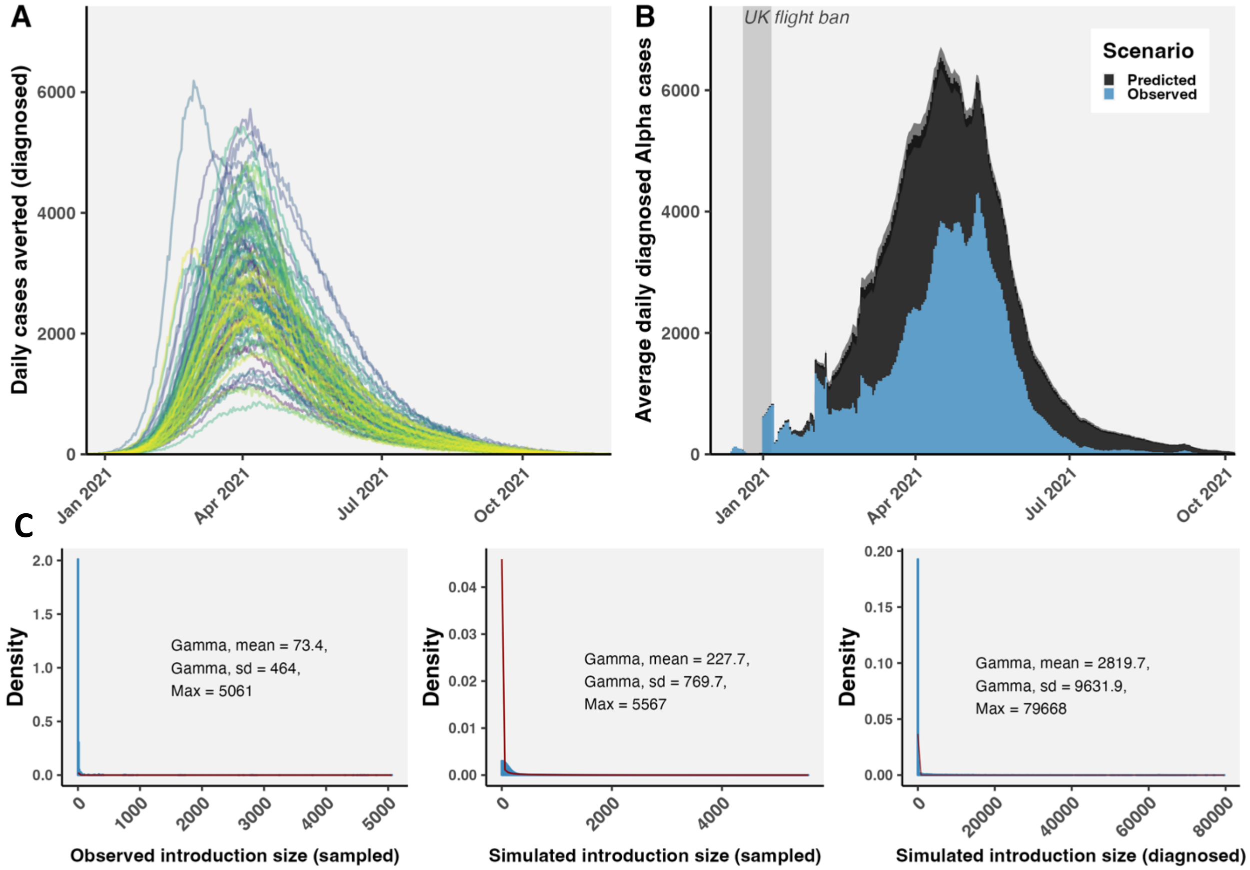

Fig. S28. Stochastic branching processes to simulate diagnosed cases averted for Alpha. (A) Total daily diagnosed cases summed across averted introductions for 100 draws. **(B)** Daily incidence observed and additional averted cases, representing median and 95% confidence interval (CI) daily cases averted. **(C)** Distribution of observed introduction sizes (sampled) and simulated introduction sizes (sampled and diagnosed), annotated with gamma distribution mean and standard deviation, as well as maximum size.

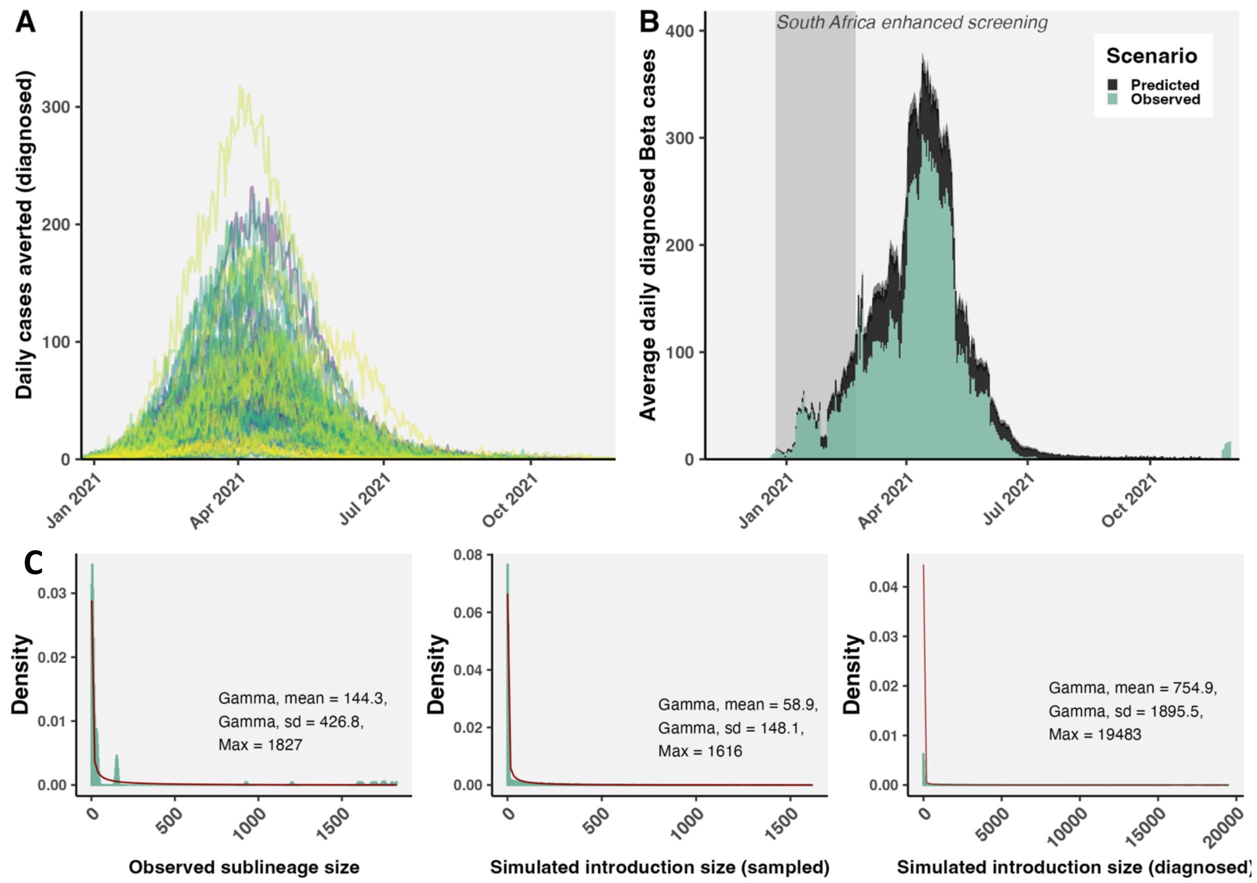

Fig. S29. Stochastic branching processes to simulate diagnosed cases averted for Beta. (A) Total daily diagnosed cases summed across averted introductions for 100 draws. **(B)** Daily incidence of observed and additional averted cases, representing median and 95% CI daily cases averted. **(C)** Distribution of observed introduction sizes (sampled) and simulated introduction sizes (sampled and diagnosed), annotated with fitted gamma distribution mean and standard deviation, as well as maximum size.

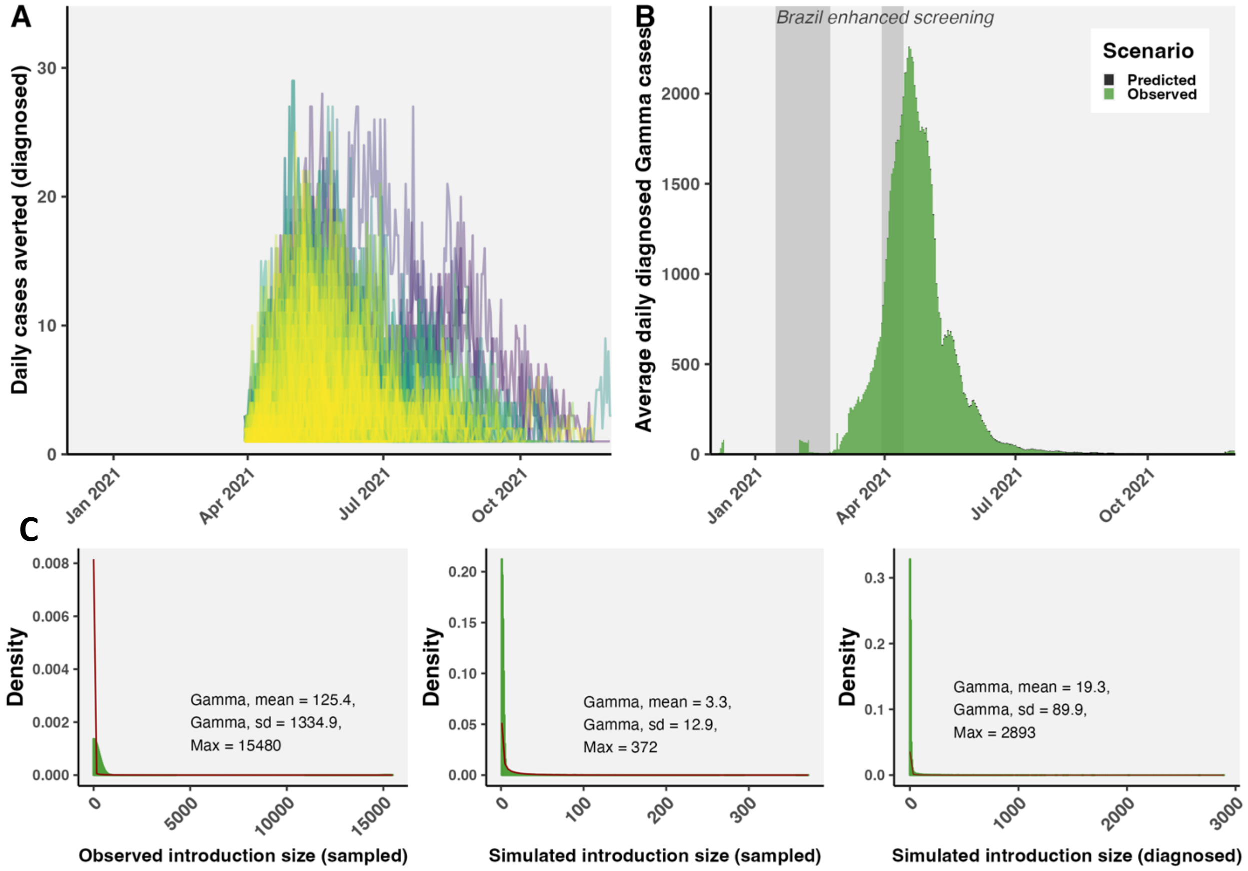

Fig. S30. Stochastic branching processes to simulate diagnosed cases averted for Gamma. (A) Total daily diagnosed cases summed across averted introductions in the second intervention for 1000 draws. **(B)** Daily incidence of observed and additional averted cases, representing median and 95% CI daily cases averted. **(C)** Distribution of observed introduction sizes (sampled) and simulated introduction sizes (sampled and diagnosed), annotated with fitted gamma distribution mean and standard deviation, as well as maximum size.

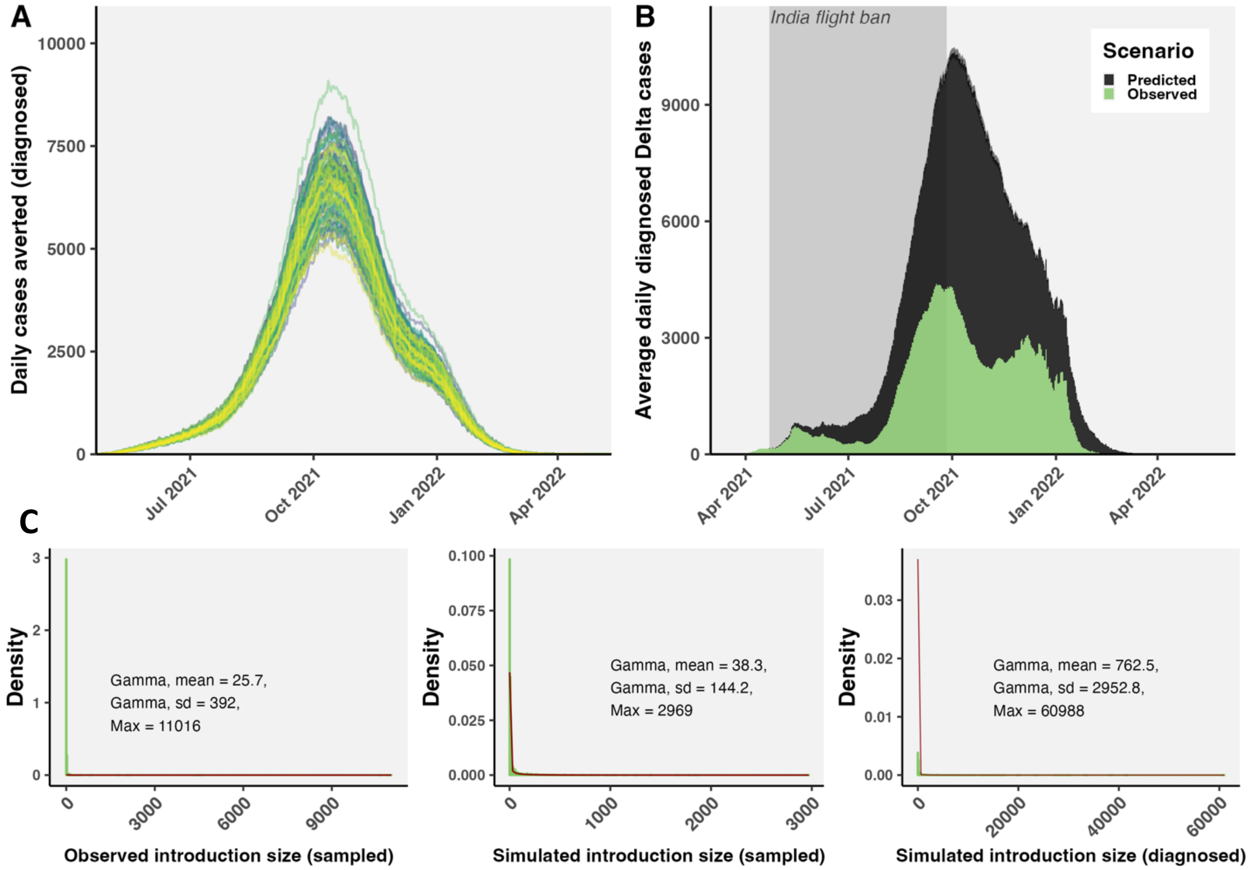

Fig. S31. Stochastic branching processes to simulate diagnosed cases averted for Delta. (A) Total daily diagnosed cases summed across averted introductions for 100 draws. **(B)** Daily incidence of observed and additional averted cases, representing median and 95% confidence interval daily cases averted. **(C)** Distribution of observed introduction sizes (sampled) and simulated introduction sizes (sampled and diagnosed), annotated with gamma distribution mean and standard deviation, as well as maximum size.

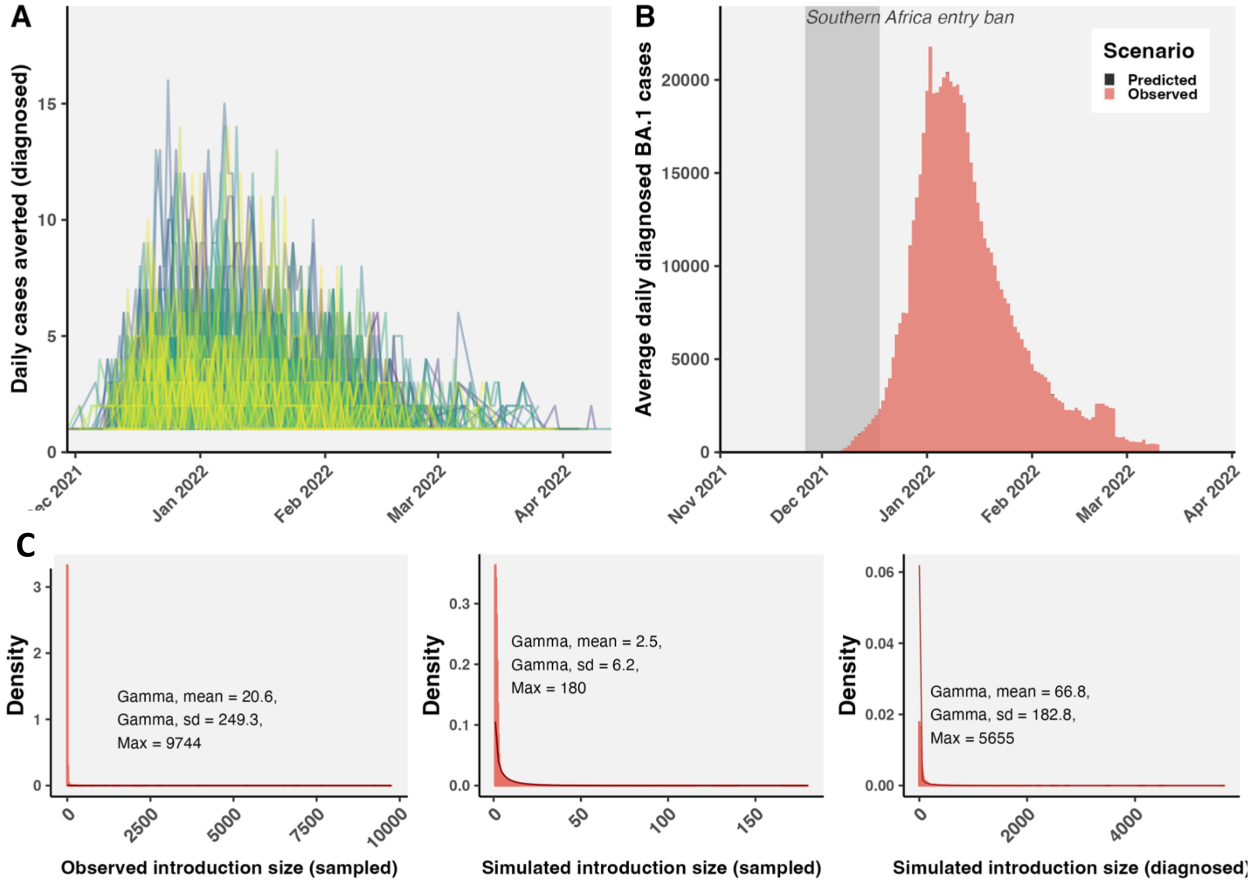

Fig. S32. Stochastic branching processes to simulate diagnosed cases averted for BA.1. (A) Total daily diagnosed cases summed across averted introductions for 100 draws. **(B)** Daily incidence of observed and additional averted cases, representing median and 95% CI daily cases averted. **(C)** Distribution of observed introduction sizes (sampled) and simulated introduction sizes (sampled and diagnosed), annotated with fitted gamma distribution mean and standard deviation, as well as maximum size.

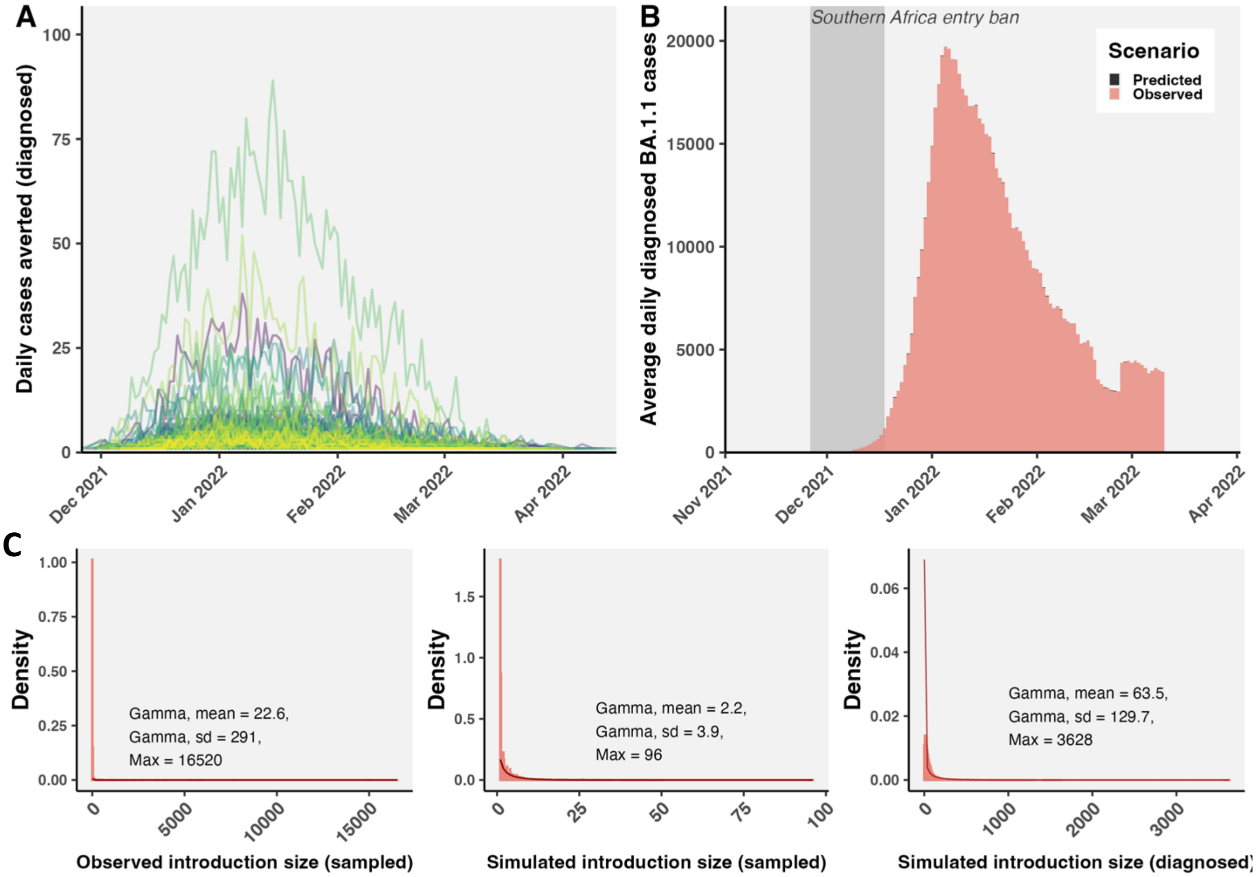

Fig. S33. Stochastic branching processes to simulate diagnosed cases averted for BA.1.1. (A) Total daily diagnosed cases summed across averted introductions for 100 draws. **(B)** Daily incidence of observed and additional averted cases, representing median and 95% CI daily cases averted. **(C)** Distribution of observed introduction sizes (sampled) and simulated introduction sizes (sampled and diagnosed), annotated with fitted gamma distribution mean and standard deviation, as well as maximum size.

Sampling size and deme sensitivity analyses

We evaluated the robustness of our results to subsample size and geographic deme grouping by further subsampling one of ten subsamples from the primary analysis (with temporal outliers removed) for Alpha, Beta, and Delta variants to 500, 1000, 5000, or 10,000 total sequences. Secondary subsampling was conducted using the same algorithm for temporally-distributed sampling with augmented sampling of sparse months based on proportional case contributions (Fig. S8) with 50% of sequences from global and Canadian pools. Additional sequences from parental lineages were also sampled: one B sequence (ref. seq. Wuhan-hu-1) and five random samples of B.1 (all) and B.1.1 (Alpha only). This was repeated five times for each sample size. ML time-scaled phylogenetic trees were inferred for each subsampled alignment, which were used for ML ancestral state reconstruction, specifying one of two reduced demes sets. Whereas in the primary analysis, we used ‘all demes’, representing global regions and individual Canadian provinces (25-27 demes), in the sensitivity analysis, we used ‘more demes’ (7-9 demes),

representing continents and highest contributing Canadian provinces and regions separate, or 'less demes' (3-4 demes), representing Canada, global (all except focal source), and the focal source. We repeated the ancestral reconstruction for primary subsample trees (n=50,000) with 'more' and 'less' demes. Grouping of demes in the primary subsample for a representative subsample was summarized for Alpha (Table S5), Beta (Table S6), and Delta (Table S7).

Table S5. Demes in the Alpha phylogeography sensitivity analyses for a representative subsample of 50,015 clean sequences. 'All demes' (n=25 demes), 'more demes' (n=8), 'less demes' (n=3).

All demes	n_all	More demes	n_more	Less demes	n_less
Canada_Quebec	5827	Canada_Other	9275	Canada	25,001
Canada_Alberta	1620				
Canada_Saskatchewan	1611				
Canada_Manitoba	141				
Canada_Nova Scotia	38				
Canada_New Brunswick	28				
Canada_Newfoundland and Labrador	10				
Canada_Ontario	8326	Canada_Ontario	8326		
Canada_British Columbia	7400	Canada_British Columbia	7400		
Europe	12654	Europe	14,363	Global	20,029
Germany	900				
France	423				
Sweden	197				
Spain	151				
Switzerland	25				
Denmark	13				
USA	4522	Americas	4558		
North America	19				
South America	13				
Brazil	4				
Asia	726	Asia	1099		
Japan	339				
India	34				
Africa	9	Africa	9		
UK (focal source)	4985	UK	4985	UK	4985

Table S6. Demes in the Beta phylogeography sensitivity analyses for a representative subsample of 26,407 clean sequences. 'All demes' (n=27 demes), 'more demes' (n=8), 'less demes' (n=4). Although the focal source is South Africa, Africa was also kept separate throughout.

All demes	n_all	More demes	n_more	Less demes	n_less
Europe	4701	Europe	8749	Global	15,160
France	2105				
Spain	1021				
Germany	545				
Sweden	314				
Switzerland	55				
Denmark	8				
Asia	4972	Asia	5118		
India	109				
Oceania	26				
Japan	11				
USA	1093	Americas	1100		

North America	4				
South America	2				
Brazil	1				
UK	193	UK	193		
Africa	5186	Africa	5186	Africa	5186
South Africa	4647	South Africa	4647	South Africa	4647
Canada_Ontario	746	Canada_Ontario	746		
Canada_Quebec	339				
Canada_British Columbia	152				
Canada_Alberta	106				
Canada_Manitoba	28	Canada_Other	668	Canada	1414
Canada_New Brunswick	27				
Canada_Nova Scotia	9				
Canada_Newfoundland and Labrador	6				
Canada_Saskatchewan	1				

Table S7. Demes in the Delta phylogeography sensitivity analyses for a representative subsample of 49,998 clean sequences. ‘All demes’ (n=27), ‘more demes’ (n=9), and ‘less demes’ (n=3).

All demes	n_all	More demes	n_more	Less demes	n_less
Canada_British Columbia	11,164	Canada_British Columbia	11,164		
Canada_Ontario	7105	Canada_Ontario	7105		
Canada_Alberta	4019				
Canada_Quebec	1505				
Canada_Saskatchewan	880			Canada	25,001
Canada_Manitoba	168	Canada_Other	6732		
Canada_New Brunswick	134				
Canada_Nova Scotia	20				
Canada_Newfoundland and Labrador	6				
USA	10,523				
North America	62	Americas	10,652		
Brazil	45				
South America	22				
Europe	5144				
Germany	395				
France	195				
Spain	64	Europe	5841		
Denmark	21			Global	21,121
Switzerland	16				
Sweden	6				
UK	3578	UK	3578		
Asia	890		990		
Japan	95	Asia	990		
Oceania	5		990		
Africa	34	Africa	60		
South Africa	26				
India	3876	India	3876	India	3876

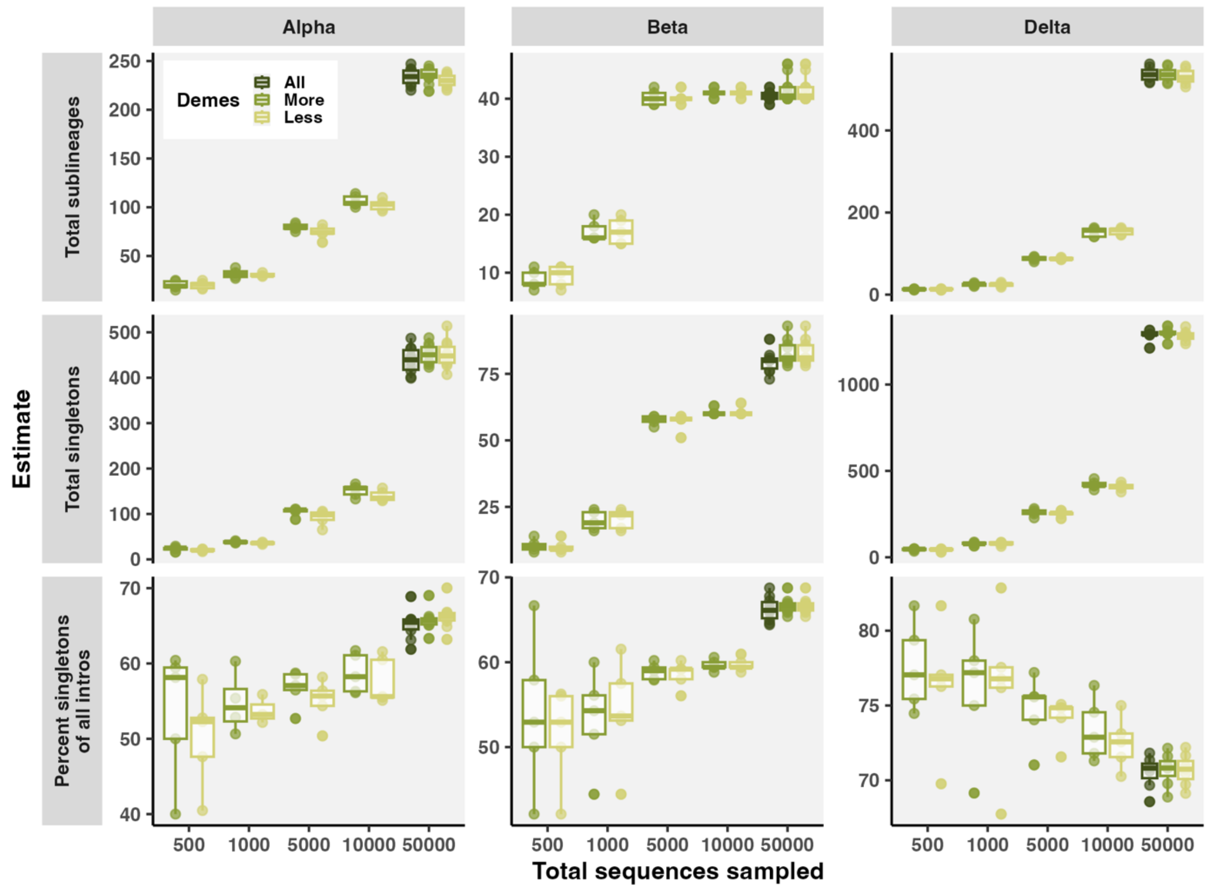

Fig. S34. Sensitivity of total inferred introductions from all sources to sample size and number of demes in the maximum likelihood analysis for Alpha, Beta, and Delta. Box and whisker plots show median (thick line), first and third quartiles (hinges), and 1.5-times interquartile range (whiskers), with individual points overlaid. Demes include 'all' geographies from the primary analysis (n=25-27 demes), 'more' geographies (n=8-9), or 'less' geographies (n=3-4),

Total inferred sublineages and singletons scaled relatively linearly with sample size (Fig. S34). Beta was an exception, as there were only 1414 clean Beta sequences available for Canada, which were all included for samples with more than 5000 total sequences; adding global sequences did not increase identified sublineages. Inferring more demes compared to less led to slightly more sublineages and singletons inferred, most notably for Alpha. Proportion singletons of introductions increased with larger sample sizes for Alpha and Beta (sublineage identification is near saturated), but not Delta, for which more sublineages were identified with larger subsamples (sublineage identification not exhaustive).

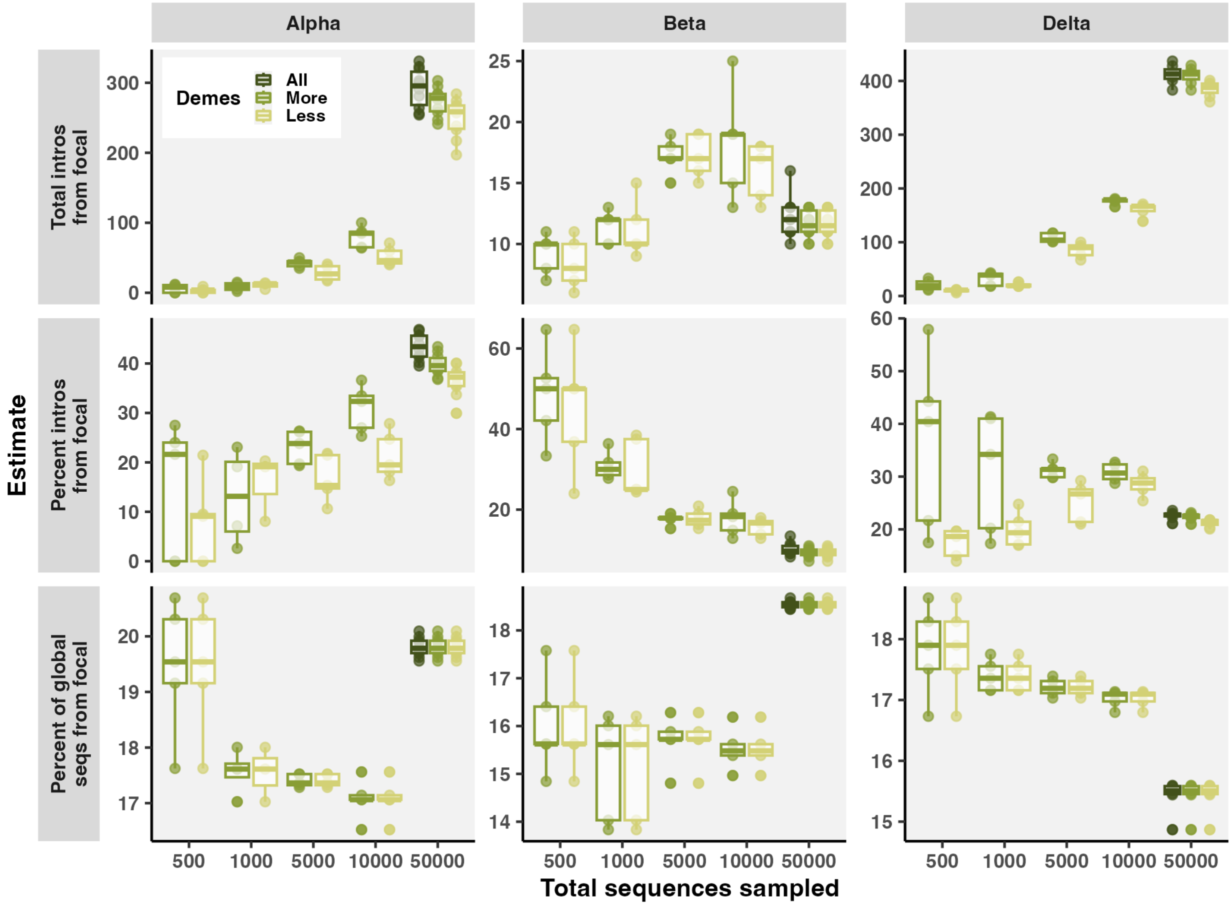

Fig. S35. Sensitivity of total and percentage inferred introductions from focal source to sample size and demes for Alpha, Beta, and Delta. Introductions include sublineages and singletons over the entire study period. Percentage of global sequences that were sampled from focal source shown in bottom row.

Total inferred introductions from the focal source roughly scaled with sample size for Alpha and Delta, but not Beta (Fig. S35). The more deme groups there were (all>more>less), the more total introductions and a higher percentage from the focal source there were, most markedly for smaller sample sizes. In the primary analysis (n=50,000), there were slightly fewer total introductions and lower percentage of introductions from the focal source when fewer demes were specified with Alpha and Delta. For Alpha, smaller sample sizes led to lower estimates of percentage of introductions from focal than the primary analysis, whereas the opposite was true for Beta and Delta. For Delta, the percentage of introductions from focal source overall was within 10% of the primary analysis for sample sizes of 5000 and 10,000. The focal source for Delta (India) represented a higher percentage of global sequences in smaller subsamples, as sequences per case are more normalized for small sample sizes. This suggests proportion of introductions from India for Delta in the primary analysis were lower bounds (as India was under-represented). By contrast, the focal source for Alpha (the UK) also contributed the most sequences per case;

therefore, its representation is lower for smaller sample sizes, except at the smallest sizes, where stochasticity leads to wide confidence intervals.

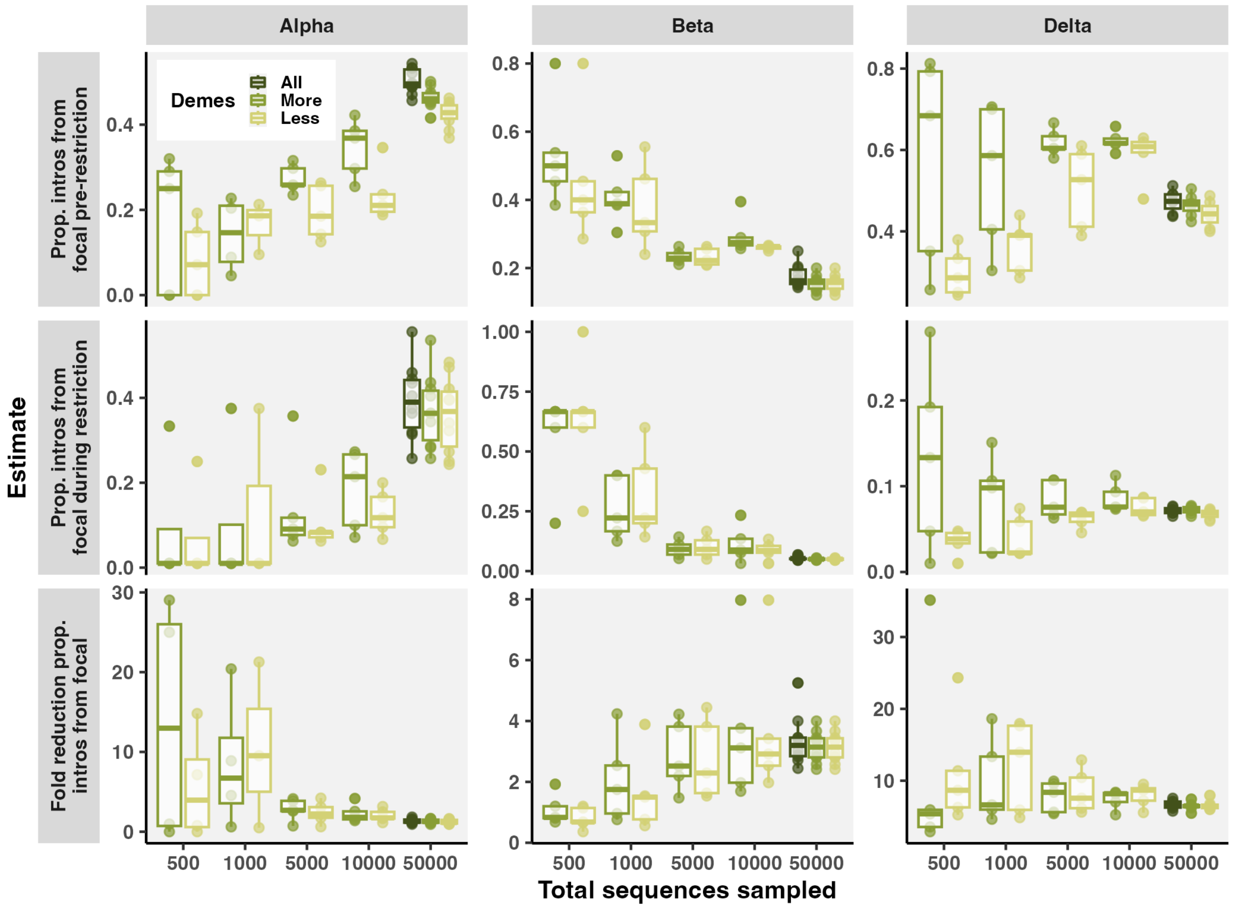

Fig. S36. Sensitivity of the proportion of introductions from focal source to sample size and demes for Alpha, Beta, and Delta. Rows represent proportion of introductions (sublineages and singletons together) from focal source compared to all sources before and during the restriction, and fold-reduction in the proportion of introductions from focal source during vs. pre-restriction.

Fold-reduction in the proportion of introductions from focal source was relatively robust to sample size and very robust to deme groupings for all variants, especially for subsamples with at least 5000 sequences (Fig. S36). Smaller subsamples had wide confidence intervals due to stochasticity in the composition of sequences and geographies.

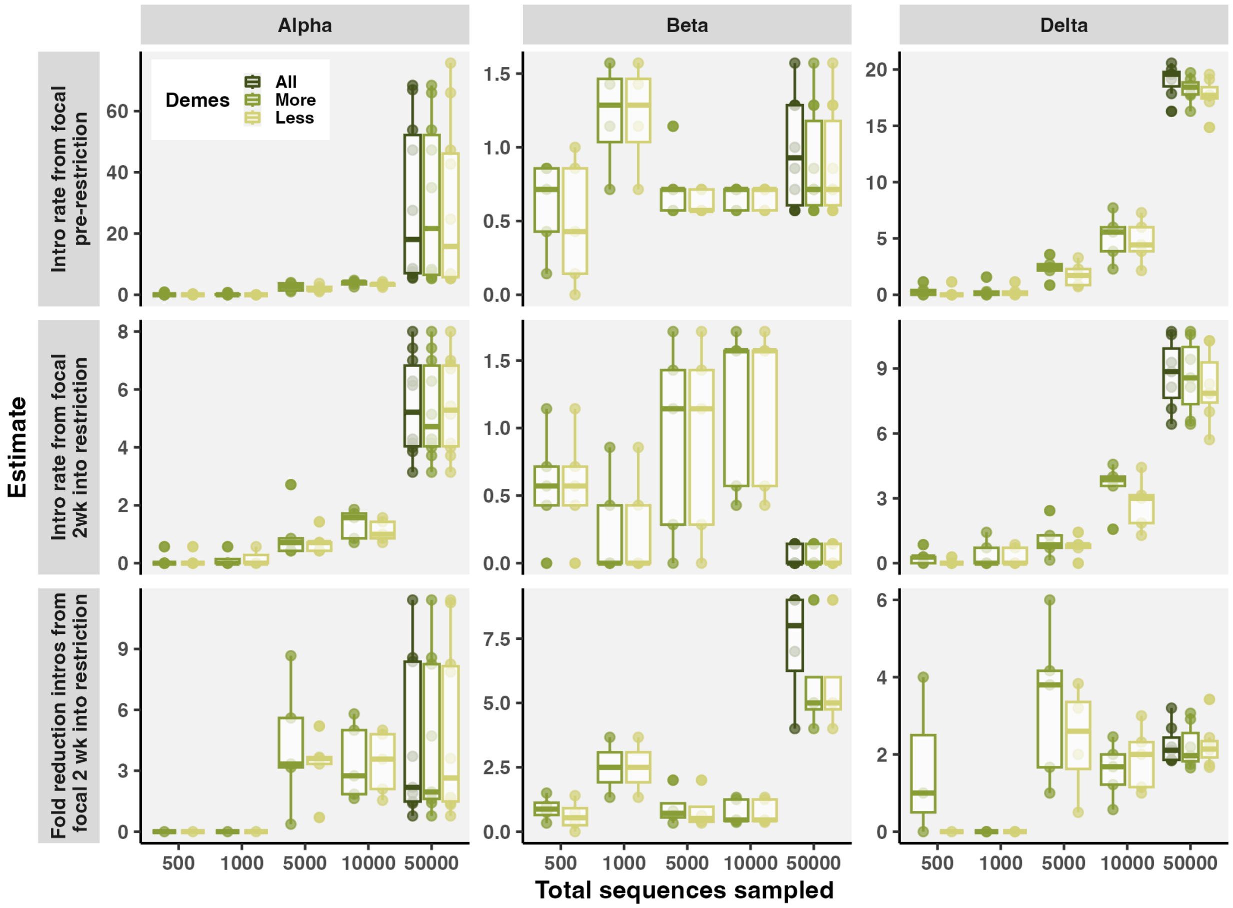

Fig. S37. Sensitivity of inferred introductions rates from focal source to sample size and demes for Alpha, Beta, and Delta. Rows represent introduction rate (sublineages and singletons together) from focal source pre-restriction and two weeks into the restriction, and fold-reduction in the introductions from focal source two weeks into vs. pre-restriction.

Estimates of introduction rates from focal sources were sensitive to subsample size (Fig. S37). Smaller subsamples failed to detect early introductions before restrictions were imposed, in particular, but also later introductions. In several small subsample sizes, introduction rates of zero were inferred before and two weeks into restrictions. For Beta especially, where there were relatively fewer introductions even in the primary analysis, detection of introductions was unstable for small subsamples. The fold-reduction in the introduction rate from focal two weeks into the restriction was unstable for small sample sizes when comparing small or zero rates; for Delta, estimates for samples larger than 5000 were quite stable, ranging from two- to four-fold-reduction, and for Alpha, estimates of the fold-reduction for samples larger than 5000 were also relatively stable, at around two- to three-fold-reduction.

The correlation of monthly estimated variant cases and sequences sampled was comparable across subsample sizes for Canadian provinces (Fig. S38) and global regions (Fig. S39), as a result of applying the same subsampling algorithm, and all represent a marked improvement upon the raw data (Fig. S12, S13).

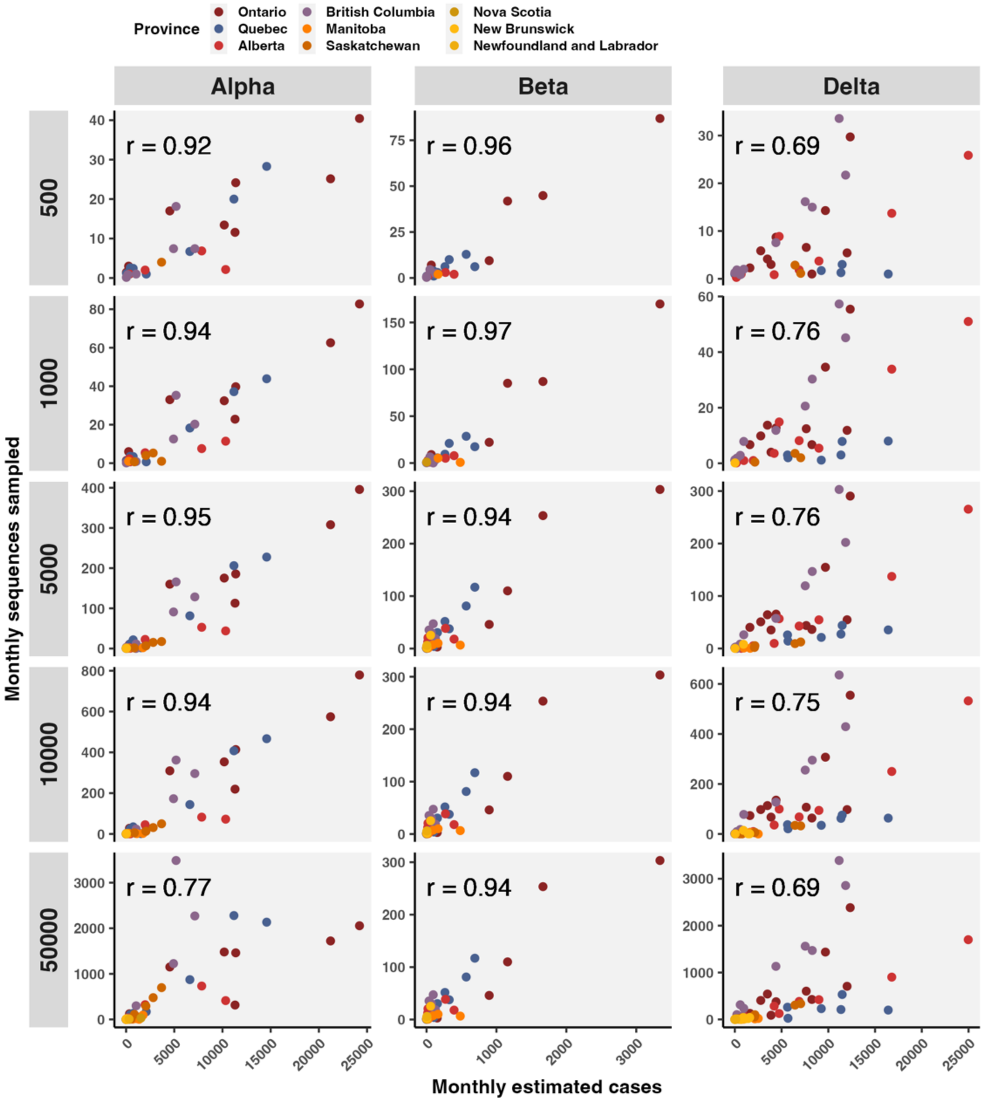

Fig. S38. Correlation between monthly sequences sampled and estimated variant cases across Canadian provinces for various subsample sizes.

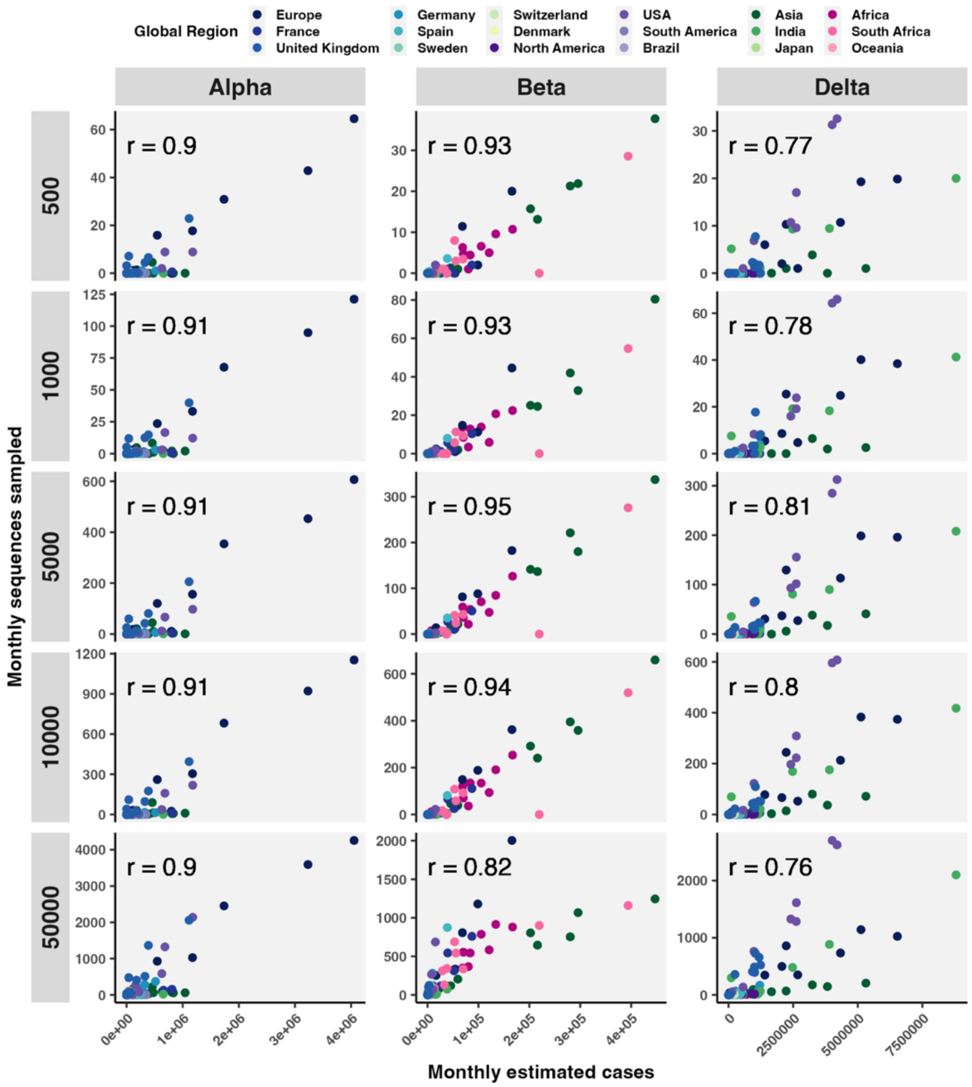

Fig. S39. Correlation between monthly sequences sampled and estimated variant cases across global regions for various subsample sizes.

Bayesian phylogeographic sensitivity analyses

To assess the robustness of estimated importation dynamics inferred with maximum likelihood, we compared relative importation rates with a Bayesian discrete trait analysis in BEAST v1.10.4 with BEAGLE (Minin and Suchard 2008; Lemey et al. 2009; Ayres et al. 2012; Suchard et al. 2018). For computational feasibility, secondary subsamples with 500 sequences were utilized for this comparison. We assessed temporal signal using Tempest (Rambaut et al. 2016) and pipeline described in methods; there were no further temporal outliers and clock signals were acceptable (linear fit adjusted R-squared: 0.260 for Alpha, 0.23 for Beta, 0.45 for Delta).

Model selection was conducted with path sampling (Baele et al. 2012b, 2012a) (chain length=1,000,000; path steps=100) to compare strict (log-normal, LN [mean=8e-4, sd=0.001]) and relaxed uncorrelated molecular clocks (mean: LN [8e-4, 0.001]; stdev: LN [0.001, 0.001]), tree priors (coalescent exponential (population size: 1/x [0, 1]; growth rate: Laplace [0.09, 0]) and Gaussian Markov Random Field (GMRF) Skygrid (precision: Gamma [0.001, 1000])) (Minin et al. 2008; Gill et al. 2013), and substitution models HKY (transition-transversion parameter: LN [1, 1.25]) and GTR (transition rate parameters: Dirichlet [1,1]) with empirical base frequencies, four categories of gamma-distributed rate heterogeneity (shape parameter: Exp [0.5]) and proportion invariant sites (Unif [0, 1])) for each variant. MCMC chains ran for 100 million states, sampled every 10,000 states. Convergence was assessed using Tracer (Rambaut et al. 2018) with equivalent sampling size (ESS) more than 200 after 10% burn-in. Superior marginal log likelihoods were estimated for GTR over HKY, GMRF over coalescent exponential, and were similar for relaxed and strict clocks. Subsequent DTA models specified GTR substitution model, GMRF Skygrid coalescent tree prior, and a strict clock.

As described above, we specified two sets of demes: 'More demes' with 7-9 groups including focal source, continents, Canada, and largest contributors separate; and 'less demes' included three (Alpha; Delta) or four (Beta) groups (Canada, focal region, and global outside focal) (Table S5-7). Tips' demes were allocated as partitions with symmetric substitution models, inferred social network with Bayesian stochastic search variable selection (BSSVS) (Lemey et al. 2009) and strict clock models. We specified GMRF Skygrid coalescent (Gill et al. 2013) tree prior (Gamma [0.001, 1000]) with 20 equally-spaced epochs. For discrete traits, we reconstructed state change counts and the complete change history on the tree (Minin and Suchard 2008). Priors included GTR transition rate parameters (Dirichlet [1, 1]), gamma shape parameters alpha (Exp [0.5]), proportion invariant sites (Unif [0, 1]), strict clock rate (LN [4e-4, 4e-4]), substitution rates for deme states as default CTMC rate reference (Ferreira and Suchard 2008), Poisson-distributed number of non-zero rates for BSSVS, uniform discrete state frequencies, and discrete trait transition rates (Gamma [1, 1]). Maximum likelihood time-scaled trees were input as initial trees with fixed topology and internal node heights were sampled from the posterior. Three parallel MCMC chains were run with 200 million states logged every 20,000 states. Chains were merged following 10% burn-in and assessed for convergence in Tracer based on ESS >200 (Rambaut et al. 2018). Maximum clade credibility (MCC) trees were inferred following 10% burn-in using TreeAnnotater with median node heights (Drummond and Rambaut 2007). Highest posterior internal node states of MCC trees were used to identify introductions over time.

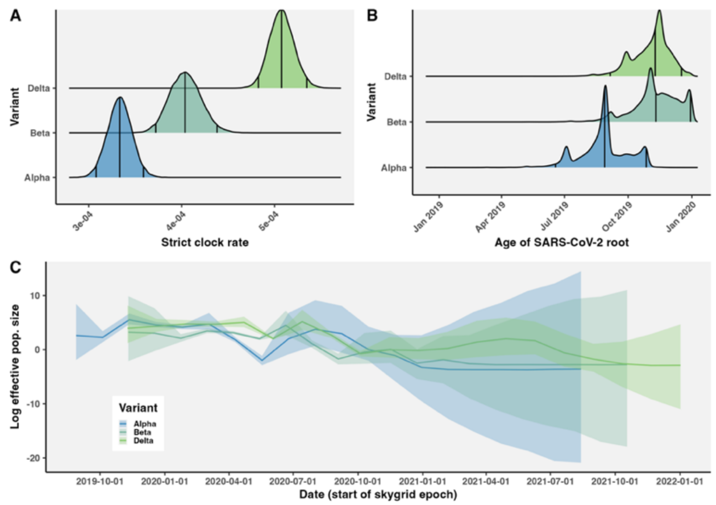

Fig. S40. Posterior distribution of clock rate, root date, and effective population size from GMRF Skygrid coalescent models. Median and 95% highest posterior density (HPD) range shown with vertical lines for **(A)** strict molecular clock rate and **(B)** root age. **(C)** Median and 95% HPD credibility interval of log effective population size across 20 equally spaced GMRF Skygrid epochs from root to most recently sampled tip.

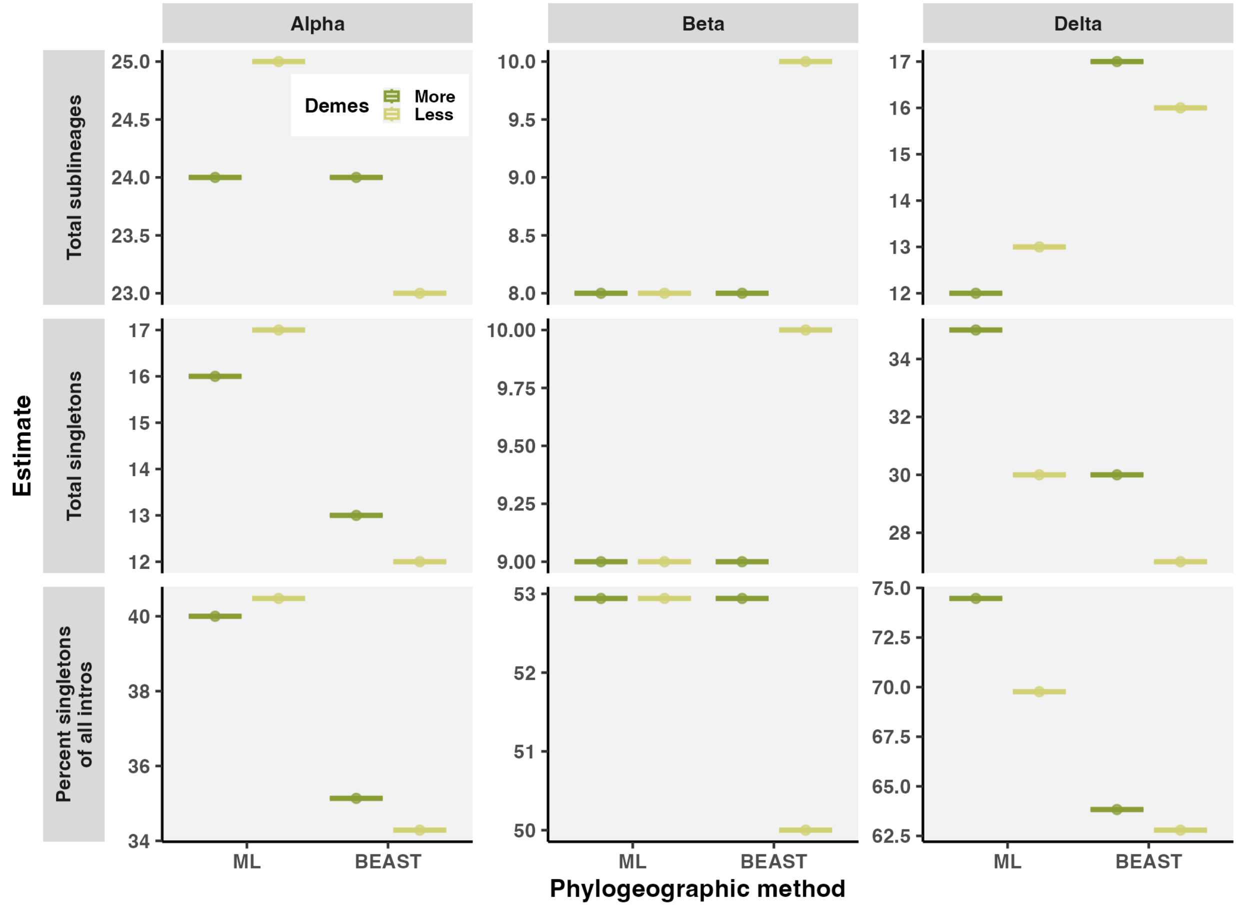

Fig. S41. Sensitivity of total inferred introductions from all sources to phylogeographic method (maximum likelihood, ML, and Bayesian) and number of demes for Alpha, Beta, and Delta (n=500). Comparison shown for a single subsample tree per variant.

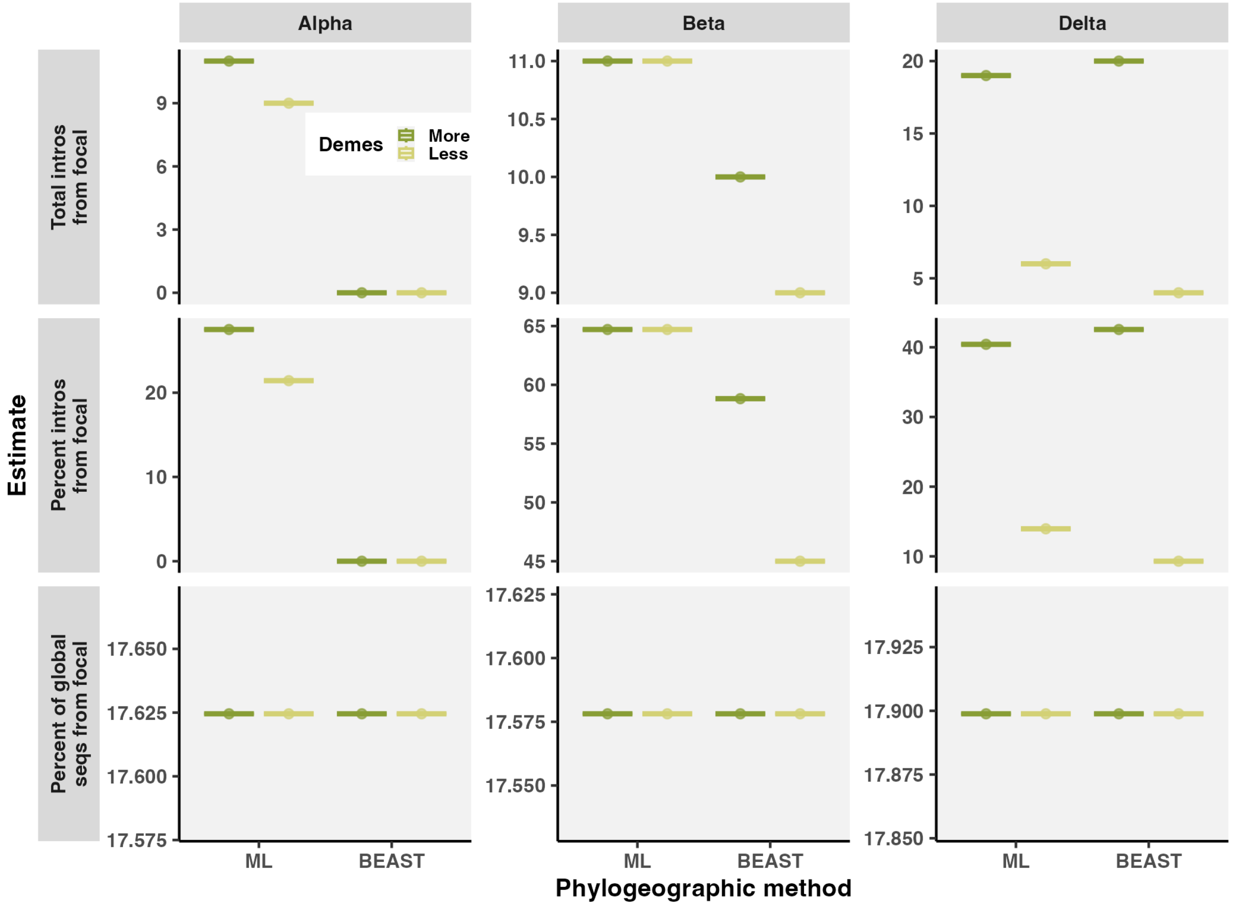

Fig. S42. Sensitivity of total and percentage inferred introductions from focal source to phylogeographic method and demes for Alpha, Beta, and Delta (n=500). Introductions include sublineages and singletons over the entire study period. Percentage of global sequences sampled from focal source in bottom row. Comparison shown for a single subsample tree per variant.

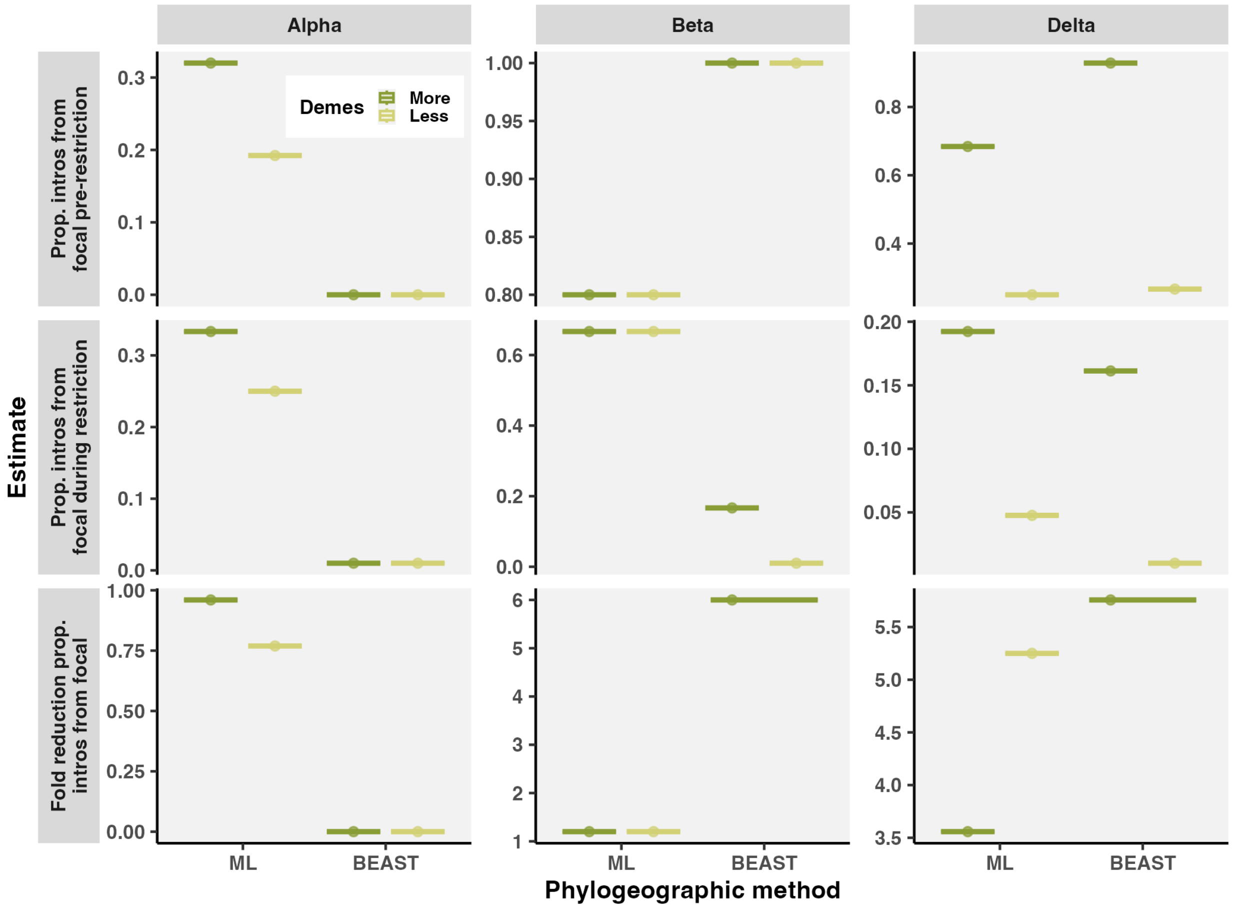

Fig. S43. Sensitivity of the proportion of introductions from focal source to phylogeographic method and demes for Alpha, Beta, and Delta. Rows represent proportion of introductions (sublineages and singletons together) from focal source compared to all sources before and during the restriction, and fold-reduction in the proportion of introductions from focal source during vs. pre-restriction. Comparison shown for a single subsample tree per variant.

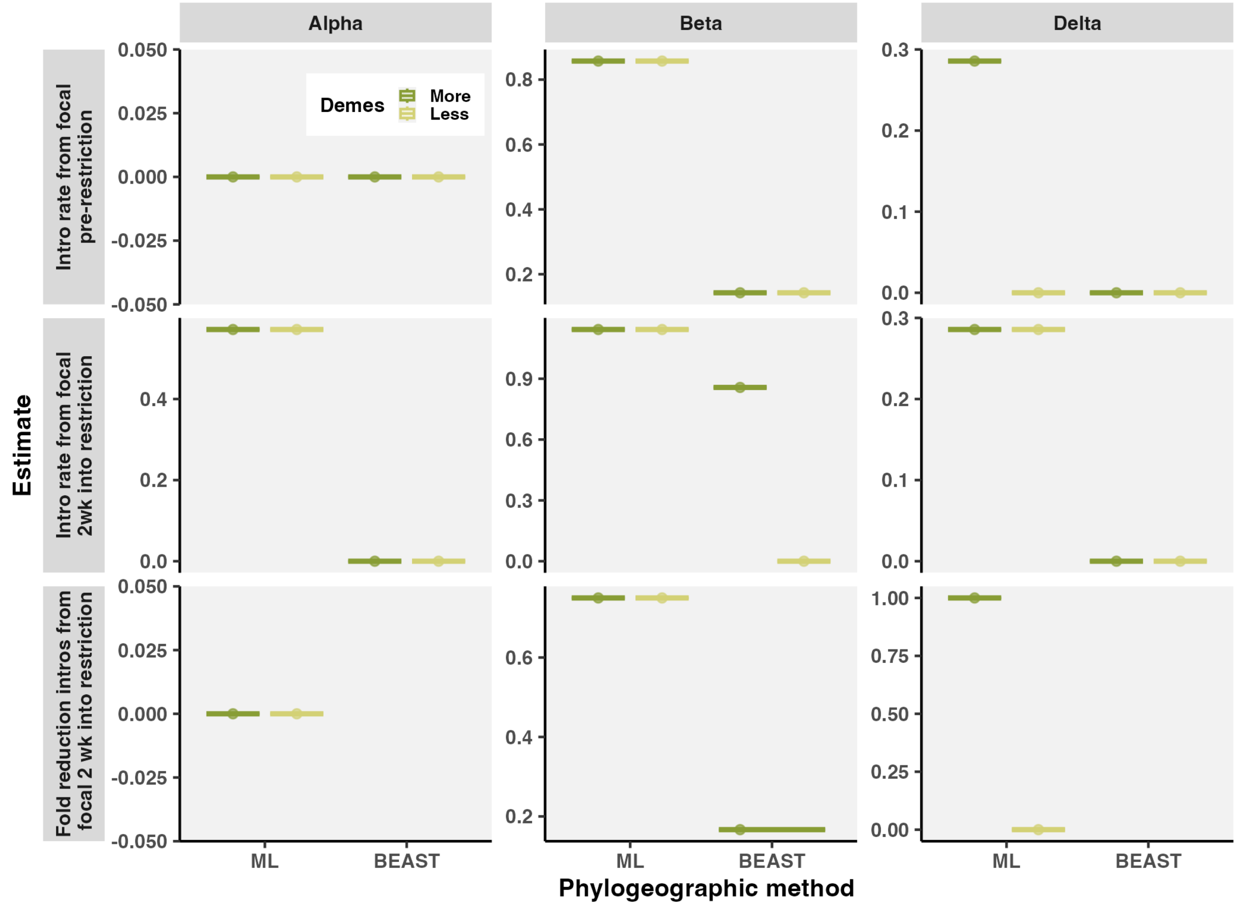

Fig. S44. Sensitivity of inferred introductions rates from focal source to phylogeographic method and demes or Alpha, Beta, and Delta (n=500). Rows represent introduction rate (sublineages and singletons together) from focal source pre-restriction and two weeks into the restriction, and fold-reduction in the introductions from focal source two weeks into vs. pre-restriction. Comparison shown for a single subsample tree per variant.

Supplementary Text

Synopsis of Canadian COVID-19 travel restrictions during the early variant pandemic

In elaboration of the timeline of federal COVID-19 travel restrictions in Canada (**Fig. 1D**), we compiled a synopsis of the timing of travel restrictions including entry requirements and variant measures from fall 2020 to spring 2022. Data was collated from a combination of resources, including the Canadian Institute for Health Information (CIHI 2021), various Government of Canada press releases, and a timeline of Canada Border Services Agency Border Measures (Canada Border Services Agency 2021b), and a timeline of border measures in Canada published by Piper et al. (Piper et al. 2022)

- December 9, 2020: Health Canada approved first COVID-19 vaccine (Health Canada 2020).
- December 20, 2020 -– January 6, 2021: Suspended all commercial and private passenger flights arriving from the United Kingdom suspended in response to Alpha (PHAC 2020).

- December 23, 2020 -– February 22, 2021: Canada Border Health Services Agency enhanced screening for travelers arriving from South Africa and referrals to PHAC in response to Beta (PHAC 2021c).
- January 7, 2021: Required all travelers aged five years or older to have proof of a negative COVID-19 molecular test within past 72 hours to board an aircraft (Canada Border Services Agency 2021b).
- January 15, 2021 – February 22, 2021: Enhanced screening questions and PHAC referrals for travelers coming from Brazil by air and land in response to Gamma (Canada Border Services Agency 2021b).
- January 21, 2021: Travelers allowed to board flights with prior positive COVID-19 test conducted 14 – 90 days before flight (Canada Border Services Agency 2021b).
- January 31, 2021 - April 30, 2021: Suspended flights to and from Mexico and the Caribbean (Transport Canada 2021a)
- February 1, 2021: All international flights must land in Toronto, Montreal, Calgary, or Vancouver (CIHI 2021).
- February 15, 2021: Testing requirement implement requiring foreign nationals arriving from the US to provide proof of a valid COVID-19 molecular test less than 72 hours before entry or prior positive COVID-19 molecular test 14-90 days before entry into Canada (Canada Border Services Agency 2021a).
- February 21, 2021: Travelers arriving by air required to submit evidence of pre-paid reservation for three-night stay at government authorized accommodation for quarantine; mandated to stay until first negative test result received (Canada Border Services Agency 2021b).
- February 22, 2021: All persons arriving by air or land required to submit quarantine plan and contact information electronically on the ArriveCAN app before boarding a plane to Canada or arriving at land border with limited exceptions (Canada Border Services Agency 2021a).
- March 30, 2021 – April 14, 2021: Reintroduced enhanced screening questions and PHAC referrals for travelers coming from Brazil in response to Gamma (Canada Border Services Agency 2021b).
- April 22, 2021 – May 21, 2021: Suspended all direct commercial and private passenger flights from Pakistan and India for 30 days. Passengers departing from India or Pakistan who arrive to Canada via indirect routes required to have negative COVID-19 test from the last country they travelled to before arriving in Canada (Transport Canada 2021b). Suspension was ultimately extended until September 26, 2021 for India and until June 8, 2021 for Pakistan.
- May 23, 2021: Extended suspension of flights from India and Pakistan (Canada Border Services Agency 2021b).

- July 5, 2021: Unvaccinated, but not vaccinated, travelers arriving by air required to stay at government-authorized hotel for three nights (of 14-day quarantine) (PHAC 2021a).
- July 5, 2021: Fully vaccinated travelers eligible to enter Canada not required to quarantine while awaiting on-arrival test result or complete a day-8 test. At any time after entry to Canada, if a fully vaccinated traveler tests positive or are exposed, local public health requirements including quarantine or isolation apply (PHAC 2021a).
- August 9, 2021: Removed requirement for hotel quarantine (Piper et al. 2022).
- August 9, 2021: Re-opened non-essential travel to Canada for fully vaccinated US citizens and permanent residents (no quarantine or day-8 test required) (Piper et al. 2022).
- August 9, 2021: International flights permitted at arrive at five additional Canadian airports (Halifax, Quebec, Ottawa, Winnipeg, and Edmonton) (Piper et al. 2022).
- August 9, 2021- September 21, 2021: Extended restriction on direct flights from India (Transport Canada 2021c).
- August 29, 2021: Restricted all direct commercial and private passenger flights from Morocco (Transport Canada 2021d).
- September 7, 2021: Reopened non-essential travel to Canada for all fully vaccinated foreign nationals (Piper et al. 2022).
- September 21, 2021 – September 26, 2021: Final extension on restriction of flights from India (Transport Canada 2021e).
- October 21, 2021: Lifted global advisory against non-essential travel, which was replaced by country specific advisories (Piper et al. 2022).
- November 30, 2021: Eliminated COVID-19 testing for fully vaccinated individuals departing and entering the country within 72 hours; expanded the list of accepted vaccines for the purpose of travel; valid COVID-19 tests no longer accepted as an alternative to vaccination for travel within Canada; new requirements for all exempt essential travelers, including the mandate to identify their vaccination status in ArriveCAN. Vaccination still required for travel within and out of Canada (PHAC 2021b).
- November 26, 2021 – December 18, 2021: Enhanced border measures for all travelers who had been to southern African nations (South Africa, Eswatini, Lesotho, Botswana, Zimbabwe, Mozambique, and Namibia) within the last 14 days; foreign nationals who had travelled to the region in the last 14 days not permitted entry; Canadian citizens and permanent residents subjected to enhanced testing, screening and quarantine (PHAC 2021c, 2021d, 2021f)
- November 30, 2021: Countries expanded to include Egypt, Malawi, and Nigeria (PHAC 2021d).
- January 15, 2022: Unvaccinated or partially vaccinated foreign national truck drivers coming to Canada from the US by land would not be allowed entry; unvaccinated or

partially vaccinated Canadian truck drivers would not be denied entry into Canada (PHAC 2022a).

- February 14, 2022: Federal government declared a public order emergency via the Emergency Act to disperse blockades assembled by the Freedom Convoy, a protest of truckers and anti-COVID-19 mandate supporters who occupied the capital for three weeks and blocked at least two major international borders (Government of Canada 2022).
- February 28, 2022: Easing of on-arrival testing for fully-vaccinated travelers with random testing and no quarantine required while awaiting test results (PHAC 2022b).
- *Study period end; data download date: March 22, 2022.*
- April 1, 2022: Fully vaccinated travelers were no longer required to provide a pre-entry COVID-19 test result to enter Canada by air, land or water; unvaccinated continued to provide a COVID-19 test result (PHAC 2022c).
- April 25, 2022: Border measures eased; unvaccinated and partially vaccinated children accompanied by a fully vaccinated guardian no longer required to complete a pre-entry COVID-19 test; fully vaccinated travelers no longer need to provide a quarantine plan; fully vaccinated travelers no longer have to wear a mask in public spaces, monitor or report symptoms, quarantine if a traveler in their group shows symptoms, or maintain a list of close contacts and locations visited. Passengers still required to use ArriveCAN (PHAC 2022d).
- October 1, 2022: ArriveCAN program ended and all entry requirements were dissolved. Vaccination, negative test, ArriveCAN, or masking no longer required for all travelers entering Canada by air, land, or water (PHAC 2022e).

SARS-CoV-2 VOC and VOI sublineage and singleton dynamics in Canada

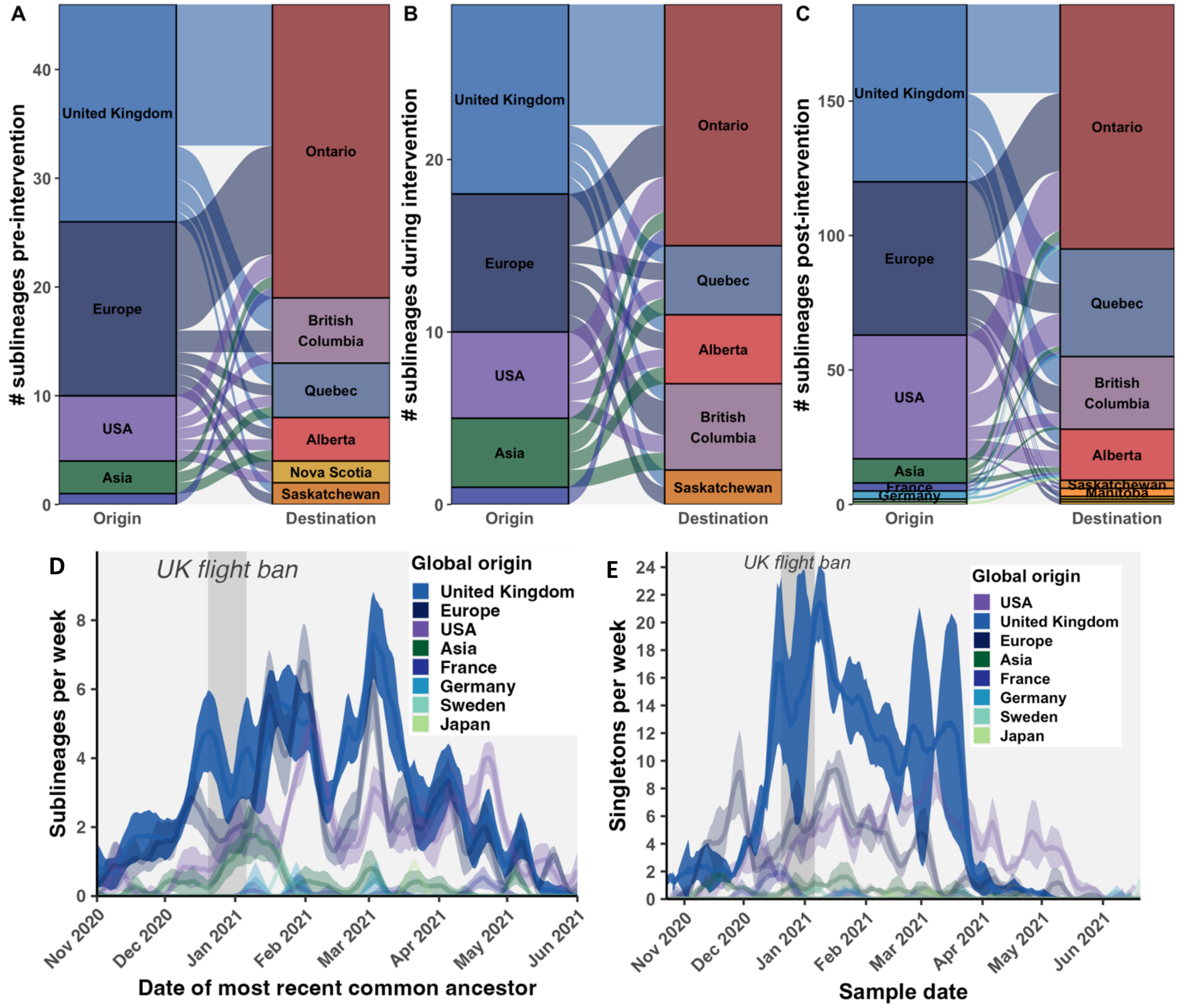

Fig. S45. **Alpha introduction dynamics in Canada.** Relative contributions of global origins and Canadian destinations to Alpha sublineage importations into Canada **(A)** before, **(B)** during, and **(C)** after the UK flight ban. Temporal dynamics of **(D)** Alpha sublineages and **(E)** singletons imported per week colored by global origins in the context of the UK flight ban. Ribbons denote 95% confidence intervals across 10 subsamples.

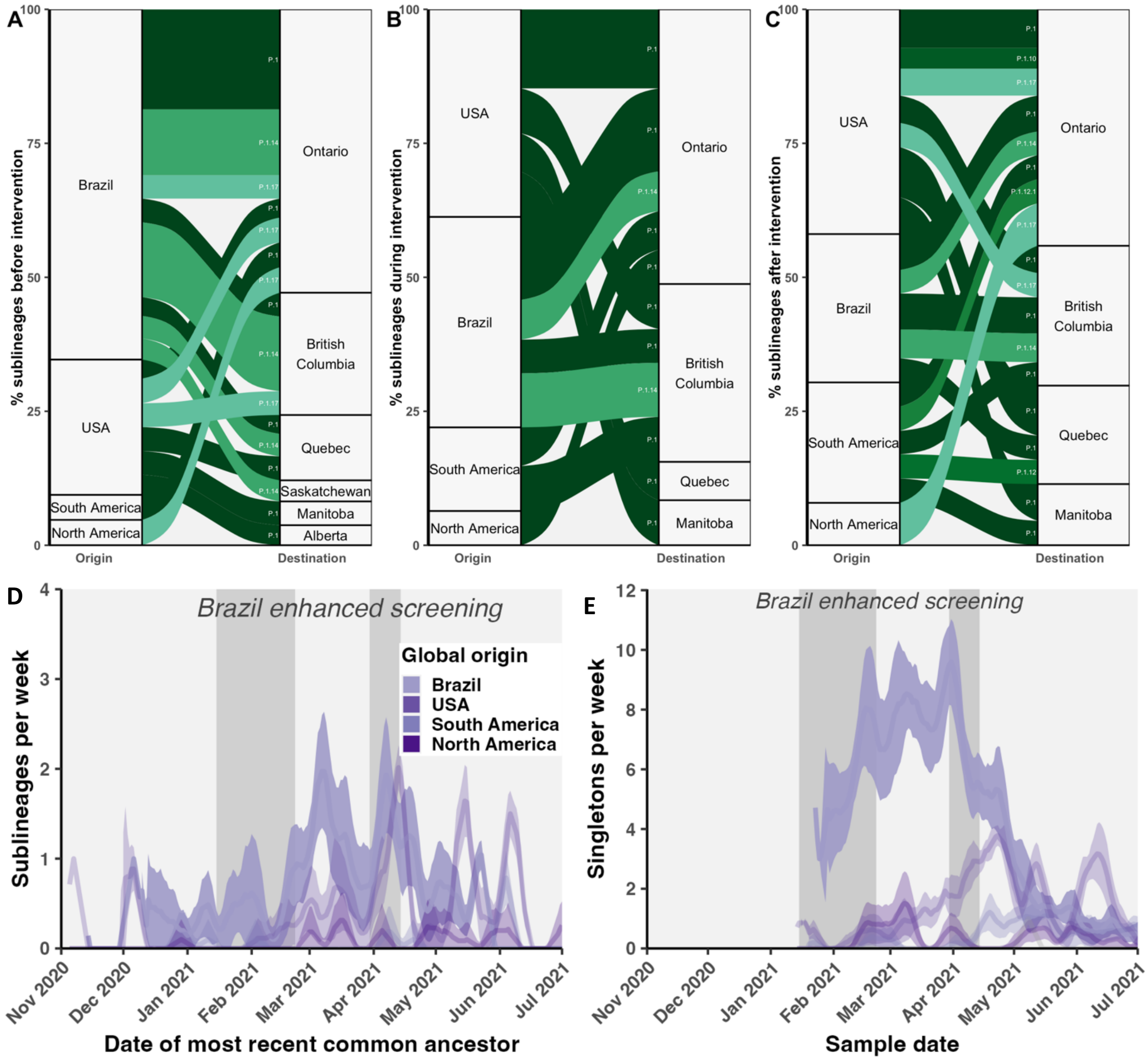

Fig. S46. Gamma introduction dynamics in Canada. Relative flows of Gamma sublineages, grouped by Pango lineage, from global origins to Canadian provinces **(A)** before, **(B)** during, and **(C)** after the Brazil enhanced screening restriction. **(D)** Gamma sublineages and **(E)** singletons introduced per week over time**.**

The second variant to be identified in Canada on November 24, 2020 was Zeta (P.2, alias: B.1.1.28.2), which was closely related to the Gamma variant and initially identified in Brazil as early as April 2020. As with Gamma, the majority of Zeta sublineages and singletons were imported from Brazil, with smaller contributions from the USA and Europe (Fig. S47).

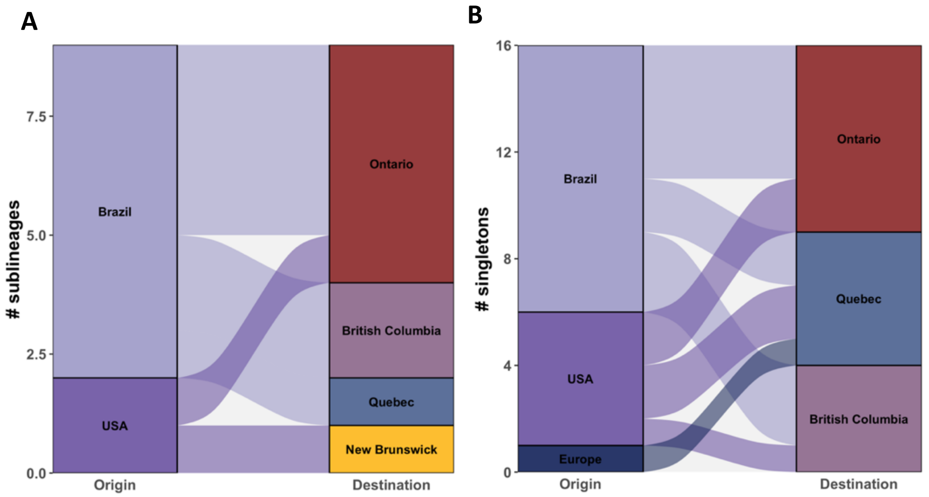

Fig. S47. Zeta (P.2) introduction dynamics in Canada. Origins and destinations of **(A)** sublineages and **(B)** singletons.

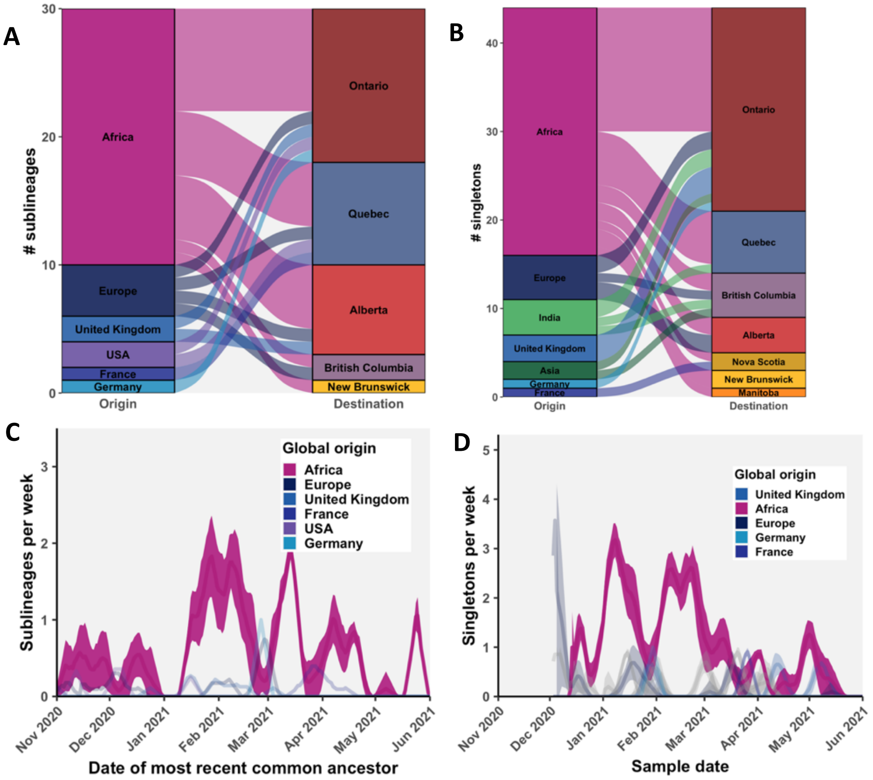

Fig. S48. **Eta (B.1.525) introduction dynamics in Canada.** Flows of **(A)** sublineages and **(B)** singletons overall, and **(C)**, **(D)** over time.

The Eta variant was first detected in the UK and Nigeria (Table S1). We estimated there were 24 (23 - 24) Eta sublineages imported into Canada between October 2020 and July 2021, resulting in 1762 (1759 - 1764) descendants sampled in Canada (Fig. S48). There were additionally 38 (36 - 41) Eta singletons introduced into Canada, accounting for 62% (60-63) of all Eta introductions.

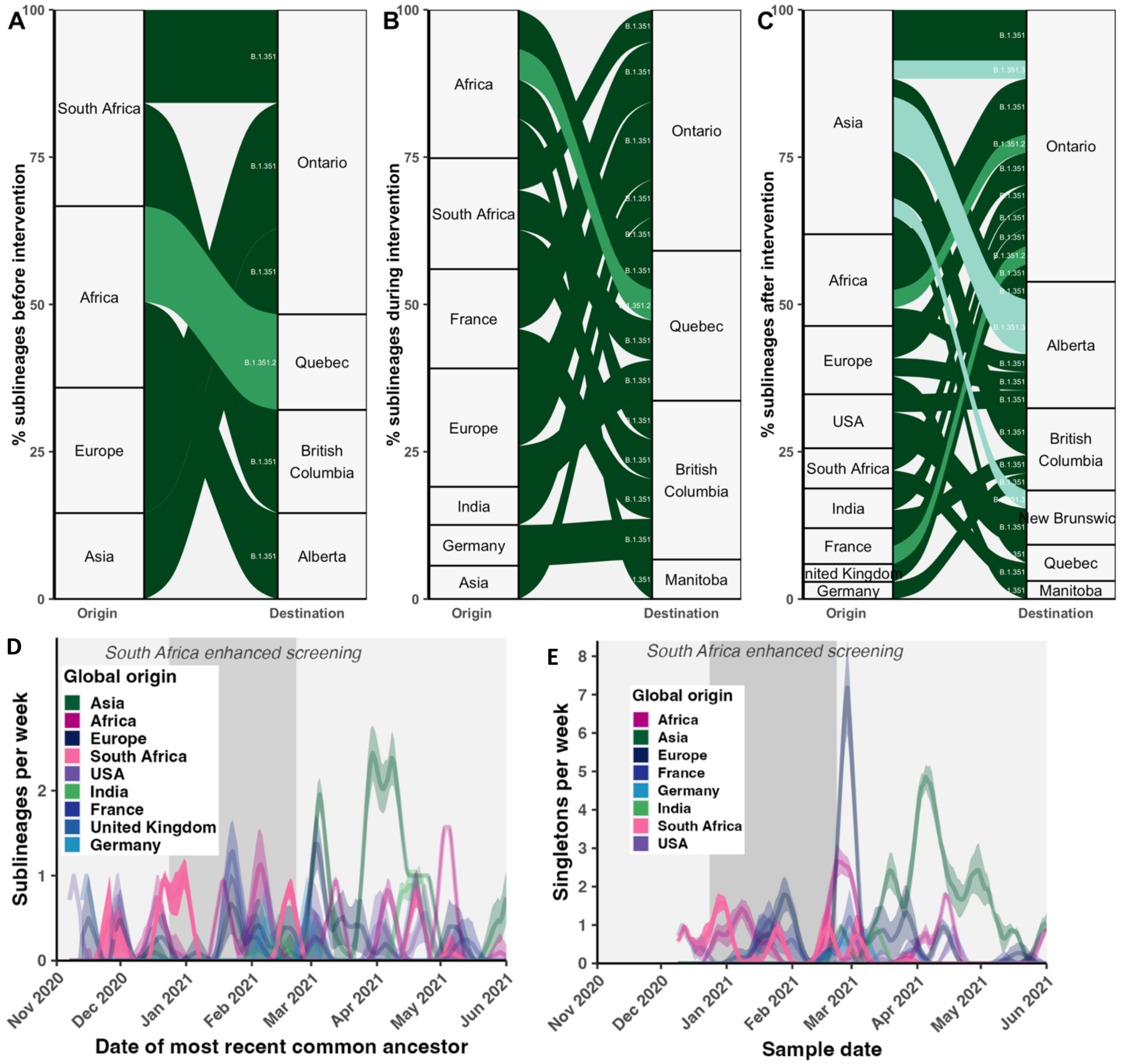

Fig. S49. **Beta (B.1.351) introduction dynamics in Canada.** Beta sublineage flows, grouped by Pango lineage, **(A)** before, **(B)** during, and **(C)** after the enhanced screening period for South Africa. **(D)** Beta sublineages and **(E)** singletons imported per week into Canada.

Epsilon consists of Pango lineages, B.1.429 and B.1.427, both were first detected in California, USA, with earliest sample dates in April 2020 for B.1.427 and in January 2020 for B.1.429 (cov-lineages.org, Table S1). The first detection in Canada was on December 5 2020. Our analysis suggests that Canada had at least 21 (20-22) B.1.429 and 6 (5-6) B.1.427 sublineages introduced between October 9, 2020 and April 12, 2021 (Fig. S50). All sublineages were inferred to have USA origins across all subsamples.

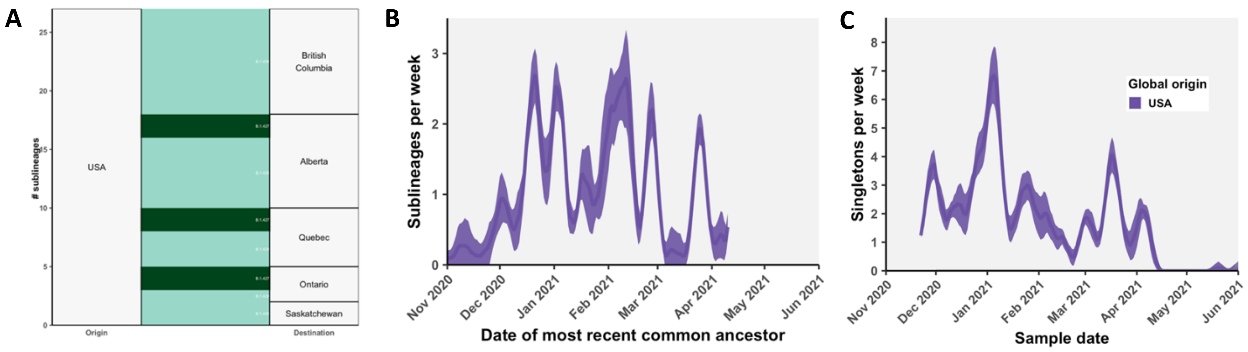

Fig. S50. **Epsilon (B.1.427, B.1.429) introduction dynamics in Canada. (A**) sublineage flows grouped by Pango lineage; **(B)** Epsilon sublineages and **(C)** singletons per week by global region of origin.

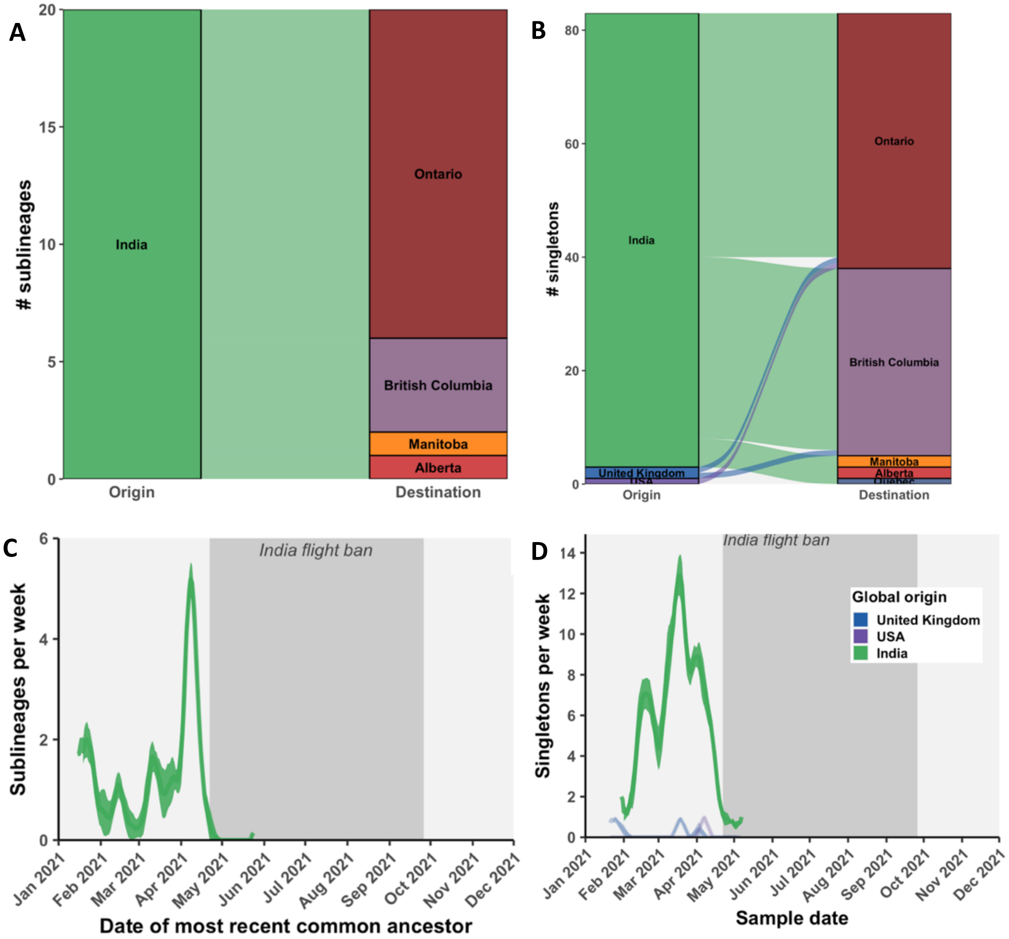

Fig. S51. **Kappa introduction dynamics in Canada.** Relative flows of Kappa **(A)** sublineages and **(B)** singletons. **(C)** Kappa sublineages and **(D)** singletons imported per week in the context of the Delta-associated India flight ban.

Kappa (B.1.617.1) was a sister lineage to Delta (B.1.617.2) identified in India around the same time. We estimated there were 21 (20 - 21) Kappa sublineages, entirely from India, and 81 (81 -

82) singletons, primarily from India but also the UK and US (Fig. S51). Kappa importation rates decreased drastically in association with India flight ban.

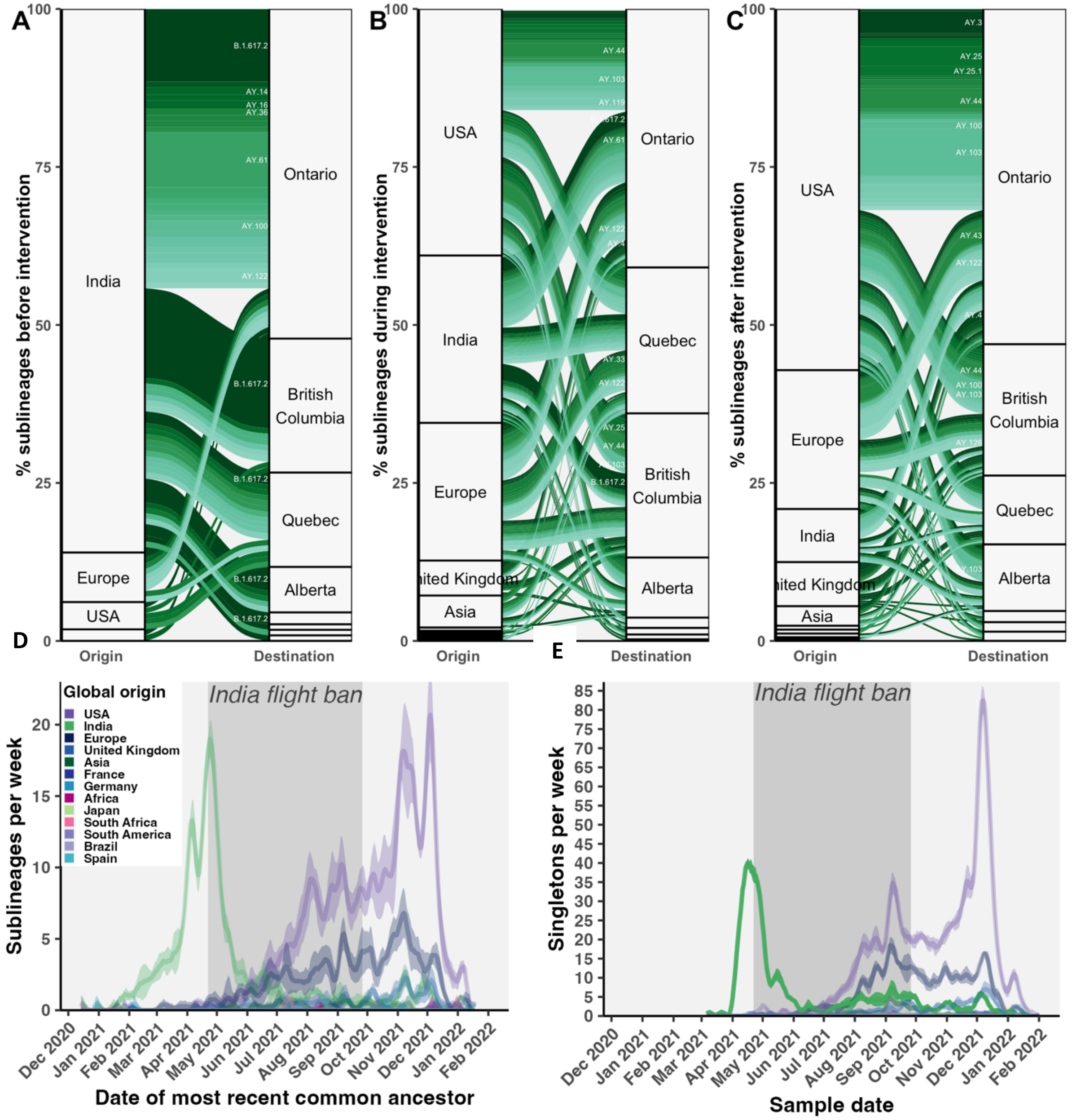

Fig. S52. Delta introduction dynamics in Canada. Relative contributions, grouped by Pango lineage, of global regions to Delta sublineages introduced to Canada **(A)** before, **(B)** during, and **(C)** after the flight ban. **(D)** Delta sublineages and **(E)** singletons introduced per week by global origin.

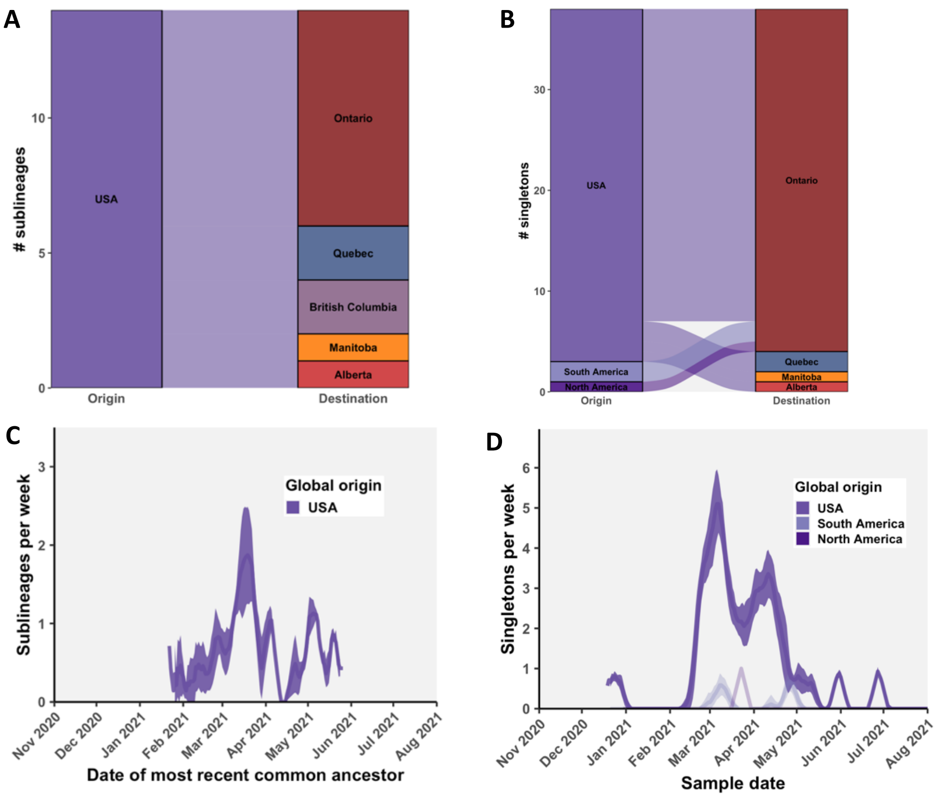

Fig. S53. Iota (B.1.526) introduction dynamics in Canada. Iota **(A)** sublineage and **(B)** singleton flows overall and (**C)** sublineages and **(D)** singletons introduced per week.

Iota was first identified in New York in early 2020. We inferred 14 (13 - 14) unique Iota sublineages and 37 (36 - 38) singletons introduced to Canada from December 2020 to July 2021 (Fig. S53), almost entirely from the USA and primarily into Ontario.

Mu was first identified in Colombia in December 2020 and first sampled in Canada in June 2021 (Table S1). There were at least 7 (7-7) Mu sublineages introduced to Canada and 55 (54 - 55) singletons (Fig. S54). Despite its first discovery in Colombia, 72% of Mu sublineages and 79% of singletons were inferred to have come from the USA, with 28% of sublineages and 11% of singletons from South America. Sequences from the USA comprised 45.1% of global sequences sampled, while 39.8% were from South America.

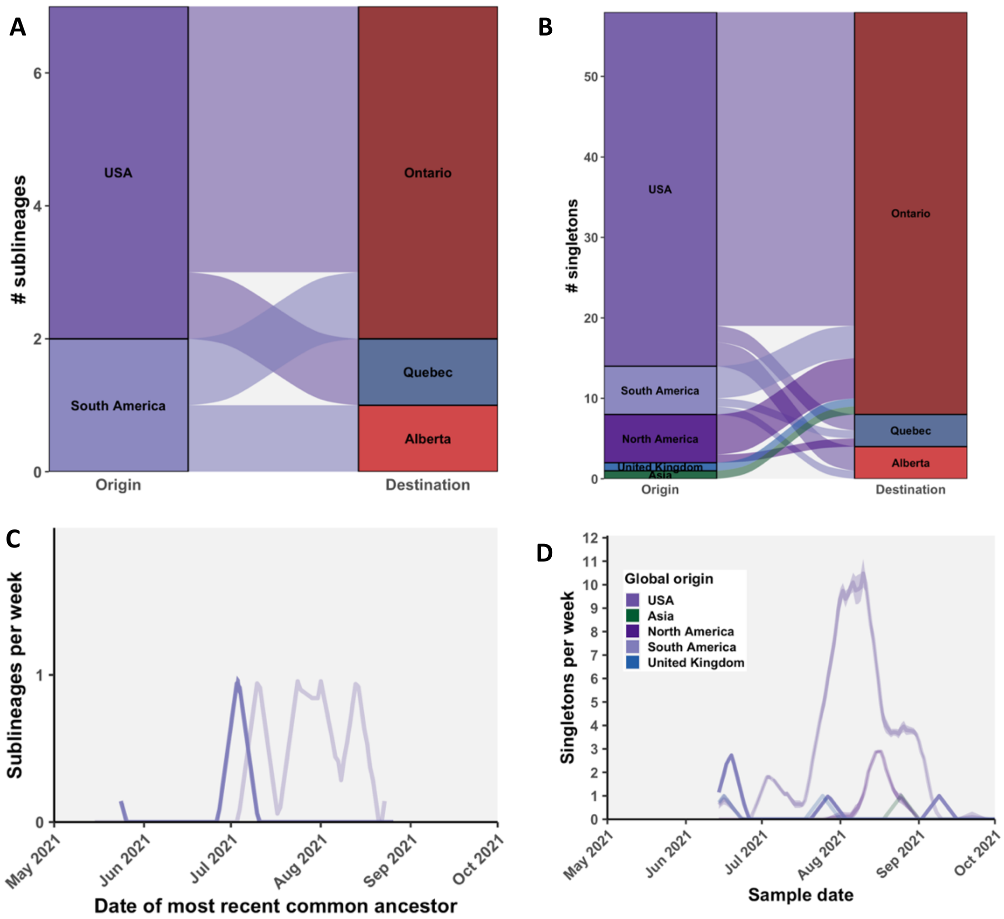

766 **Fig S54. Mu (B.1.621) introduction dynamics in Canada.** Mu **(A)** sublineage and **(B)** singleton flows overall,
767 and **(C)** sublineages per week and **(D)** singletons per week.

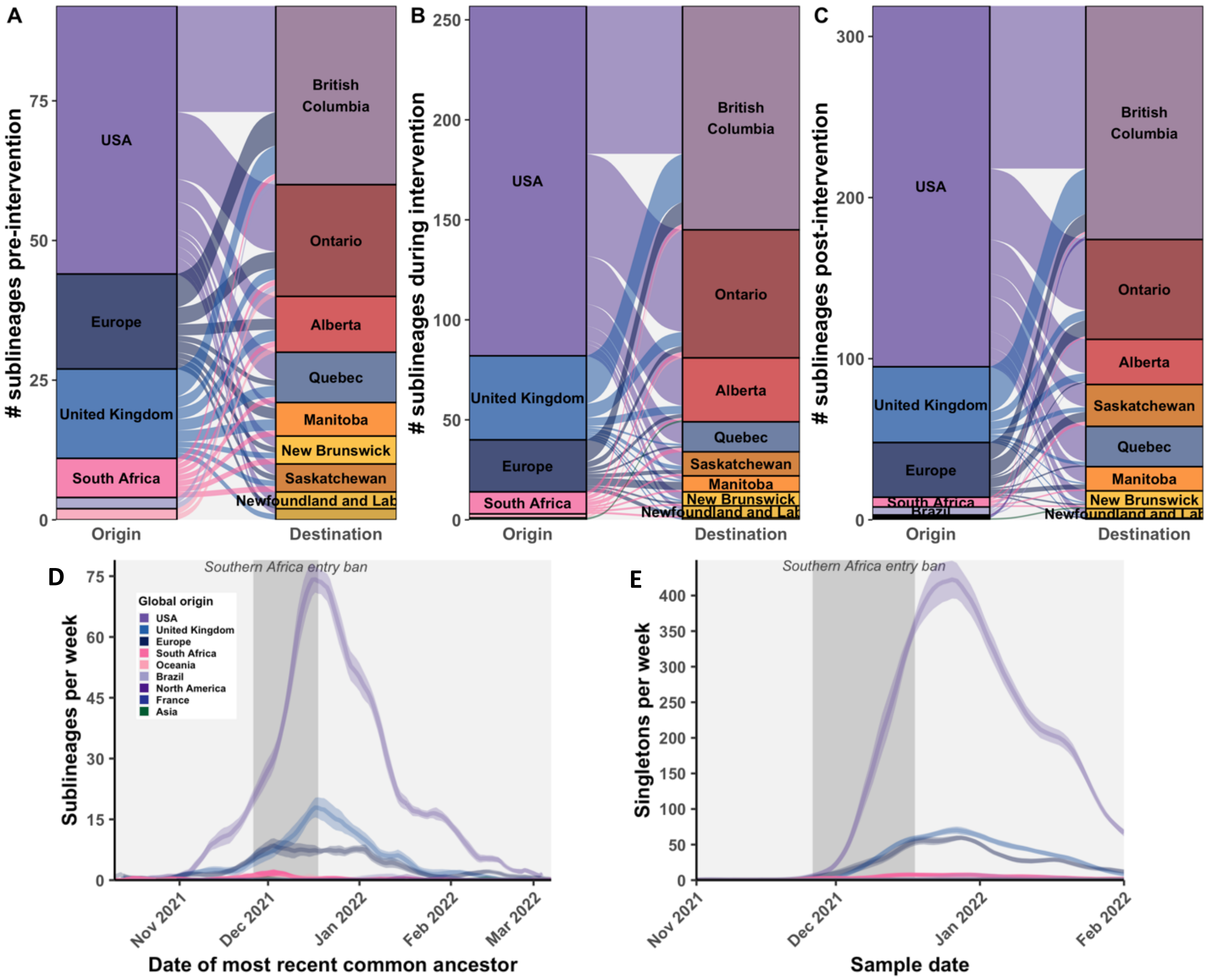

Fig. S55. Omicron BA.1 introduction dynamics in Canada. Relative contributions of global regions to BA.1 sublineages **(A)** before, **(B)** during, **(C)** and after the travel restrictions. **(D)** BA.1 sublineages and **(E)** singletons introduced per week, in the context of the southern Africa entry ban and enhanced screening intervention.

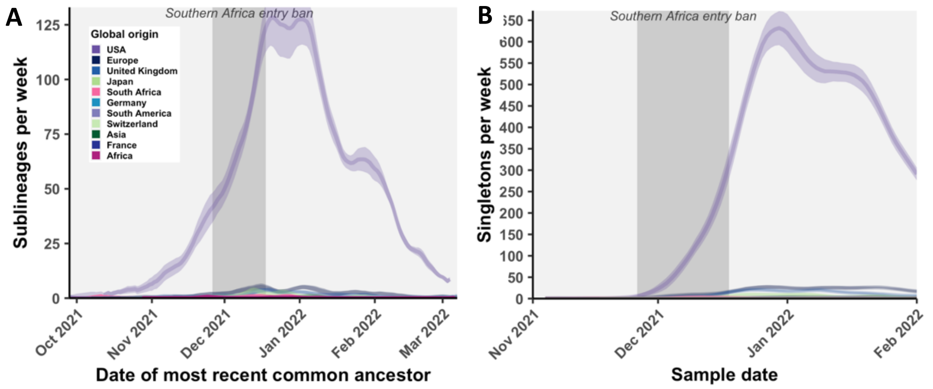

Fig. S56. Omicron BA.1.1 (A) sublineages and (B) singletons introduced per week.

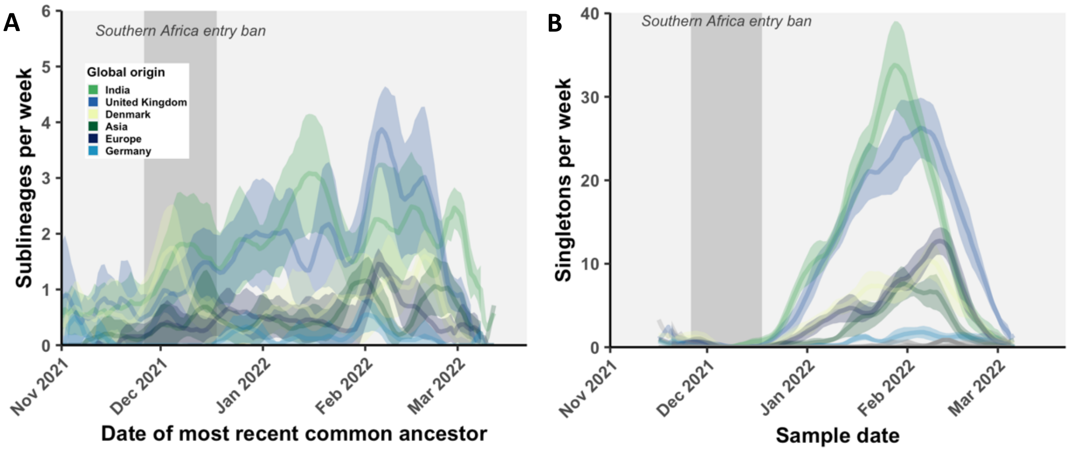

Fig. S57. Omicron BA.2 (A) sublineages and (B) singletons introduced per week.

	Before restrictions (-7 d)												
	Observed												
Variant	Diag	Intro	Intro/diag * 100										
Alpha	697.5	201.1	28.8										
Beta	18.0	17.8	99.1										
Gamma	0.0	4.2	Inf										
Delta	1011.4	386.8	38.2										
Omicron	10.5	499.3	4766.3										
	Early period restriction												
	Observed			Cases predicted			Intros predicted			Predicted intro/diag * 100			
Variant	Diag	Intro	Intro/diag * 100	Mean	Lower CI	Upper CI	Mean	Lower CI	Upper CI	Mean	Lower CI	Upper CI	
Alpha	158.8	256.6	161.5	167.1	165.9	168.3	273.9	269.4	278.5	163.9	162.4	165.5	
Beta	716.5	81.1	11.3	728.8	725.3	732.4	88.7	84.6	92.8	12.2	11.7	12.7	
Gamma	613.6	220.6	35.9	613.6	613.6	613.6	228.2	224.1	232.3	37.2	36.5	37.9	
Delta	29019.8	1382.6	4.8	29843.4	29807.3	29879.5	1810.0	1755.7	1864.2	6.1	5.9	6.2	
Omicron	489.9	2509.3	512.2	491.0	490.6	491.4	2509.6	2506.8	2512.5	511.1	511.0	511.2	
	Late period restriction												
	Observed			Cases predicted			Intros predicted			Predicted intro/diag * 100			
Variant	Diag	Intro	Intro/diag * 100	Mean	Lower CI	Upper CI	Mean	Lower CI	Upper CI	Mean	Lower CI	Upper CI	
Alpha	4317.3	338.1	7.8	4362.7	4358.6	4366.9	413.5	399.7	427.2	9.5	9.2	9.8	
Beta	1476.3	118.9	8.1	1514.1	1505.2	1523.0	116.5	112.9	120.1	7.7	7.5	7.9	
Gamma	23247.4	202.5	0.9	23247.4	23247.4	23247.4	202.5	197.6	207.4	0.9	0.8	0.9	
Delta	172500.2	3908.5	2.3	182830.0	182583.6	183076.4	4378.0	4317.7	4438.3	2.4	2.4	2.4	
Omicron	17978.0	8226.4	45.8	17982.4	17980.5	17984.2	8235.7	8226.5	8244.9	45.8	45.8	45.8	

Table S8. Ratios of importations to diagnosed variant cases before restrictions, and during the early and late periods of restrictions. For observed and predicted in the absence of restrictions.

References

Althaus CL. Ebola superspreading. *Lancet Infect Dis.* 2015;**15**(5):507–8.

Ayres DL, Darling A, Zwickl DJ, Beerli P, Holder MT, Lewis PO, et al. BEAGLE: an application programming interface and high-performance computing library for statistical phylogenetics. *Syst Biol.* 2012;**61**(1):170–3.

Baele G, Lemey P, Bedford T, Rambaut A, Suchard MA, Alekseyenko AV. Improving the accuracy of demographic and molecular clock model comparison while accommodating phylogenetic uncertainty. *Mol Biol Evol.* 2012a;**29**(9):2157–67.

Baele G, Li WLS, Drummond AJ, Suchard MA, Lemey P. Accurate Model Selection of Relaxed Molecular Clocks in Bayesian Phylogenetics. *Mol Biol Evol*. 2012b;**30**(2):239–43.

Canada Border Services Agency. Current travel restrictions—Standing Committee on Health: Emergency Situation Facing Canadians in Light of the COVID-19 Pandemic [Internet]. 2021a. Available from: www.cbsa-asfc.gc.ca/transparency-transparence/pd-dp/bbp-rpp/hesa/2021-05-21/travrestrict-restrictvoya-eng.html

Canada Border Services Agency. Timeline – CBSA Border Measures [Internet]. 2021b. Available from: www.publicsafety.gc.ca/cnt/trnsprnc/brfng-mtrls/prlmntry-bndrs/20211015/08-en.aspx

Canadian Institute for Health Information (CIHI). COVID-19 Intervention Timeline in Canada - Data Tables [Internet]. 2021 [cited 2021 Mar 26]. Available from: www.cihi.ca/en/covid-19-intervention-timeline-in-canada

Dougherty BP, Smith BA, Carson CA, Ogden NH. Exploring the percentage of COVID-19 cases reported in the community in Canada and associated case fatality ratios. *Infect Dis Model.* 2021;**6**:123–32.

Drummond AJ, Rambaut A. BEAST: Bayesian evolutionary analysis by sampling trees. *BMC Evol Biol*. 2007;**7**(1):214–8.

Endo A, Group C for the MM of IDC 19 W, Abbott S, Kucharski AJ, Funk S. Estimating the overdispersion in COVID-19 transmission using outbreak sizes outside China. *Wellcome Open Res.* 2020;**5**:67.

Faria NR, Mellan TA, Whittaker C, Claro IM, Candido D da S, Mishra S, et al. Genomics and epidemiology of the P.1 SARS-CoV-2 lineage in Manaus, Brazil. *Science.* 2021;**372**:815–21.

Ferreira MAR, Suchard MA. Bayesian analysis of elapsed times in continuous-time Markov chains. *Can J Stat.* 2008;**36**(3):355–68.

Gill MS, Lemey P, Faria NR, Rambaut A, Shapiro B, Suchard MA. Improving Bayesian Population Dynamics Inference: A Coalescent-Based Model for Multiple Loci. *Mol Biol Evol.* 2013;**30**(3):713–24.

Government of Canada. February 14, 2022 Declaration of Public Order Emergency [Internet]. 2022 [cited 2024 Apr 11]. Available from: www.justice.gc.ca/eng/csj-sjc/section58.html

Grubaugh ND, Ladner JT, Kraemer MUG, Dudas G, Tan AL, Gangavarapu K, et al. Genomic epidemiology reveals multiple introductions of Zika virus into the United States. *Nature.* 2017 May 24;**546**:401–5.

Health Canada. Health Canada authorizes first COVID-19 vaccine [Internet]. 2020 [cited 2024 Apr 11]. Available from: www.canada.ca/en/health-canada/news/2020/12/health-canada-authorizes-first-covid-19-vaccine0.html

Hill V, Plessis LD, Peacock TP, Aggarwal D, Colquhoun R, Carabelli AM, et al. The origins and molecular evolution of SARS-CoV-2 lineage B.1.1.7 in the UK. *Virus Evol.* 2022;**8**(2):veac080.

Lemey P, Rambaut A, Drummond AJ, Suchard MA. Bayesian phylogeography finds its roots. *PLoS Comp Biol.* 2009;**5**(9):e1000520.

McLaughlin A, Montoya V, Miller RL, Mordecai GJ, Consortium CC 19 GN (CanCOGen), Worobey M, et al. Genomic epidemiology of the first two waves of SARS-CoV-2 in Canada. *Elife.* 2022;11.

Minin VN, Bloomquist EW, Suchard MA. Smooth Skyride through a Rough Skyline: Bayesian Coalescent-Based Inference of Population Dynamics. *Mol Biol and Evol*. 2008;**25**(7):1459–71.

Minin VN, Suchard MA. Counting labeled transitions in continuous-time Markov models of evolution. *J Math Biol.* 2008;**56**(3):391–412.

Nishiura H, Linton NM, Akhmetzhanov AR. Serial interval of novel coronavirus (COVID-19) infections. *Intl J of Infect Dis*. 2020;**93**:1–14.

Piper J, Gomis B, Lee K. "Guided by Science and Evidence"? The Politics of Border Management in Canada's Response to the COVID-19 Pandemic. *Frontiers Political Sci*. 2022;**4**:834223.

Public Health Agency of Canada (PHAC). Canada suspends flights from the United Kingdom for 72 hours [Internet]. 2020. Available from: www.canada.ca/en/public-health/news/2020/12/canada-suspends-flights-from-the-united-kingdom-for-72-hours.html

PHAC. Government of Canada's first phase to easing border measures for travellers entering Canada [Internet]. 2021a [cited 2023 May 10]. Available from: www.canada.ca/en/public-health/news/2021/06/government-of-canadas-first-phase-to-easing-border-measures-for-travellers-entering-canada3.html

PHAC. Adjustments to Canada's border and travel measures [Internet]. 2021b [cited 2023 May 10]. Available from: www.canada.ca/en/public-health/news/2021/11/adjustments-to-canadas-border-and-travel-measures.html

PHAC. Government of Canada introduces additional measures to address COVID-19 Omicron variant of concern (November 30 2021) [Internet]. 2021c. Available from: www.canada.ca/en/public-health/news/2021/11/government-of-canada-introduces-additional-measures-to-address-covid-19-omicron-variant-of-concern.html

PHAC. Government of Canada introduces additional measures to address COVID-19 Omicron variant of concern (December 17 2021) [Internet]. 2021d. Available from: www.canada.ca/en/public-health/news/2021/12/government-of-canada-announces-additional-measures-to-contain-the-spread-of-the-omicron-variant.html

PHAC. Coronavirus disease 2019 (COVID-19): Epidemiology update [Internet]. Ottawa, ON: Government of Canada; 2021e Feb. Available from: health-infobase.canada.ca/covid-19/epidemiological-summary-covid-19-cases.html?stat=num&measure=active#a2

PHAC. Government of Canada announces additional measures to contain the spread of the Omicron variant [Internet]. 2021f. Available from: www.canada.ca/en/public-health/news/2021/12/government-of-canada-announces-additional-measures-to-contain-the-spread-of-the-omicron-variant.html

PHAC. Requirements for truckers entering Canada in effect as of January 15, 2022 [Internet]. 2022a [cited 2024 Apr 11]. Available from: www.canada.ca/en/public-health/news/2022/01/requirements-for-truckers-entering-canada-in-effect-as-of-january-15-2022.html

PHAC. Government of Canada lightens border measures as part of transition of the pandemic response [Internet]. 2022b. Available from: Government of Canada lightens border measures as part of transition of the pandemic response. www.canada.ca/en/public-health/news/2022/02/government-of-canada-lightens-border-measures-as-part-of-transition-of-the-pandemic-response.html

PHAC. Government of Canada will remove pre-entry test requirements for vaccinated travellers on April 1 [Internet]. 2022c [cited 2023 May 10]. Available from: www.canada.ca/en/public-health/news/2022/03/government-of-canada-will-remove-pre-entry-test-requirement-for-fully-vaccinated-travellers-on-april-1.html

PHAC. Government of Canada announces additional easing of border measures effective April 25 [Internet]. 2022d [cited 2023 May 10]. Available from: www.canada.ca/en/public-health/news/2022/04/government-of-canada-announces-additional-easing-of-border-measures-effective-april-25.html

PHAC. Government of Canada to remove COVID-19 border and travel measures effective October 1 [Internet]. 2022e [cited 2024 Apr 11]. Available from: www.canada.ca/en/public-health/news/2022/09/government-of-canada-to-remove-covid-19-border-and-travel-measures-effective-october-1.html

Rambaut A, Drummond AJ, Xie D, Baele G, Suchard MA. Posterior summarization in Bayesian phylogenetics using Tracer 1.7. *Syst Biol.* 2018;**67**(5):901–4.

Rambaut A, Lam TT, Carvalho LM, Pybus OG. Exploring the temporal structure of heterochronous sequences using TempEst (formerly Path-O-Gen). *Virus Evol*. 2016;**2**(1):vew007-7.

Reichmuth ML, Hodcroft EB, Riou J, Neher RA, Hens N, Althaus CL. Impact of cross-border-associated cases on the SARS-CoV-2 epidemic in Switzerland during summer 2020 and 2021. *Epidem*. 2022;**41**:100654.

Riou J, Althaus CL. Pattern of early human-to-human transmission of Wuhan 2019 novel coronavirus (2019-nCoV), December 2019 to January 2020. *Eurosurv.* 2020;**25**(4):2000058.

Russell TW, Golding N, Hellewell J, Abbott S, Wright L, Pearson CAB, et al. Reconstructing the early global dynamics of under-ascertained COVID-19 cases and infections. *BMC Med.* 2020;18(1):332.

Statistics Canada. 2021 Census Population and dwelling counts: Canada, provinces and territories [Internet]. 2022 [cited 2023 Jul 19]. Available from: www150.statcan.gc.ca/t1/tbl1/en/tv.action?pid=9810000101

Statistics Canada. Table 24-10-0056-01 Leading indicator, International visitors entering or returning to Canada by air, by country of residence and traveller type [Internet]. 2023 [cited 2023 Apr 5]. Available from: doi.org/10.25318/2410005601-eng

Suchard MA, Lemey P, Baele G, Ayres DL, Drummond AJ, Rambaut A. Bayesian phylogenetic and phylodynamic data integration using BEAST 1.10. *Virus Evol*. 2018;**4**(1):vey016.

Susvitasari K, Tupper P, Stockdale JE, Colijn C. A method to estimate the serial interval distribution under partially-sampled data. *Epidem.* 2023;100733.

Transport Canada. Expansion of international flight restrictions at Canadian airports [Internet]. 2021a [cited 2024 Apr 11]. Available from: www.canada.ca/en/transport-

canada/news/2021/01/expansion-of-international-flight-restrictions-at-canadian-airports.html

Transport Canada. Government of Canada suspends flights from India and Pakistan [Internet]. 2021b. Available from: www.canada.ca/en/transport-canada/news/2021/04/government-of-canada-suspends-flights-from-india-and-pakistan.html

Transport Canada. Transport Canada extending restrictions on direct flights from India until September 21, 2021 [Internet]. 2021c. Available from: www.canada.ca/en/transport-canada/news/2021/08/transport-canada-extending-restrictions-on-direct-flights-from-india-until-september-21-2021.html

Transport Canada. Transport Canada suspending all direct commercial and private passenger flights from Morocco until September 29, 2021 [Internet]. 2021d [cited 2024 Apr 22]. Available from: www.canada.ca/en/transport-canada/news/2021/08/transport-canada-suspending-all-direct-commercial-and-private-passenger-flights-from-morocco-until-september-29-2021.html

Transport Canada. Canada announces extension of flight ban from India as it prepares for the return of direct flights [Internet]. 2021e. Available from: www.canada.ca/en/transport-canada/news/2021/09/canada-announces-extension-of-flight-ban-from-india-as-it-prepares-for-the-return-of-direct-flights.html

Viana R, Moyo S, Amoako DG, Tegally H, Scheepers C, Althaus CL, et al. Rapid epidemic expansion of the SARS-CoV-2 Omicron variant in southern Africa. *Nature*. 2022;**603**:679–86.
