# Supplementary material for: Phylogeographic evaluation of the effectiveness of Canadian travel restrictions in reducing SARS-CoV-2 variant importations and burden
Source: Virus Evol. 2025 Oct 11;11(1):veaf077. doi: 10.1093/ve/veaf077 (PMC12526992; doi:10.1093/ve/veaf077)
Supplement: Supp_CanCOGeN-consortium-authorship_veaf077 [file supp_cancogen-consortium-authorship_veaf077.pdf]

The Canadian COVID-19 Genomics Network (CanCOGeN) consortium authorship

Steering Committee

Lorne Hepworth, Kenneth Baillie, Bettina Hamelin, Rob Annan, Ewan Harrison, Duncan MacCannell, Chris McMaster, Allison McGeer, Eric Meslin, Howard Njoo, Joris Veltman

Coordinating Committee

Catalina Lopez-Correa, Naveed Aziz, Fiona Brinkman, William Hsiao, Yann Joly, Steven Jones, Sandrine Moreira, Samira Mubareka, Natalie Prystajecky, Terrance Snutch, Lisa Strug, Gary Van Domselaar, Ma'an Zawati, David Alexander, Erin Gill, Jordan Lerner-Ellis, Calvin Sjaarda, Guillaume Butler-Laporte, Paul McLaren, Paul Vancaeseele, Diana Iglesias, Agnes Baross, Koko Agborsangaya, Megan Smallwood, Yoo Jin Park, Marc-André Langlois, Gerald Pfeffer

Data Sharing Committee

Art Poon, Fiona Brinkman, Catalina Lopez-Correa, Emma Griffiths, Eric Sutherland, , Gary van Domselaar, Gijs van Rooijen, Guillaume Bourque, Kieran O'Doherty, Kimberlyn McGrail, Jason Leblanc, Lisa Strug, Ma'n Zawati, Marc Fiume, Matthew Croxen, Michael Szego, Natalie Prystajecky, Sandrine Moreira, Steven Jones, Terrance Snutch, Will Hsiao, Yann Joly, Erin Gill Megan Smallwood, Eddy Nason, Koko Agborsangaya, Daryl Waggott.

Canadian Public Health Laboratory Network members and staffs having contributed data

Cadham Provincial Laboratory (Manitoba); National Microbiology Laboratory

Anna Majer, Shari Tyson, Grace Seo, Philip Mabon, Elsie Grudeski, Rhiannon Huzarewich, Russell Mandes, Anneliese Landgraff, Jennifer Tanner, Natalie Knox, Morag Graham, Gary Van Domselaar, Paul Van Caeseele, Jared Bullard, David Alexander, Kerry Dust, Nathalie Bastien, Yan Li, Timothy Booth, Darian Hole, Madison Chapel, Kirsten Biggar, Adam Hedley, Ana Duggan, Ayooluwa Bolaji, Brooke Cistarelli, Catherine Yoshida, Vanessa Laminman, Christine Bonner, Erika (Rikki) Landry, Katrina (Katie) Lanyon, Brynn Kaplen

Hôpital Georges L. Dumont (New Brunswick); National Microbiology Laboratory

Anna Majer, Shari Tyson, Grace Seo, Philip Mabon, Elsie Grudeski, Rhiannon Huzarewich, Russell Mandes, Anneliese Landgraff, Jennifer Tanner, Natalie Knox, Morag Graham, Gary Van Domselaar, Richard Garceau, Guillaume Desnoyers, Nathalie Bastien, Yan Li, Timothy Booth, Darian Hole, Madison Chapel, Kirsten Biggar, Pierre Lyons, Annie-Pier Beauregard, Woodson Shaw, Genevieve Labbe, Catherine Yoshida, Vanessa Laminman, Christine Bonner, Erika (Rikki) Landry, Katrina (Katie) Lanyon, Brynn Kaplen

Alberta Precision Labs; University of Alberta; University of Calgary

Berenger B, Bernier F, Buss, E, Chui L, Croxen M, Deo A, Dieu P, Gill K, Ferrato C, Gordon P, Kellner J, Khan F, Koleva P, Lam LG, Li V, Lloyd C, Lynch T, Ma R, Melin A, Murphy S, Pabbaraju K, Shokoples S, Tipples G, Thayer J, Whitehouse M, Wong A, Yu C, Zelyas N, Colin Lloyd, Petya Koleva, Johanna Thayer, Andrea Tyler, Patrick Bastedo, Catherine Yoshida

Laboratoire de Santé Publique du Québec, McGill Génome Sciences Centre; CoVSeq Consortium

Sandrine Moreira, Jiannis Ragoussis, Guillaume Bourque, Jesse Shapiro, Éric Fournier, Réjean Dion, Hugues Charest, Aurélie Guilbault, Benjamin Delisle, Sarah Reiling, Anne-Marie Roy, Shu-Huang Chen, Corinne Darmond, Sally Lee, Brent Brookes, Pierre Lepage, Jannick St-Cyr, Patrick Willet, Mathieu Bourgey, David Bujold, Hector Galvez, Paul Stretenowich, Pierre-Olivier Quirion, Romain Grégoire, Carmen Lia Murall, Julie Hussin, Raphaël Poujol, Jean-Christophe Grenier, Fatima Mostefai, Sylvie Laboissières, Alexandre Montpetit, Mark Lathrop, Michel Roger, Judith Fafard, Florence Lacasse, Martine Isabelle, Inès Levade, Bouchra Serhir, Joel Menard, Martine Morin, Kodjovi Mlaga, Ines Levade, Genevieve Labbe, Catherine Yoshida

McMaster University

Hooman Derakhshani, Sheridan J.C. Baker, Emily M. Panousis, Ahmed N. Draia, Jalees A. Nasir, Michael G. Surette, Andrew G. McArthur

Toronto Invasive Bacterial Diseases Network; Sunnybrook Health Sciences

Allison McGeer, Patryk Aftanas, Angel Li, Kuganya Nirmalarajah, Emily Panousis, Ahmed Draia, Jalees Nasir, David Richardson, Michael Surette, Samira Mubareka, Andrew G. McArthur

Eastern Ontario Regional Laboratory Association

Leanne Mortimer, Hooman Derakhshani, Emily Panousis, Ahmed Draia, Jalees Nasir, Robert Slinger, Andrew G. McArthur

Public Health Ontario Laboratory

Vanessa G Allen, Yao Chen, Richard de Borja, Alireza Eshaghi, Nahuel Fittipaldi, Christine Frantz, Jonathan B Gubbay, Jennifer L Guthrie, Lawrence Heisler, Esha Joshi, Michael Laszloffy, Michael C.Y. Li, Sandeep Nagra, Yogi Sundaravadanam, Matthew Watson, Sandra Zittermann, Jacob Afelskie, Doonia Bajovic, Philip Banh, Claudia Chu, Kirby Cronin, Rachelle DiTullio, Carla Duncan, Dhiraj Gaglani, Mark Horsman, Hadia Hussain, Jason Iraheta, Grace Jeong, Sushma Kavikondala, Lisa Kim, Surendra Kumar, Aimin Li, Maria Mariscal, Alex Marchand-Austin, Dean Maxwell, Lisa McTaggart, Fatima Merza, Esther Nagai, Rima Palencia, John Palmer, Samir N Patel, Nataliya Potapova, Anna Puzinovici, Zarah Rajaei, Himeshi Samarsinghe, Candice Schreiber, Narisha Shakuralli, Christine Seah, Karthikeyan Sivaraman, Fatemeh Shaeri, Vincent Su Bin Cha, Ashleigh Sullivan, Sarah Teatero, Semra Tibebu, Nobish Varghese, Andre Villegas, Jesse Wang, Sophie Yu, Farhan Yusuf, Genevieve Labbe, Catherine Yoshida

Ontario Institute of Cancer Research

Jared Simpson

Mount Sinai Hospital

Jeff Wrana

BC-CDC Public Health Laboratory

GenomeCanada

CanCOGeN

Prystajecky Natalie, Linda Hoang, John R. Tyson, Dan Fornika, Shannon Russell, Kim Macdonald, Kimia Kamelian, Ana Pacagnella, Corrinne Ng, Loretta Janz, Richard Harrigan, Robert Azana, Mel Krajden, Jessica M. Caleta, Tara Newman, Kimia Kamelian, Andrea Tyler, Patrick Bastedo, Catherine Yoshida

University of British Columbia

John R. Tyson, Terrance P. Snutch

Roy Romanow Provincial Laboratory (Saskatchewan)

Ryan McDonald, Keith MacKenzie, Meredith Faires, Kara Loos, Stefani Kary, Rachael DePaulo, Alanna Senecal, Amanda Lang, Ana Duggan, Catherine Yoshida, Jessica Minion

Public Health Laboratory (Newfoundland) - Dr. Leonard A. Miller Centre for Health Services; National Microbiology Laboratory

Anna Majer, Shari Tyson, Grace Seo, Philip Mabon, Elsie Grudeski, Rhiannon Huzarewich, Russell Mandes, Anneliese Landgraff, Jennifer Tanner, Natalie Knox, Morag Graham, Gary Van Domselaar, Robert Needle, Yang Yu, Adel Malek, Laura Gilbert, George Zahariadis, Nathalie Bastien, Yan Li, Timothy Booth, Darian Hole, Madison Chapel, Kirsten Biggar, Kerri Smith, Matthew Gilmour, Chris Corkum, Geoff Woodland, Phillip Andrews, Ana Duggan, Catherine Yoshida, Vanessa Laminman, Christine Bonner, Erika (Rikki) Landry, Katrina (Katie) Lanyon, Brynn Kaplen

QEII Health Sciences Centre (Nova Scotia); National Microbiology Laboratory

Anna Majer, Shari Tyson, Grace Seo, Philip Mabon, Elsie Grudeski, Rhiannon Huzarewich, Russell Mandes, Anneliese Landgraff, Jennifer Tanner, Natalie Knox, Morag Graham, Gary Van Domselaar, Todd Hatchette, Jason LeBlanc, Janice Pettipas, Dan Gaston, Nathalie Bastien, Yan Li, Timothy Booth, Darian Hole, Madison Chapel, Kirsten Biggar, Allana Loder, Greg McCracken, Greg McCracken, Allana Loder, Genevieve Labbe, Catherine Yoshida, Vanessa Laminman, Christine Bonner, Erika (Rikki) Landry, Katrina (Katie) Lanyon, Brynn Kaplen

CanCOGeN VirusSeq committees and working groups' members:

CanCOGeN VirusSeq Implementation Committee

GenomeCanada

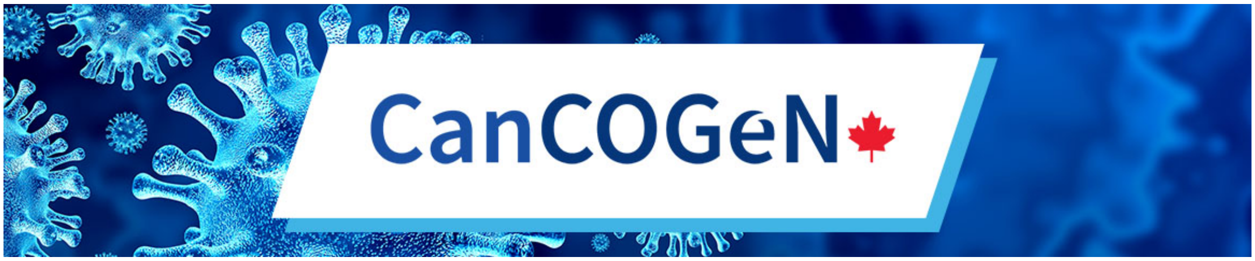

Terrance Snutch, Fiona Brinkman, Marceline Côté, William Hsiao, Yann Joly, Sharmistha Mishra, Sandrine Moreira, Samira Mubareka, Jared Simpson, Gary Van Domselaar

CanCOGeN Capacity Building Working Group

Gary Van Domselaar, Matthew Croxen, Natalie Knox, Celine Nadon, Hezhao Ji, Jennifer Tanner, Catherine Yoshida

CanCOGeN Data Analytics Working Group

Gary Van Domselaar, Fiona Brinkman, Zohaib Anwar, Robert Beiko, Matieu Bourgey, Guillaume Bourque, Ahmed Draia, Jun Duan, Marc Fiume, Dan Fornika, Eric Fournier, Erin Gill, Paul Gordon, Emma Griffiths, Jose Hector Galvez Lopez, Darian Hole, Will Hsiao, Jeffrey Joy, Kimia Kamelian, Natalie Knox, Philip Mabon, Finlay Maguire, Tom Matthews, Andrew McArthur, Samir Mechai, Sandrine Moreira, Art Poon, Amos Raphenya, Claire Sevenhuysen, Jared Simpson, Jennifer Tanner, Lauren Tindale, John Tyson, Geoff Winsor, Nolan Woods, Genevieve Labbe, Ana Duggan, Andrea Tyler, Patrick Bastedo, Catherine Yoshida

CanCOGeN Ethics and Governance Working Group

Yann Joly, Fiona Brinkman, Erin Gill, Will Hsiao, Hanshi Liu, Sandrine Moreira, Gary Van Domselaar, Ma'n Zawati, Sarah Savić-Kallesøe

CanCOGeN Metadata Working Group

William Hsiao, David Alexander, Zohaib Anwar, Nathalie Bastien, Tim Booth, Guillaume Bourque, Fiona Brinkman, Hughes Charest, Caroline Colijn, Matthew Croxen, Guillaume Desnoyers, Rejean Dion, Damion Dooley, Ana Duggan, Leah Dupasquier, Kerry Dust, Nahuel Fittipaldi, Eleni Galanis, Emma Garlock, Greg German, Erin Gill, Gurinder Gopal, Tom Graefenhan, Morag Graham, Emma Griffiths, Linda Hoang, Naveed Janjua, Jeffrey Joy, Kimia Kamelian, Lev Kearney, Natalie Knox, Theodore Kuschak, Jason LeBlanc, Yan Li, Anna Majer, Adel Malek, Dionne Marcino, Ryan McDonald, David Moore, Celine Nadon, Samir Patel, Natalie Prystajecky, Anoosha Sehar, Claire Sevenhuysen, Garrett Sorensen, Laura Steven, Lori Strudwick, Jennifer Tanner, Marsha Taylor, Shane Thiessen, Gary Van Domselaar, Adrian Zetner

CanCOGeN Research Collaborations Working Group

Fiona Brinkman, Zohaib Anwar, Marceline Côté, Marc Fiume, Laura Gilbert, Erin Gill, Paul Gordon, Yann Joly, Sandrine Moreira, Samira Mubareka, Natalie Prystajecky, Jennifer Tanner, Gary Van Domselaar, Phot Zahariadis,

CanCOGeN Sequencing Working Group

Ioannis Ragoussis, Terrance Snutch, Patryk Aftanas, Matthew Croxen, Hooman Derakhshani, Nahuel Fittipaldi, Morag Graham, Andrew McArthur, Sandrine Moreira, Samira Mubareka, Natalie Prystajecky, Ioannis Ragoussis, Jared Simpson, Michael Surette, John Tyson, Genevieve Labbe, Ana Duggan, Andrea Tyler, Patrick Bastedo, Catherine Yoshida, Vanessa Laminman, Christine Bonner, Erika (Rikki) Landry, Katrina (Katie) Lanyon, Brynn Kaplen

Canadian VirusSeq Data Portal (CVDP) Team

Guillaume Bourque, Lincoln Stein, Christina Yung, Hanshi Liu, Yann Joly, Adrielle Houweling, William Hsiao, Marc Fiume, David Bujold, Erin Gill, Fiona Brinkman

CanCOGeN Modeling Working Group

Samira Mubareka, Catalina Lopez-Correa, Erin Gill, Natalie Prystajecky, Beate Sander, Sarah P. Otto, Gary Van Domselaar, Guillaume Polliquin, Sandrine Moreira, Sharmistha Mishra, Caroline Colijn, Koko Agborsangaya, Troy Day, Micheal Li, Art F.Y. Poon, Jeffrey B. Joy, Marc Brisson, David Earn, Mathieu Maheu-Giroux, Julien Arino, Fiona Brinkman
